# Virtual reality-based emotion-to-emotion therapy for post-traumatic stress disorder: Protocol for a randomized controlled trial of efficacy, safety, and cost-effectiveness

**DOI:** 10.1371/journal.pone.0357593

**Published:** 2026-09-02

**Authors:** Do-Eun Lee, Namju Lee, Nam-Kwen Kim, Joohee Seo, Jaeuk U. Kim, In Chul Jung, Moon Joo Cheong, Hyung Won Kang

**Affiliations:** 1 Department of Korean Neuropsychiatry Medicine, College of Korean Medicine, Wonkwang University, Iksan, Jeonbuk, Republic of Korea; 2 Korean Medicine Cognitive Disorder Research Center, College of Korean Medicine, Wonkwang University, Iksan, Jeonbuk, Republic of Korea; 3 Department of Integrative Medicine, Division of Biomedical and Health Service Convergence, Graduate School of Jeonbuk Advanced Bio-convergence Academy (JABA), Wonkwang University, Iksan, Jeonbuk, Republic of Korea; 4 Department of Ophthalmology and Otolaryngology and Dermatology, School of Korean Medicine, Pusan National University, Yangsan, Gyeongnam, Republic of Korea; 5 Department of Korean Neuropsychiatry, National Medical Center, Seoul, Republic of Korea; 6 Digital Health Research Division, Korea Institute of Oriental Medicine, Daejeon, Republic of Korea; 7 Department of Oriental Neuropsychiatry, College of Korean Medicine, Daejeon University, Daejeon, Republic of Korea; 8 Clinical Trial Center, Daejeon Korean Medicine Hospital of Daejeon University, Daejeon, Republic of Korea; 9 Department of Medical Counseling, College of Health Sciences, Wonkwang University, Iksan, Jeonbuk, Republic of Korea; National Cerebral and Cardiovascular Center: Kokuritsu Junkankibyo Kenkyu Center, JAPAN

## Abstract

**Background:**

Trauma-focused psychotherapies are recommended first-line treatments for post-traumatic stress disorder (PTSD). Emotion-to-Emotion Therapy (ETE) is a psychotherapy derived from traditional Korean medicine that regulates excessive emotional responses using theoretically opposing emotions. Virtual reality (VR) may support standardized and immersive delivery of the meditation component of ETE. This trial will evaluate the efficacy, safety, and cost-effectiveness of VR-based ETE for PTSD.

**Methods:**

This multicenter, randomized, waitlist-controlled, assessor-blinded trial will enroll 72 adults with PTSD and allocate them 1:1:1 to VR-based ETE, standard ETE, or a waitlist control group. Both active groups will receive 16 sessions over 8 weeks; waitlist participants will continue usual activities with scheduled monitoring. The primary endpoint is the between-group difference in the week-8 Korean version of the PTSD Checklist for DSM-5 (PCL-5-K) total score, adjusted for the baseline PCL-5-K score and study site. A week-12 follow-up assessment will examine the durability of treatment effects. Secondary outcomes, assessed at baseline and weeks 8 and 12, include the Core Seven-Emotions Inventory–Short Form, Korean Posttraumatic Growth Inventory, Five Facet Mindfulness Questionnaire–Short Form, Patient Health Questionnaire-15, Hospital Anxiety and Depression Scale, Korean Insomnia Severity Index, Hwa-Byung Scale, Clinical Global Impression scales, quantitative electroencephalography, and heart rate variability. Health-related quality of life and cost data will be collected for a within-trial economic evaluation.

**Discussion:**

This trial will provide preliminary randomized evidence on the efficacy, safety, and cost-effectiveness of standard and VR-based ETE for PTSD, and on whether adding VR to the meditation component of ETE offers clinical value beyond standard ETE. Interpretation will need to consider the modest sample size, the absence of participant blinding, and the use of a waitlist rather than an active control condition.

**Trial registration:**

Clinical Research Information Service of the Republic of Korea, KCT0007845. Registered on October 18, 2022.

## Introduction

Post-traumatic stress disorder (PTSD) is a chronic and potentially disabling psychiatric disorder that develops after exposure to actual or threatened death, serious injury, or sexual violence. It is characterized by intrusive memories, avoidance behaviors, negative alterations in cognition and mood, and hyperarousal, resulting in substantial impairment in daily functioning and health-related quality of life [1].

Trauma-focused psychotherapy is recommended as a first-line treatment for PTSD by major international clinical guidelines [2]. Established interventions include cognitive behavioral therapy, cognitive processing therapy, prolonged exposure therapy, narrative exposure therapy, eye movement desensitization and reprocessing, and stress inoculation training [2]. More recently, virtual reality (VR) has emerged as a promising platform for delivering psychological interventions in a standardized, immersive, and reproducible manner [3]. In patients with PTSD, VR enables controlled therapeutic environments while overcoming several practical limitations of real-world exposure-based interventions, thereby facilitating individualized treatment delivery [3]. Nevertheless, most existing VR-based interventions have been developed as virtual exposure therapies. To our knowledge, no randomized controlled trial has evaluated a traditional Korean medicine–based psychotherapy integrated with VR for PTSD, highlighting an important gap in the current literature.

Traditional Korean medicine adopts a holistic perspective in which mental and physical health are closely interconnected [4]. Within this framework, dysregulated emotional responses are considered important contributors to both psychological and physiological dysfunction [4]. Emotion-to-Emotion Therapy (ETE) is a structured psychotherapeutic approach derived from traditional Korean medicine that seeks to regulate excessive emotional responses by applying theoretically opposing emotions based on the Chiljeong (Seven Emotions) theory [4]. ETE has been incorporated into a Korean medicine–based treatment program for PTSD [5], and a standardized ETE treatment manual has been developed [6].

To our knowledge, no randomized controlled trial has evaluated the efficacy, safety, or cost-effectiveness of ETE for PTSD, either in its conventional format or when integrated with VR. Therefore, the present multicenter randomized controlled trial was designed to compare VR-based ETE, standard ETE, and a waitlist control in adults with PTSD. This protocol describes the study rationale, trial design, interventions, outcome measures, and statistical analysis plan for evaluating the efficacy, safety, and cost-effectiveness of VR-based ETE.

## Materials and methods

The original study protocol was prospectively registered with the Clinical Research Information Service of the Republic of Korea (CRIS; registration no. KCT0007845) on October 18, 2022, following Institutional Review Board (IRB) approval and before the initiation of participant recruitment. Subsequent approved amendments, including the multicenter expansion and protocol version 5.1, were approved by the relevant IRBs. The study protocol is presented in accordance with the SPIRIT guidelines [7,8] (S1 Checklist). The full study protocol is provided both in the version submitted to the IRB before recruitment began (version 2.0; S2 File, Korean; S3 File, English) and in the current IRB-approved version 5.1 (S4 File, Korean; S5 File, English).

### Study design

**Fig 1:**
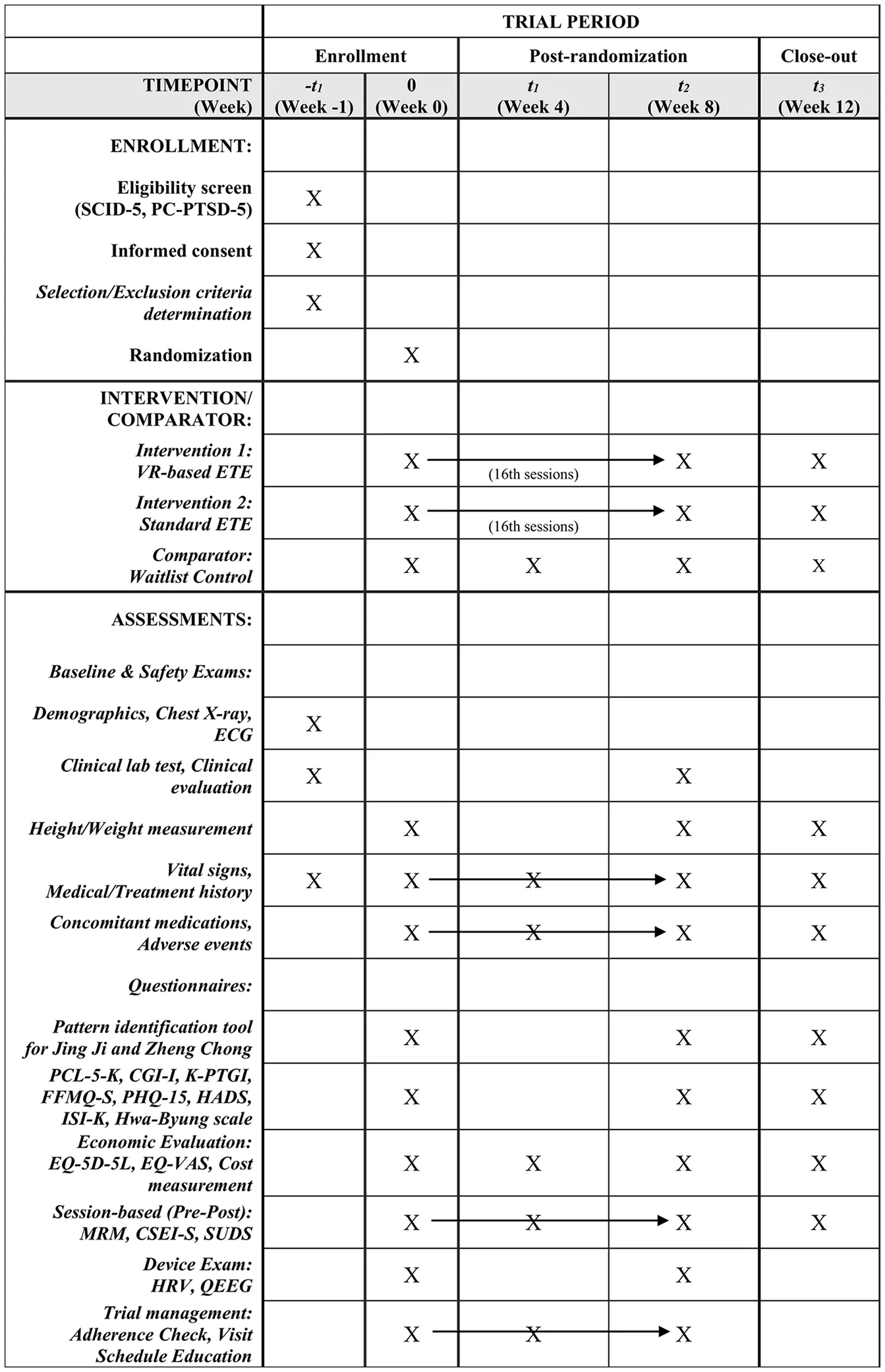
SPIRIT schedule of enrollment, interventions, and assessments. The screening period (−t_1_) is completed within 7 days before randomization. Mid-intervention assessment (t_1_) is performed at week 4, post-intervention assessment (t_2_) at week 8, and follow-up assessment (t_3_) at week 12. Participants in the two intervention groups attend two treatment sessions per week (16 sessions over 8 weeks). For the intervention groups, the week-4 assessment corresponds to the eighth treatment session, whereas for the waitlist control group it corresponds to the second study visit. Arrows indicate the continuous 16-session intervention period for the two intervention groups. Abbreviations: SCID-5, Structured Clinical Interview for DSM-5; PC-PTSD-5, Primary Care PTSD Screen for DSM-5; ECG, electrocardiogram; ETE, Emotion-to-Emotion Therapy; VR, virtual reality; PCL-5-K, Korean version of the PTSD Checklist for DSM-5; CGI-I, Clinical Global Impression–Improvement scale; K-PTGI, Korean version of the Posttraumatic Growth Inventory; FFMQ-S, Five Facet Mindfulness Questionnaire–Short Form; PHQ-15, Patient Health Questionnaire-15; HADS, Hospital Anxiety and Depression Scale; ISI-K, Korean version of the Insomnia Severity Index; EQ-5D-5L, EuroQol 5-Dimension 5-Level; EQ-VAS, EuroQol visual analogue scale; MRM, Mentalizing the Rooms of Mind; CSEI-S, Core Seven-Emotions Inventory–Short Form; SUDS, Subjective Units of Distress Scale; HRV, heart rate variability; QEEG, quantitative electroencephalography.

This study is a multicenter, three-arm, parallel-group, randomized, waitlist-controlled, assessor-blinded clinical trial. Seventy-two eligible participants will be randomly allocated in a 1:1:1 ratio to one of three groups: (1) VR-based Emotion-to-Emotion Therapy (VR-ETE), (2) standard Emotion-to-Emotion Therapy (ETE), or (3) a waitlist control group. The overall study design and assessment schedule are presented in Fig 1.

### Patient and public involvement

Patients and members of the public were not involved in the design of this trial, in the selection of the outcome measures, or in the development of the recruitment strategy, and no formal patient or public involvement is planned in the conduct or reporting of the trial.

### Participants

The trial was initially conducted at Wonkwang University Sanbon Hospital as a single-center study. IRB approval was obtained on October 11, 2022 (WMCSB 202209−83). The study was prospectively registered with the CRIS (KCT0007845) on October 18, 2022, and participant recruitment commenced on November 18, 2022. Accordingly, participant recruitment began only after both ethical approval and prospective trial registration had been completed. Participants are recruited through local newspaper and online advertisements, hospital bulletin boards, and public notices at the participating sites.

#### Inclusion criteria.

1. Adults aged 18–70 years (inclusive), both males and females.
2. Individuals meeting the Diagnostic and Statistical Manual of Mental Disorders, Fifth Edition (DSM-5) diagnostic criteria for PTSD as confirmed by the Structured Clinical Interview for DSM-5 (SCID-5).
3. Individuals scoring 3 or more on the Primary Care PTSD Screen for DSM-5 (PC-PTSD-5). This cutoff is supported by the Korean validation study, which identified a score of 3 as the threshold for clinically significant PTSD symptoms [9]. In this protocol, the PC-PTSD-5 is used as a screening tool to identify individuals warranting full diagnostic evaluation and is followed by a confirmatory SCID-5 interview [10].
4. Individuals who voluntarily decide to participate and sign the informed consent form.

#### Exclusion criteria.

1. Individuals with a severe psychiatric disorder, defined as (i) a current or past history of delusions or hallucinations; (ii) at least one previous manic episode; (iii) a history of alcohol abuse or dependence; or (iv) an assessed risk of suicide. Criterion (i) is applied because the emotionally evocative nature of VR-assisted meditation may increase the risk of symptom exacerbation in individuals with psychotic vulnerability. The potential implications of this criterion for external validity are addressed in the Discussion.
2. Individuals who require continuous administration of substances (other than the permitted concomitant medications) that are believed to influence the induction of symptoms.
3. Individuals with congenital diseases or severe issues with the central and peripheral nervous systems, endocrine system, immune system, heart, liver, or kidneys.
4. Pregnant or breastfeeding individuals.
5. Individuals who have participated in another clinical study in the past month or are currently participating in another clinical study.
6. Individuals deemed unsuitable for participation by the clinical trial investigator or the responsible investigator.

#### Participant withdrawal criteria.

Participants are withdrawn for (1) non-compliance with investigator instructions, (2) protocol violations, (3) occurrence of serious adverse events, or (4) withdrawal of consent or a request to discontinue. Adherence is defined as (number of sessions actually completed ÷ number of sessions scheduled) × 100, recorded at every treatment visit. Participants with adherence below 75% will be excluded from the per-protocol (PP) analysis but will remain in the intention-to-treat (ITT) analysis. Participants who discontinue the assigned intervention but do not withdraw consent for follow-up will be encouraged to complete all scheduled outcome assessments whenever possible.

### Randomization, allocation concealment, and blinding

Sequence generation. The randomization list is generated by a randomization officer or statistician not involved in trial conduct or evaluation, using StataMP 16 (StataCorp LLC, College Station, TX, USA) block randomization with a 1:1:1 allocation ratio. A single allocation sequence is used for the trial as a whole; no separate sequence was created when the second site joined, and randomization is therefore not stratified by study site. Participants are allocated from this sequence in the order in which they are enrolled, irrespective of the site at which they present (competitive enrollment). The block size was set by the independent randomization officer. Because recruitment is ongoing, the block size is not reported in this protocol paper, in order to preserve allocation concealment and to prevent prediction of upcoming allocations; it will be reported in full in the primary results publication. The allocation sequence remains inaccessible to the investigators responsible for participant enrollment and outcome assessment throughout the trial. Study site is nevertheless included as a covariate in all prespecified efficacy models.

Allocation concealment. The randomization sequence is concealed using sequentially numbered, opaque, sealed envelopes, opened in order by the study investigator in front of each participant; the date of opening and the investigator’s signature are recorded on each envelope, which is then stored separately.

Blinding. Outcome assessment is conducted by assessor-blinded outcome collectors—independent clinical psychologists who are not involved in delivering the interventions and who remain unaware of group allocation during data collection. Because participants cannot be blinded to their own treatment allocation in a trial of this nature, and because the outcome battery is predominantly self-reported, assessor blinding provides only limited protection against participant performance and reporting bias; the assessor-rated Clinical Global Impression scales and the device-based quantitative electroencephalography (QEEG) and heart rate variability (HRV) measures offer partial, though not complete, protection against this source of bias. This limitation is discussed further below.

### Sample size

This study is designed as an exploratory, hypothesis-generating randomized controlled trial. G*Power 3.1.9.4 indicated that, assuming a large effect size (f = 0.5), 95% power, a two-sided alpha of 0.05, three groups, and one covariate in an analysis of covariance (ANCOVA) framework, 65 participants would be required for the primary omnibus group comparison. The target sample was set at 72 participants (24 per group), balancing the conventional omnibus calculation with feasible recruitment, available resources, and the planned study period. The sample-size calculation addresses the overall three-group effect and was not intended to provide definitive power for individual pairwise comparisons, particularly VR-based ETE versus standard ETE. Accordingly, the comparison between the two active interventions will be interpreted as exploratory, with emphasis on effect estimates and 95% confidence intervals rather than a definitive superiority or non-inferiority conclusion. Attrition may reduce precision; therefore, the findings will primarily inform effect-size estimation and sample-size planning for a future confirmatory trial.

### Interventions

The interventions are reported in accordance with the Template for Intervention Description and Replication (TIDieR) checklist [11] (S6 File). The intervention consists of three sequential phases: (1) a screening phase, (2) an 8-week treatment phase, and (3) a follow-up assessment at week 12. Participants allocated to the VR-ETE and standard ETE groups receive 16 treatment sessions over 8 weeks (two sessions per week, 40 minutes per session). Both active interventions are delivered individually, one clinician to one participant, face-to-face in an outpatient consultation room at each study site; no group sessions and no remote or unsupervised home sessions are used. Participants allocated to the waitlist control group continue their usual daily activities for 8 weeks while receiving fortnightly telephone monitoring. They attend the same assessment visits as the intervention groups at baseline, week 4, week 8, and week 12 but do not receive the study intervention during the randomized phase.

#### ETE procedure.

ETE comprises five stages (total 40 minutes): (1) assessment using the Core Seven-Emotions Inventory–Short Form (CSEI-S), Mentalizing the Rooms of Mind (MRM), and the Subjective Units of Distress Scale (SUDS; 5 min); (2) analysis, comprising identification of the participant’s predominant emotional pattern based on traditional Korean medicine principles (Yin-Yang and Five-Elements theory), followed by selection of the appropriate ETE intervention module (5 min); (3) ETE training, comprising emotion induction, breathing awareness, body-awareness training, upper-, middle-, and lower-Dan-Tien meditation, Xi-Xi meditation, resource meditation, and integrated three-Dan-Tien meditation (20 min); (4) reassessment using the CSEI-S, MRM, and SUDS (5 min); and (5) emotional-regulation education (5 min). For participants with PTSD, the principal intervention module is Si-Seung-Gong (思勝恐), a traditional Korean medicine emotion-regulation technique based on the principle that cognitive awareness (“thought”) is used to regulate excessive fear. MRM is a projective drawing-based clinical technique used in M&L psychotherapy in which participants visualize and depict their current internal state as a set of rooms representing thoughts, emotions, memories, sources of distress, and psychological resources. The technique is intended to help participants objectify and reflect on their internal experiences and to support clinical assessment and session-by-session monitoring of psychological change [12].

#### VR-based ETE procedure.

The VR intervention is delivered using a Meta Oculus Quest 2 head-mounted display (Meta Platforms Inc., Menlo Park, CA, USA) connected to a desktop computer running a dedicated VR-ETE software application (Version 1.0; ZOIGRAM, Seoul, Republic of Korea). The approximately 20-minute training phase delivers the same therapeutic meditation modules used in standard ETE, including emotional trigger, breathing, body-sensation, upper Dan-Tien meditation, lower Dan-Tien meditation, middle Dan-Tien meditation, Xi-Xi, resource, and integrated three-Dan-Tien meditation. These modules are delivered as narrated, scripted, audio-guided sequences within an immersive virtual environment. For participants with PTSD, upper Dan-Tien meditation (Si-Seung-Gong) is used as the primary module. Treatment adaptation is clinician-directed rather than software-driven. Before each intervention session, the treating clinician selects the appropriate VR meditation module or modules based on the participant’s pre-session CSEI-S and SUDS scores and the clinical findings derived from MRM. The VR software does not provide automated or real-time algorithmic adaptation. No separate pilot or usability study was conducted for this specific VR-ETE application before the trial; however, the therapeutic content incorporated into the application was based on the previously developed standardized ETE manual [6]. All other procedural stages—including assessment, analysis, reassessment, and education—are identical to those used in standard ETE.

#### Control group.

The waitlist control group receives no ETE intervention during the 8-week randomized intervention period but receives telephone check-ins every 2 weeks. Waitlist participants complete the week-12 follow-up assessment at the same time as participants in the active intervention groups. No booster or maintenance intervention is provided to any group between weeks 8 and 12; this interval is used solely for follow-up monitoring and assessment. No waitlist participant will begin ETE or VR-based ETE before the week-12 follow-up assessment has been fully completed. Only after completion of this final randomized-phase assessment may waitlist participants elect to receive 8 weeks of VR-based ETE, comprising 16 sessions, free of charge. The per-visit transportation stipend provided during the randomized phase is not provided for this post-trial intervention.

#### Intervention standardization and treatment fidelity.

As specified in the approved protocol, the interventions are delivered by board-certified Korean medicine neuropsychiatrists, or by licensed Korean medicine doctors with at least 1 year of clinical experience working under the direct supervision of a board-certified Korean medicine neuropsychiatrist. In practice, all interventions in this trial were delivered by board-certified Korean medicine neuropsychiatrists. To minimize inter-center variability, clinicians at both sites deliver the two active interventions using the same standardized ETE manual, session sequence, module-selection criteria, treatment schedule, and structured case-report forms. Treatment fidelity is monitored through completion of the structured case-report forms, and protocol deviations are documented. Study site is included as a covariate in the efficacy analyses. No independent rating of session recordings or formal inter-rater fidelity assessment is performed.

#### Concomitant treatments and medication management.

Participants receiving counseling for pre-existing PTSD may continue counseling if the treatment regimen has remained stable for at least 3 months before screening. Participants taking hypnotics, antidepressants, antipsychotics, or anxiolytics may continue these medications if the dosage has remained stable for at least 4 weeks before screening and remains unchanged during the trial. Medications for unrelated pre-existing conditions may be continued without restriction, including antihypertensive, antidiabetic, lipid-lowering, and analgesic medications. Medication use, including drug class and dosage stability, is recorded at every scheduled visit and summarized descriptively by treatment group. Any unplanned medication change during the trial is documented as a protocol deviation and evaluated in a sensitivity analysis rather than resulting in automatic exclusion, unless the change meets a prespecified withdrawal criterion. No medication washout period is required. Accordingly, the study follows a pragmatic trial design with respect to concomitant psychiatric medication use.

### Baseline assessments

At baseline, the Gyeonggye-Jeongchung pattern identification tool (驚悸怚忡; “fright palpitations,” also transliterated as Jing Ji and Zheng Chong) [13] is administered to characterize a traditional Korean medicine phenotype related to hyperarousal and anxiety-associated palpitation symptoms, which are clinically relevant to PTSD. The resulting pattern classification is intended to support exploratory hypothesis generation regarding whether baseline traditional Korean medicine pattern characteristics identify participants with differential treatment responses. Pattern classifications will be summarized descriptively by treatment group and, if subgroup sizes permit, may be examined as exploratory predictors or effect modifiers using subgroup summaries or treatment-by-pattern interaction terms. These analyses are not powered for definitive subgroup inference, and the pattern classification will not be included as a covariate in the primary confirmatory analysis.

### Outcome measures

The primary outcome measure is the Korean version of the PTSD Checklist for DSM-5 (PCL-5-K), a 20-item self-report measure of PTSD symptom severity with a total score ranging from 0 to 80. The PCL-5-K is administered at baseline, week 8, and week 12, with week 8 designated as the primary endpoint [14].

Secondary outcome measures include the Clinical Global Impression–Severity Scale (CGI-S) and Clinical Global Impression–Improvement Scale (CGI-I) [15], the Korean version of the Posttraumatic Growth Inventory (K-PTGI) [16], the Five Facet Mindfulness Questionnaire–Short Form (FFMQ-S) [17], the Patient Health Questionnaire-15 (PHQ-15) [18], the Hospital Anxiety and Depression Scale (HADS) [19], the Korean version of the Insomnia Severity Index (ISI-K) [20], and the Hwa-Byung Scale [21]. Hwa-Byung (火病, literally “fire illness”) is a Korean culture-bound syndrome characterized by suppressed anger with combined psychological and somatic manifestations; the Hwa-Byung Scale is a 31-item self-report instrument comprising a 15-item symptom subscale and a 16-item personality subscale, each item rated on a 5-point scale (0–4), with higher scores indicating greater severity [21]. Secondary outcome measures are administered at baseline, week 8, and week 12, as applicable. The CGI-S is administered at baseline to characterize overall illness severity. The CGI-I is administered only at weeks 8 and 12 to evaluate improvement relative to the pretreatment condition; no CGI-I score is assigned at baseline.

The CSEI-S and the SUDS are administered immediately before and after each intervention session. The CSEI-S is used to assess changes in core emotional states [22]. The SUDS, originally introduced by Wolpe and Lazarus, is a brief self-report rating of momentary subjective distress and is used to support session-level clinical monitoring [23]. Because it is used here for within-session clinical monitoring rather than as a validated confirmatory endpoint, the SUDS will be treated as an exploratory session-level measure. MRM is administered at the same time points as a projective drawing-based clinical technique. Participants visualize and depict their current internal experiences as rooms representing thoughts, emotions, memories, distress, and psychological resources. MRM is used to support clinical assessment, self-reflection, treatment-module selection, and qualitative monitoring of psychological change [12]. It is not treated as a standardized continuous efficacy outcome.

QEEG is assessed at baseline and week 8. Resting-state electroencephalography is recorded using a 21-channel Neuron-Spectrum-4/P system (Neurosoft, Ivanovo, Russia) with an electrode cap containing 21 surface electrodes positioned according to the International 10–20 System. Five-minute eyes-closed and five-minute eyes-open recordings are obtained. Electrode impedance is maintained below 5 kΩ at all sites. Linked ears (A1/A2) are used as the reference, and the ground electrode is positioned midway between Fz and Cz. Artifacts, including eye blinks, ocular movements, and muscle activity, are screened and excluded. Data quality is subsequently verified in NeuroGuide version 3.4.0 (Applied Neuroscience, Inc., Largo, FL, USA), and only recordings with test–retest reliability coefficients of ≥ 0.95 are retained for analysis. The prespecified QEEG indices include absolute power, relative power, z-scores, and interelectrode coherence across the delta, theta, alpha, beta, and gamma frequency bands. The approved protocol additionally lists source localization using low-resolution brain electromagnetic tomography (LORETA) among the exploratory electroencephalographic analyses. This analysis is not included in the prespecified analysis plan for the present trial: source estimation from a 21-channel montage has limited spatial resolution, and the available sample size does not support inference at the level of individual Brodmann areas once the multiplicity correction described below is applied. The prespecified QEEG analyses are therefore restricted to the sensor-level indices listed above.

HRV is assessed at baseline and week 8 using an SA-3000P analyzer (Medicore Co., Ltd., Seoul, Republic of Korea). Participants are seated comfortably in a chair with back support and rest for 5 minutes before a 3-minute recording. Breathing is spontaneous and is not paced or otherwise controlled. The manufacturer-provided software automatically calculates time-domain indices (standard deviation of NN intervals [SDNN] and root mean square of successive differences [RMSSD]) and frequency-domain indices (low frequency [LF], high frequency [HF], and LF/HF ratio), and the generated values are used for analysis.

### Economic evaluation

The economic evaluation is designed as a 12-week within-trial analysis. The EuroQol 5-Dimension 5-Level instrument (EQ-5D-5L) and the EuroQol visual analogue scale (EQ-VAS) are administered at baseline and at weeks 4, 8, and 12 [24]. EQ-5D-5L utility scores will be derived using the Korean EQ-5D-5L value set and used to calculate quality-adjusted life-years (QALYs) using the area-under-the-curve method, with linear interpolation between assessment points. Cost data are collected at the same assessment time points using a purpose-built cost questionnaire covering direct medical costs, direct non-medical costs, and indirect costs attributable to productivity loss. In-trial intervention costs are calculated by multiplying the recorded number of sessions by the corresponding prespecified unit costs. After unblinding, researchers obtain costs incurred at the study sites from the participating sites’ billing and electronic records. All resource-use costs will be valued in 2026 Korean won. Where cost inputs originate from earlier calendar years, they will be converted to the 2026 price year using the Korean Consumer Price Index. Because the analytic time horizon is 12 weeks and therefore less than 1 year, neither costs nor health outcomes will be discounted. The economic evaluation will follow the relevant recommendations of the Korean pharmacoeconomic evaluation guideline [25] and the CHEERS 2022 reporting standards [26]. Productivity loss is valued using the human-capital approach because the cost questionnaire does not collect the return-to-work timing data required to apply the friction-cost method.

### Safety monitoring

All adverse events (AEs), regardless of their relationship to the intervention, are recorded and classified according to severity as mild, moderate, or severe. Causality is rated as definitely related, probably related, possibly related, probably not related, not related, or unknown. Anticipated device-related AEs are expected to be mild and transient and may include eyestrain, dizziness, and mild physical discomfort associated with routine head-mounted display use. No independent Data and Safety Monitoring Board (DSMB) was established because the trial was considered to pose minimal risk. The underlying ETE therapeutic content is already used in routine Korean medicine practice, and the additional technological component consists of a brief, non-invasive head-mounted display-based training segment. All serious adverse events (SAEs) are reported by the investigator to the principal investigator and the IRB within 24 hours, regardless of causality. The principal investigator may suspend part or all of the trial pending further review or instruction from the IRB. No interim efficacy analysis or formal statistical stopping rule is prespecified. Safety oversight is based on continuous AE monitoring, expedited SAE reporting, and review by the principal investigator and the IRB.

### Statistical analysis

Clinical and economic analyses will be conducted using R software (version 4.3.2; R Foundation for Statistical Computing, Vienna, Austria) and StataMP version 16 (StataCorp LLC, College Station, TX, USA). QEEG and HRV indices will be extracted using NeuroGuide version 3.4.0 and the SA-3000P software, respectively. The exact software and package versions used will be reported after completion of the final analysis.

#### Analysis populations.

The primary efficacy analysis will follow the ITT principle and include all randomized participants according to their originally assigned treatment group, regardless of treatment adherence, withdrawal from treatment, protocol deviations, or the study site at which they were enrolled. Accordingly, all participants randomized at Wonkwang University Sanbon Hospital before recruitment at that site ended will remain in the ITT analysis and will not be excluded because of the site closure. Any imbalance between sites will be accounted for by including study site as a covariate in the prespecified efficacy models. Missing week-8 outcome data will be addressed using multiple imputation. A PP population will be used for sensitivity analyses and will exclude participants who complete less than 75% of the prescribed intervention sessions or who have major protocol deviations considered likely to affect efficacy assessment. Baseline demographic and clinical characteristics, including age, sex, study site, baseline PCL-5-K score, and the index trauma type recorded during the SCID-5 interview, will be summarized descriptively by treatment group. No significance testing of baseline balance will be performed; any imbalance will be described and considered in the interpretation of the results.

#### Primary confirmatory analysis.

The primary confirmatory analysis will assess the overall treatment-group effect on the week-8 PCL-5-K total score using ANCOVA. The week-8 PCL-5-K total score will be entered as the dependent variable, with treatment group as a fixed effect and baseline PCL-5-K score and study site as covariates. The overall treatment-group effect will be evaluated using a two-sided significance level of 0.05. If the overall treatment-group effect is statistically significant, the prespecified comparisons of VR-based ETE versus waitlist control and standard ETE versus waitlist control will be conducted using a Dunnett adjustment for multiple comparisons. Adjusted mean differences, 95% confidence intervals, and corresponding p values will be reported. The comparison between VR-based ETE and standard ETE will be treated as exploratory because the trial was not powered to establish superiority or non-inferiority between the two active interventions.

#### Secondary and exploratory analyses.

Continuous secondary outcomes measured at week 8—the K-PTGI, FFMQ-S, PHQ-15, HADS, ISI-K, and Hwa-Byung Scale total scores—will each be analyzed using a separate ANCOVA, with the week-8 score as the dependent variable and the corresponding baseline score, treatment group, and study site included in the model. These analyses will be considered secondary or exploratory, and adjusted mean differences with 95% confidence intervals will be reported. QEEG parameters (absolute power, relative power, z-scores, and interelectrode coherence across the delta, theta, alpha, beta, and gamma frequency bands) and HRV parameters (SDNN, RMSSD, LF, HF, and LF/HF) measured at week 8 will be analyzed using the same ANCOVA framework, with the corresponding baseline value and study site included as covariates. Each physiological parameter will be modeled separately rather than entering multiple highly correlated QEEG or HRV indices simultaneously as predictors. To control the false discovery rate across the complete physiological outcome family, the Benjamini–Hochberg procedure will be applied jointly to the full set of prespecified QEEG and HRV hypothesis tests, including all electrodes, frequency bands, coherence pairs, and HRV indices. Both unadjusted and adjusted p values will be reported, and statistical interpretation will be based primarily on the adjusted values, effect sizes, and 95% confidence intervals. These high-dimensional physiological analyses will be considered exploratory.

The persistence of treatment effects through week 12 will be examined using a mixed-effects model for repeated measures including the post-baseline observations at weeks 8 and 12. The model will include treatment group, assessment time, the treatment-group-by-time interaction, the corresponding baseline outcome score, and study site. Within-participant correlations will be accounted for using an appropriate repeated-measures covariance structure. Adjusted between-group differences at weeks 8 and 12 will be reported. This model will be applied to the primary outcome (PCL-5-K) and, as exploratory analyses, to the K-PTGI, FFMQ-S, PHQ-15, HADS, ISI-K, and Hwa-Byung Scale total scores.

Baseline CGI-S scores will be summarized descriptively by treatment group. CGI-I scores obtained at weeks 8 and 12 will be analyzed as ordinal outcomes using an ordinal regression model. If the proportional-odds assumption is not satisfied, an appropriate alternative ordinal or nonparametric method will be used. Session-level CSEI-S and SUDS scores will be summarized by treatment group and session. Pre-session to post-session changes in CSEI-S and SUDS scores may be examined using mixed-effects models as exploratory analyses. MRM drawings will be summarized descriptively and qualitatively in accordance with a prespecified coding framework informed by the existing MRM literature. MRM will not be included as a continuous outcome in inferential statistical models unless a prespecified quantitative coding and scoring procedure is applied.

#### Missing data.

Missing week-8 efficacy data will be addressed using multiple imputation under a missing-at-random assumption. The imputation model will include randomized treatment group, baseline and available post-baseline outcome measurements, and study site. The primary ANCOVA will be fitted to each imputed dataset, and estimates will be combined using Rubin’s rules. Sensitivity analyses will include a mixed-effects model using all available longitudinal observations and, where appropriate, complete-case analysis. The PP analysis will provide an additional supportive sensitivity analysis. Except for multiplicity-adjusted comparisons, statistical tests will be two-sided with a significance level of 0.05.

#### Nonparametric and assumption-robust procedures.

The distributional assumptions of the planned parametric models will be evaluated using residual diagnostics and descriptive assessments of the outcome distributions. When the assumptions of parametric analyses are not adequately satisfied, appropriate transformations, robust standard errors, or nonparametric procedures will be considered. Exploratory within-group pre–post comparisons may be conducted using the Wilcoxon signed-rank test. Between-group comparisons may be conducted using the Kruskal–Wallis test, followed, where appropriate, by pairwise Mann–Whitney U tests with a Bonferroni-type adjustment. These within-group and nonparametric analyses will be considered supportive or exploratory and will not replace the prespecified primary ANCOVA.

#### Economic analysis.

The primary economic outcome will be the incremental cost per QALY gained. QALYs will be calculated from EQ-5D utility scores using the area-under-the-curve method, with linear interpolation between assessment points. The secondary economic outcome will be the incremental cost per unit improvement in EQ-VAS score. The two primary economic comparisons will mirror the confirmatory clinical comparisons: VR-based ETE versus the waitlist control condition and standard ETE versus the waitlist control condition. The comparison between VR-based ETE and standard ETE will be reported as an exploratory secondary economic comparison. The base-case within-trial economic evaluation will cover the 8-week intervention period and the subsequent assessment-only follow-up through week 12. Costs and health outcomes will be analyzed using the same ITT population as the primary clinical analysis. The PP population will be examined in sensitivity analyses. For each comparison, mean costs, mean QALYs, incremental costs, incremental QALYs, and incremental cost-effectiveness ratios will be estimated. Sampling uncertainty surrounding costs, QALYs, and incremental cost-effectiveness ratios will be examined using nonparametric bootstrapping. Cost-effectiveness planes and cost-effectiveness acceptability curves will be presented where appropriate. Because the 12-week horizon is insufficient to represent the long-term course of PTSD, the analysis is not intended to establish long-term or lifetime cost-effectiveness. Rather, it will generate preliminary short-term estimates of costs, utilities, QALYs, and uncertainty that may serve as candidate input parameters for future decision-analytic modeling, including a longer-term Markov model if appropriate external transition and recurrence data become available.

Missing cost, EQ-5D-5L, and EQ-VAS data in the within-trial analysis will be addressed using multiple imputation under a missing-at-random assumption. Because cost data are commonly right-skewed, appropriate generalized linear models or bootstrap-based methods will be used when estimating between-group differences in mean costs.

### Data management and quality control

Investigators collect and record information for each participant on a structured case report form. Data are kept confidential in accordance with the privacy policies of the participating institutions and are stored in password-protected computers or double-locked cabinets accessible only to authorized researchers. All original source documents, including questionnaires, medical records, and related materials, are retained for the period required by applicable Korean regulations and are then destroyed. The research team is monitored internally to verify the protection of participant rights, adherence to the protocol, the quality of data collection and handling, and the accuracy of recruitment records. All clinicians and researchers involved in the trial have completed Good Clinical Practice training. Data will not be shared while the trial is ongoing. After completion of the study and publication of the results, the de-identified minimal dataset underlying the reported findings, together with a data dictionary and the analysis code, will be deposited in Figshare (https://figshare.com) and a persistent identifier will be provided, subject to applicable participant-consent, ethical, privacy, and legal restrictions.

### Ethical considerations

The study was initially approved by the IRB of Wonkwang University Sanbon Hospital on October 11, 2022 (WMCSB 202209−83). It was subsequently approved by the IRB of Wonkwang University Korean Medicine Hospital on November 7, 2023 (WKUIOMH-IRB-2023-11), before recruitment commenced at the second study site. The study was registered with the CRIS on October 18, 2022 (KCT0007845). Registration occurred after approval by the IRB of the initial study site on October 11, 2022, and before recruitment of the first participant began on November 18, 2022. The second study site joined the multicenter trial only after obtaining approval from its own IRB, and each protocol amendment was implemented only after approval by the relevant IRB or IRBs. Written informed consent is obtained by the investigator at the screening visit, before any study-specific procedure is performed and before a screening number is assigned. The investigator explains the purpose and procedures of the trial, the foreseeable risks and benefits, the voluntary nature of participation, and the right to withdraw at any time without penalty, and provides an opportunity to ask questions before the consent form is signed; a copy of the signed form is given to the participant. The study is conducted in accordance with the Declaration of Helsinki and applicable institutional and national ethical requirements. Provisions for compensation in the event of trial-related harm are set out in the participant information and consent documents approved by the Institutional Review Board. Post-trial care is limited to the offer of VR-based ETE to waitlist participants after completion of the week-12 assessment, as described above.

The trial began as a single-center study at Wonkwang University Sanbon Hospital, where recruitment commenced on November 18, 2022. Following the relocation of the principal investigator, the study was expanded into a multicenter trial through the addition of Wonkwang University Korean Medicine Hospital. The second site obtained ethical approval on November 7, 2023, before any participant was recruited there. Early termination of recruitment at Wonkwang University Sanbon Hospital was approved on August 26, 2024 for administrative reasons. Recruitment is continuing at Wonkwang University Korean Medicine Hospital. All participants randomized at the Sanbon site before recruitment at that site ended will remain in the ITT population and will be analyzed according to their original randomized allocation; no participant will be excluded solely because recruitment at that site ended. Potential site imbalance will be accounted for through the prespecified inclusion of study site in the statistical models. Protocol version 5.1, dated November 19, 2025, is the current IRB-approved amended protocol and incorporates these institutional transitions and the associated modifications to study procedures. Recruitment is expected to be completed by September 2026, data collection by December 2026, and the final statistical and economic analyses and reporting by April 2027.

## Discussion

ETE is a Korean medicine-based psychotherapeutic approach that integrates emotion-focused assessment and regulation with meditation-based training. Existing publications describe the theoretical basis, manual development, and preliminary clinical application of ETE; however, robust comparative evidence of efficacy for PTSD remains limited [4–6]. Engagement with meditation training may also vary across participants. The VR component was therefore introduced to provide a more standardized and immersive delivery environment. Evidence regarding VR-based mindfulness and meditation is emerging; however, the available studies remain heterogeneous and do not establish that immersive VR necessarily produces superior clinical outcomes compared with conventional delivery [27].

This exploratory, hypothesis-generating, multicenter, randomized, assessor-blinded, waitlist-controlled trial was designed to evaluate the efficacy, safety, and economic outcomes of VR-based ETE and standard ETE for PTSD. The week-12 assessment, conducted 4 weeks after completion of the 8-week intervention, will provide preliminary information on persistence of treatment effects. Waitlist participants remain without ETE intervention until the week-12 assessment has been completed, thereby preserving week-12 data as follow-up under the randomized waitlist condition before optional post-trial treatment. The waitlist condition provides a comparator for symptom change over time and repeated assessment; however, it does not control for therapist attention, treatment expectancy, or other nonspecific intervention effects and may yield larger effect estimates than an active control [28]. The study also includes device-based physiological outcomes, including QEEG and HRV, which may provide exploratory information on neurophysiological and autonomic changes.

To promote standardized delivery across sites, the same clinician eligibility criteria, ETE manual, session structure, module-selection procedures, treatment schedule, and structured case-report forms are used. Study site is included in the primary and secondary efficacy models. Nevertheless, because fidelity is monitored through structured documentation rather than independent ratings of session recordings, complete equivalence of intervention delivery across clinicians and sites cannot be established.

The study aims to provide foundational comparative evidence regarding conventional and VR-based ETE as potential treatment strategies for PTSD. It will also inform the feasibility of integrating immersive digital technology into traditional Korean medicine-based psychotherapy.

This study has several limitations. First, double-blinding is not feasible, and assessor blinding cannot prevent expectancy and reporting bias in predominantly self-reported outcomes. Second, no independent DSMB was established and no formal interim efficacy stopping rule was prespecified; safety oversight relies on continuous adverse-event reporting and review by the principal investigator and the IRB. Third, randomization was not stratified by study site, trauma type, or baseline severity. Study site and baseline severity are included as covariates in the prespecified models, but covariate adjustment can improve precision without fully removing imbalance in unmeasured prognostic factors. Index trauma type is ascertained during the SCID-5 interview and will be summarized descriptively by treatment group, but it is not included as a covariate because the anticipated number of participants in each trauma category is too small to support adjusted inference. Fourth, as an exploratory trial, the sample size supports preliminary estimation of the overall treatment-group effect but not definitive pairwise inference, particularly for VR-based ETE versus standard ETE; the large assumed effect size and potential attrition may further limit precision. Fifth, exclusion of participants with a history of psychosis may limit generalizability. Sixth, although shared manualized procedures and structured case-report forms are used across sites, fidelity is not assessed through independent recording-based ratings, and no formal inter-rater fidelity assessment is performed. Seventh, no separate pilot or usability study of the specific VR-ETE application was completed before the trial. Eighth, the 12-week within-trial economic horizon cannot establish the long-term cost-effectiveness of either intervention; it can only provide preliminary estimates and candidate parameters for future decision-analytic modeling.

Within these constraints, the protocol is expected to provide useful preliminary evidence on the clinical and economic outcomes of VR-integrated Korean medicine psychotherapy for PTSD and to identify priorities for a larger, independently fidelity-monitored trial using an active control.

## Dissemination

The study findings will be disseminated through peer-reviewed publications and presentations at academic conferences and international scientific meetings.

## Supporting information

S1 Checklist SPIRIT 2025 checklist for the study protocol. (DOCX)

S2 File Study protocol submitted to the Institutional Review Board, version 2.0 (Korean; original pre-recruitment version). (PDF)

S3 File Study protocol submitted to the Institutional Review Board, version 2.0 (English; original pre-recruitment version). (DOCX)

S4 File Institutional Review Board-approved study protocol, version 5.1 (Korean; current amended version). (DOCX)

S5 File Institutional Review Board-approved study protocol, version 5.1 (English; current amended version). (DOCX)

S6 File Completed TIDieR checklist for the interventions. (DOCX)
