# Supplementary material for: Virtual reality-based emotion-to-emotion therapy for post-traumatic stress disorder: Protocol for a randomized controlled trial of efficacy, safety, and cost-effectiveness
Source: PLoS One. 2026 Sep 2;21(9):e0357593. doi: 10.1371/journal.pone.0357593 (PMC13537590; doi:10.1371/journal.pone.0357593)
Supplement: Supplementary file 1 [file pone.0357593.s002.pdf]

PROTOCOL

- 임상시험계획서 -

외상후 스트레스 장애 환자에 대한 가상현실 기반 오지상승위치료법의 안전성, 유효성 및 경제성 평가 임상시험

Clinical trial to evaluate the safety, efficacy, and economic feasibility of virtual reality-based 'Emotion to Emotion Therapy' for post-traumatic stress disorder patients

Protocol No.	**WS_NP_2022_01**
Protocol Ver.	Ver. 2.0
임상시험 실시기관	원광대학교 산본병원
시험책임자	강 형 원
발행 일자	2022.09.25.

CONFIDENTIAL

본 임상시험계획서에 포함된 모든 정보는 임상시험 관련자, 기관생명윤리위원회, 보건당국을 위해 제공된 것으로 임상시험용 의약품을 투여받을 사람에게 시험참가에 대한 서면 동의를 받기 위한 경우를 제외하고는 임상시험책임자의 사전 서면 동의 없이 제3자에게 공개될 수 없습니다.

▣ 임상시험계획서 제개정 이력

버전 번호	일자	주요 내용
Ver. 1.0	2022.07.28.	임상시험계획서 제정
Ver. 2.0	2022.09.25.	경제성평가관련 내용 추가 및 CRF 수정

Protocol Agreement

외상후 스트레스 장애 환자에 대한 가상현실 기반 오지상승위치료법의 안전성, 유효성 및 경제성 평가 임상시험

Clinical trial to evaluate the safety, efficacy, and economic feasibility of virtual reality-based 'Emotion to Emotion Therapy' for post-traumatic stress disorder patients

임상시험계획서번호: WS_NP_2022_01
계획서 버전: 2.0
최종 작성일: 2022.09.25.

- 본인은 임상시험계획서를 숙지하였으며, 모든 규정과 지침서에 따라 본 시험을 수행할 것을 동의합니다.
- 본인은 이 임상시험계획서와 관련하여 개발하거나 얻은 모든 정보의 보안을 유지할 것을 동의합니다.

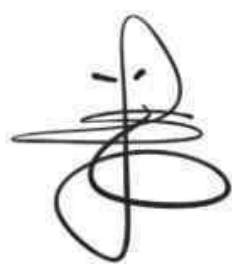

Principal Investigator
원광대학교 산본병원 강형원

2022.09.25.
Date

목 차

▣ 임상연구계획서 개요 ······ 1
1. 임상시험의 명칭 및 단계 ······ 7
1.1 명칭 ······ 7
1.2 단계 ······ 7
2. 임상시험 실시기관, 임상시험책임자/담당자 및 의뢰자 ······ 7
2.1 주관기관 및 주소 ······ 7
2.2 실시기관 및 주소 ······ 7
2.3 임상시험 의뢰자 및 주소 ······ 7
3. 임상시험의 목적 및 배경 ······ 7
3.1 임상시험의 목적 ······ 7
3.2 임상시험의 배경 ······ 7
3.3 연구 내용, 범위 및 방법 ······ 7
3.4 연구결과의 기대효과 및 활용 ······ 9
4. 대상 질환 및 임상시험 기간 ······ 10
4.1 대상 질환 ······ 10
4.2 임상시험 기간 ······ 10
5. 피험자의 선정기준, 제외기준 및 목표 피험자의 수와 선정근거 ······ 10
5.1 선정기준 ······ 10
5.2 제외기준 ······ 10
5.3 병용약물 ······ 10
5.4 목표한 피험자의 수 및 산출근거 ······ 11
6. 임상시험 방법 ······ 12
6.1 임상시험의 설계 ······ 12
6.2 임상시험 설계 요약 ······ 12
6.3 PTSD환자 대상 오지상승위치료법과 VR 기반 오지상승위치료법 ······ 14
6.3.1 오지상승위치료법 ······ 14
7. 관찰항목, 관찰검사 방법 및 일정 ······ 17
7.1 진행일정표 ······ 17
7.2 관찰항목별 방법 ······ 18
7.2.1 피험자 동의서 서명 및 스크리닝 번호 부여 ······ 18
7.2.2 인구학적 조사 및 병력 조사 ······ 18
7.2.3 피험자 적합성 평가 ······ 18
7.2.4. 피험자 식별코드 부여 ······ 18
7.2.5 임상검사항목 ······ 18
7.2.6 순응도 평가 ······ 24
7.2.7 이상반응 조사 ······ 24

7.3 관찰검사 방법 및 일정 ··· 24
7.3.1 선정방문과 스크리닝 ··· 24
7.3.2 시험시행 ··· 24
8. 예측 부작용 ··· 27
9. 중지 및 탈락 기준 ··· 28
9.1 중지의 정의 ··· 28
9.2 중지의 기준 ··· 28
9.3 탈락의 정의 ··· 28
9.4 탈락의 기준 ··· 28
9.5 중지 및 탈락의 처리 과정 ··· 28
9.6 순응도와 임상시험 계획서 위반에 대한 처리 ··· 28
10. 평가기준, 평가방법 ··· 29
10.1. 평가기준 ··· 29
10.1.1 PTSD환자 대상 오지상승위치료법과 VR 기반 오지상승위치료법 전후 평가 분석 ··· 29
10.1.2 정량화뇌파(QEEG) 분석 ··· 29
11. 통계분석 방법 ··· 29
11.1. 인구학적 자료와 시험 시작 시 임상병력 자료의 분석 ··· 29
11.2. 평가변수의 분석방법 ··· 30
11.2.1 PTSD환자 대상 오지상승위치료법과 VR 기반 오지상승위치료법의 효과성 분석 ··· 30
11.2.2 PTSD환자 대상 오지상승위치료법과 VR 기반 오지상승위치료법 전후 측정값들 간의 상관성 분석 ··· 31
11.3. 안전성 변수의 분석방법 ··· 32
12. 이상반응을 포함한 안전성의 평가기준, 평가방법 및 보고방법 ··· 32
12.1 안정성 관련 용어의 정의 ··· 33
12.1.1 이상반응 ··· 33
12.1.2 중대한 이상반응 ··· 33
12.2 이상반응의 평가 ··· 33
12.2.1 중증도 평가 ··· 33
12.2.2 시험치료법과의 인과관계 평가 ··· 33
12.3 안전성 통계처리 ··· 34
12.4 이상반응 보고 ··· 34
12.4.1 이상반응 교육 ··· 34
12.4.2 중대한 이상반응의 보고 ··· 34
12.4.3 이상반응 발생 시 조치사항 ··· 34
12.4.4 이상반응의 추적관찰 ··· 34
13. 피험자에 대한 이익 및 혜택 ··· 35
14. 피험자의 안전 보호에 관한 대책 ··· 35
14.1 시험자의 역할 ··· 35
14.2 모니터링의 역할 ··· 35
14.3 피험자 동의 ··· 35

14.4 임상시험계획서의 승인과 변경 ······ 36
14.5 피험자 기록의 비밀보장 ······ 36
14.6 자료의 보관 ······ 36
15. 참고문헌 ······ 37

1. 임상시험의 명칭 및 단계

1.1 명칭

외상후 스트레스 장애 환자에 대한 가상현실 기반 오지상승위치료법의 안전성, 유효성 및 경제성 평가 임상시험

1.2 단계

해당없음

2. 임상시험 실시기관, 임상시험책임자/담당자 및 의뢰자

2.1 주관기관 및 주소

원광대학교 산학협력단
전북 익산시 익산대로 460 원광대학교 대학본부 4층 산학협력단
전화: 063-850-5952

2.2 실시기관 및 주소

원광대학교 산본병원
경기도 군포시 산본로 321

2.3 임상시험 의뢰자 및 주소

한국보건산업진흥원
충북 청주시 흥덕구 오송읍 오송생명2로 187 오송보건의료행정타운 한국보건산업진흥원
전화: 034-713-8000

3. 임상시험의 목적 및 배경

3.1 임상시험의 목적

○ 본 연구는 외상후스트레스장애 환자에 대한 가상현실 기반 오지상승위치료법의 유효성, 안전성 및 경제성 평가를 통해 임상 적용 가능성을 탐색하고 가상현실 기반의 한의 치료모델을 제시하기 위함이다.

3.2 임상시험의 배경

○ 외상 후 스트레스 장애 개요

외상 후 스트레스 장애(Posttraumatic stress disorder, PTSD)는 죽음, 심각한 상해, 성폭력과 같은 외상성 사건에 노출된 이후 다음과 같은 특징적인 증상이 나타나는 질환이다. 외상성 사건에 대한 반복적이고 고통스러운 기억, 꿈과 같은 침습 증상, 외상성 사건과 관련이 있는 자극에 대한 지속적인 회피, 외상성 사건과 관련이 있는 인지와 감정의 부정적인 변화, 외상성 사건과 관련된 각성과 반응성의 뚜렷한 변화가 나타난다 [1]. 2016년 발표된 정신질환실태 역학조사 보고서에 따르면, PTSD의 평생 유병률은 1.5%라고 보고되었다 [2]. PTSD 환자들은 동시에 우울장애, 물질사용장애, 불안장애 등을 같이 가지고 있는 경우가 많다

[3].

PTSD의 치료를 위하여 첫번째로 고려되는 것은 외상에 초점을 맞춘 심리치료이다 [4, 5]. PTSD의 심리치료로 인지행동치료/인지치료, 인지처리치료, 노출치료/지속노출치료, 안구운동 민감소실 및 재처리(EMDR), 내러티브 노출치료, 스트레스 면역훈련, 단계기반치료 등이 적용되고 있다 [6]. 한의정신요법으로는 경자평지요법, 지언고론요법, 이정변기요법, 오지상승요법 등을 적용할 수 있으며 [7] 한의정신요법 기반 PTSD 심리치료 프로그램 또한 개발된 바 있다 [8].

○ PTSD를 포함한 정신건강 관리에 적합한 기술인 가상현실(VR)
- 가상현실(VR)은 불안과 우울과 같은 정신적인 스트레스를 해소하기 위한 교육 및 훈련에 유용한 기술이다. 1990년대에 고소공포증 치료에 VR을 이용한 이후, 정신질환, 스트레스 및 통증 경감 등의 연구에서 활용되어 왔다.
- COVID-19 대유행으로 인하여 2020년을 기점으로 다양한 분야에서 VR활용이 증가하고 있다, 특히 대면접촉이 필수적이었던 심리치료 분야에서 VRT 사용에 대한 관심이 증가하고 있다.
- 미국을 중심으로 다양한 분야에서 VRT(Virtual Reality Therapy)적용의 증가하는 추세로 특히, 정신과 치료 영역에서 급증하였다. VR기반 심리치료 적용을 다양한 정신질환의 진단부터 PTSD 치료부터 중독 및 신경증의 우울, 불안에까지 다양하게 진행하고 있다 [26][27].
- PTSD 환자(세계무역센터의 공격이나 전쟁장면을 경험한)에게 실제에서 적용하기 불가능한 상황을 재연함으로써 상상의 대안적인 기법으로 사용하고 있다. 특히 PTSD 환자들의 경우 VR을 적용할 경우 통제가능한 환경에서의 환자의 수준에 맞는 치료를 할 수 있어서 실제 노출의 위험성을 줄이고 치료적 효과를 증대할 수 있음이 지속적으로 보고되고 있다.

○ VR 기술의 정신치료 적용의 한계와 극복방안
- 최근에는 과학기술의 발달과 사회적 환경에 의해 비대면 치료적 개입에 대한 관심이 증가하고 있다 [28].
- 가상현실 심리치료의 장점은 현실과 유사한 가상공간에서 내담자의 감정이나 사고방식, 정서 상태 등을 변화하게 하여 심리적 고통과 정서적 불안을 가지고 있는 환자를 치료한다는 것이다 [29].
- 하지만, 1990년대 초창기 이후 외상후스트레스장애, 공포증, 불안증 컨텐츠 상용화 하는 데 실패하였는데, 이는 VR 기술의 한계도 가지고 있는 심리치료기술의 콘텐츠의 빈약이 이유로 사료된다.
- 한국 문화의 풍부한 콘텐츠는 심리치료 활용에 좋은 매개체이고, 한의학 정신치료와 VR 기술을 접목하여 이런 콘텐츠를 활용할 수 있다면 VR 기술의 정신치료 분야에 새로운 전기가 될 수 있다.
- 한의정신요법 중 오지상승위치료법은 한의학 고유의 정신치료법으로서 서양에서는 없는 오행상생상극 이론에 따른 감정치료기법이다. 한의학에서 감정은 칠정(七情)으로 외계 환경이나 정서적인 자극에 대한 감정반응으로, 칠정에 문제가 있어 정신증상 뿐만 아니라 신체증상까지 발현되며, 이를 칠정상(七情傷)이라 하는데[30], 칠정(七情)은 한의학에서 인간 정신에 대한 생리·병리의 근간이자 치료적 핵심으로, 오장기능을 조절함으로써 오장에 배속된 감정증상을 치료할 수 있다.
- 본 연구에서는 오지상승위치료법을 VR기술과 접목한 VR기반 오지상승위치료법을 PTSD 환자 대상으로 수행하여 효과 및 안전성, 경제성을 탐색하여 임상적 적용 가능성에 대해 탐색하고자 한다.

3.3 연구 내용, 범위 및 방법

○ 피험자 선정
  - 본 임상시험에서는 공개모집을 통하여 자의에 의해 임상시험 동의서에 서명한 피험자를 대상으로 하여 임상시험 계획서에 따라 선정기준 및 제외기준을 검토하여 본 임상시험에 적합하다고 판단되면 피

험자로 선정한다.

○ **블록무작위배정**(Blocked Randomization)
- 임상시험의 수행 및 평가에 관여하지 않는 무작위배정 담당자(또는 통계 담당자)가 구체적 계획 및 재현 가능한 방법으로 무작위배정 목록을 생성하고 무작위 배정표는 봉인하여 봉인해제 여부를 확인할 수 있는 방식으로 보관하며 연구책임자가 별도로 관리한다.
- 무작위배정 담당자는 통계프로그램 StataMP 16 program(StataCorp LLC, 4905 Lakeway Drive College Station, Texas 77845 USA)을 사용하여 각 개체가 뽑힐 가능성이 동일한 상태에서 무작위로 각 군에 배정한다. 시험군 1, 2와 대조군의 무작위 배정은 1:1:1 비율로 하는 블록 무작위배정 방법(block randomization) 실시한다.
- 시험담당자는 대상자 앞에서 순서대로 무작위배정 봉투를 개봉하여 대상자를 배정하고 개봉한 봉투에는 개봉한 날짜와 시험담당자의 서명을 기재하여 별도 보관한다.
- 무작위배정 봉투와 무작위 배정표에 기재된 대상자 식별코드는 무작위 배정 번호를 의미한다.

○ **시험군 1,2**(VR오지상승위개입군, 오지상승위개입군)**와 대조군 평가**
- 시험군 1,2와 대조군 모두 기초설문으로 경계정충 변증도구를 시행한다. 시험군1은 가상현실(Virtual Reality, 이하 VR) 기반 오지상승위치료법을, 시험군2는 오지상승위치료법을 실시하고 대조군은 별다른 프로그램을 시행하지 않는다.
- 오지상승위치료법 혹은 가상현실 기반 오지상승위치료법을 8주간 실시하고 0주, 8주, 12주 Follow up에 시험군과 대기대조군에게 주 유효성 평가 도구, 부 유효성 평가도구 설문평가를 실시힌다. 경제성 평가에 대한 설문평가는 0주, 4주, 12주 Follow에 실시한다. 기기검사는 0주, 8주에 측정한다.
- 주 유효성 평가는 PCL-5-K를, 부 유효성 평가로는 CGI-I, CSEI-S, K-PTGI, FFMQ-S, PHQ-15, HADS, ISI-K, 화병척도, SUDS, MRM을 실시 평가한다. 단, CSEI-S, SUDS, MRM은 매회기 전후로 평가한다.
- 경제성 평가로는 EQ-5D, EQ-VAS, 비용측정 설문을 실시한다.
- 기기검사로 심박변이도(HRV)와 정량화 뇌파(QEEQ)분석을 실시하고, 정량화 뇌파 측정은 중재 전후로 눈 감은 상태 5분, 눈 뜬 상태 5분 총 10분간 측정한다.
- 모든 실험이 완료된 이후 원하는 대조군은 Follow up 이후 오지상승위치료법 혹은 가상현실 기반 오지상승위치료법을 실시한다.

○ 평가방법 절차
- 각 평가는 시험군1과 시험군 2 및 대조군 세군간과 세군 내 효과성을 분석하기 위해 차이검증 및 상관관계 분석을 실시한다.
- 이후 VR기반 오지상승위요법과, 오지상승위요법 각각의 비용-효용대비를 확인하기 위한 경제성 평가를 실시한다.

3.4 연구결과의 기대효과 및 활용

○ 본 연구 수행을 통하여 한방정신요법을 가상현실과 접목한 ‘가상현실 기반 오지상승위치료법’을 통해 한방정신요법을 보다 효과적으로 활용할 수 있는 새로운 기술 개발의 사례를 제시할 수 있다.

○ PTSD 등의 정신과 질환에 대한 VR과 같은 신기술을 활용한 치료법 개발의 가능성과 기초근거를 제시할 수 있다.

○ 가상현실 기반 오지상승위치료법 전후의 주유효성 평가 척도와 부유효성 평가 척도와 심박변이도 및 뇌파변화의 상관성을 규명함으로써, 외상후 스트레스장애의 가상현실 기반 오지상승위치료법에 대한 각

효과성을 예측할 수 있다.

○ 한의치료를 통한 PTSD 증상 개선으로 환자, 가정 및 사회구성원의 삶의 질 향상을 도모할 수 있다.

4. 대상 질환 및 임상시험 기간

4.1 대상 질환

SCID-5(Structured Clinical Interview for DSM-5)에 의해 PTSD로 진단받은 자

4.2 임상시험 기간

임상시험 승인일로부터 2026년 12월 31일까지

5. 피험자의 선정기준, 제외기준 및 목표 피험자의 수와 선정근거

5.1 선정기준

다음 사항에 모두 해당하는 환자를 시험에 적합한 자로 선정한다.

1) 만 18세 이상 65세 미만 성인 남녀
2) 구조화된 임상적 면담 도구(SCID-5, Structured Clinical Interview for DSM-5)에 의해 외상 후 스트레스 장애로 진단받은 자
3) PC-PTSD-5 3점 이상인 자
4) 자의로 참여를 결정하고 동의서에 서명한 자

5.2 제외기준

다음 사항에 해당하는 환자는 제외한다.

1) 고도의 정신과 질환자
   ① 최근 또는 과거병력 상 망상 또는 환각을 갖고 있는 경우
   ② 적어도 조증이 한 번 이상 나타난 경우
   ③ 알코올 남용이나 의존 경력이 있는 경우
   ④ 자살위험이 있는 자
2) 병용약물을 제외하고 증상유발에 영향을 줄 것으로 판단되는 물질을 지속적으로 투여하여야 하는 경우
3) 선천적 질환, 중추신경계, 말초신경계 질환, 내분비계 질환, 면역학적 질환, 심, 간, 신에 심각한 문제가 있는 자
4) 임신 중이거나 수유 중인 자
5) 지난 1달 간 다른 임상연구에 참여한 경험이 있거나 현재 다른 임상연구에 참여 중인 자.
6) 임상시험 담당자 혹은 임상시험 책임자가 기타 임상시험을 수행하기 어렵다고 판단되는 자

5.3 병용약물

시험기간 중 VR 기반 오지상승위치료법 혹은 오지상승위치료법 이외의 다른 비약물적 처치는 시행하지 않는 것을 원칙으로 하지만 다음과 같은 비약물적 처치와 병용약물경우는 참가자격부여가 가능하다. 다만, 필요한 경우에는 다음의 기준에 따른 시험담당자의 판단에 의해 실시될 수 있다.

1) 기존 PTSD을 위해 실시한 상담치료는 screening 전 최소 3개월 동안 안정적으로 실시해온 경우
2) Hypnotics, antidepressants, antipsychotics, anxiolytics 복용환자의 경우 최근 4주 동안 약물용량이 stable한 경우

3) 임상시험 이전부터 상복하던 다른 질환의 치료약물(고혈압, 당뇨, 고지혈증, 진통제 등)은 모두 허용한다.

5.4 목표한 피험자의 수 및 산출근거

유효성 평가 피험자 수: 60명, 유효성 평가 피험자수: 20/군

탈락률 20% 고려하여 최종 배정 피험자수: 24/군, 목표 피험자 수: 72명

♠ 피험자 수 결정 근거

1) G*Power 3.1.9.4 정량적 산출

G*Power 3.1.9.4 의 정량적 산출 결과 Effect size는 0.5, Power는 95% 신뢰구간, 세 집단으로 설정하고, 공변량 분석 시 필요한 대상자수는 65명으로 나왔다. 이는 본 연구에서 제시한 60명에 ±10%이내 피험자수인 72명의 유의성을 신뢰롭게 확보할 수 있는 통계적 근거이다.

F tests – ANCOVA: Fixed effects, main effects and interactions

Analysis: A priori: Compute required sample size

**Input:**	Effect size f	=	0.5
	α err prob	=	0.05
	Power (1-β err prob)	=	0.95
	Numerator df	=	2
	Number of groups	=	3
	Number of covariates	=	1
**Output:**	Noncentrality parameter ë	=	16.2500000
	Critical F	=	3.1477912
	Denominator df	=	61
	Total sample size	=	65
	Actual power	=	0.9502209

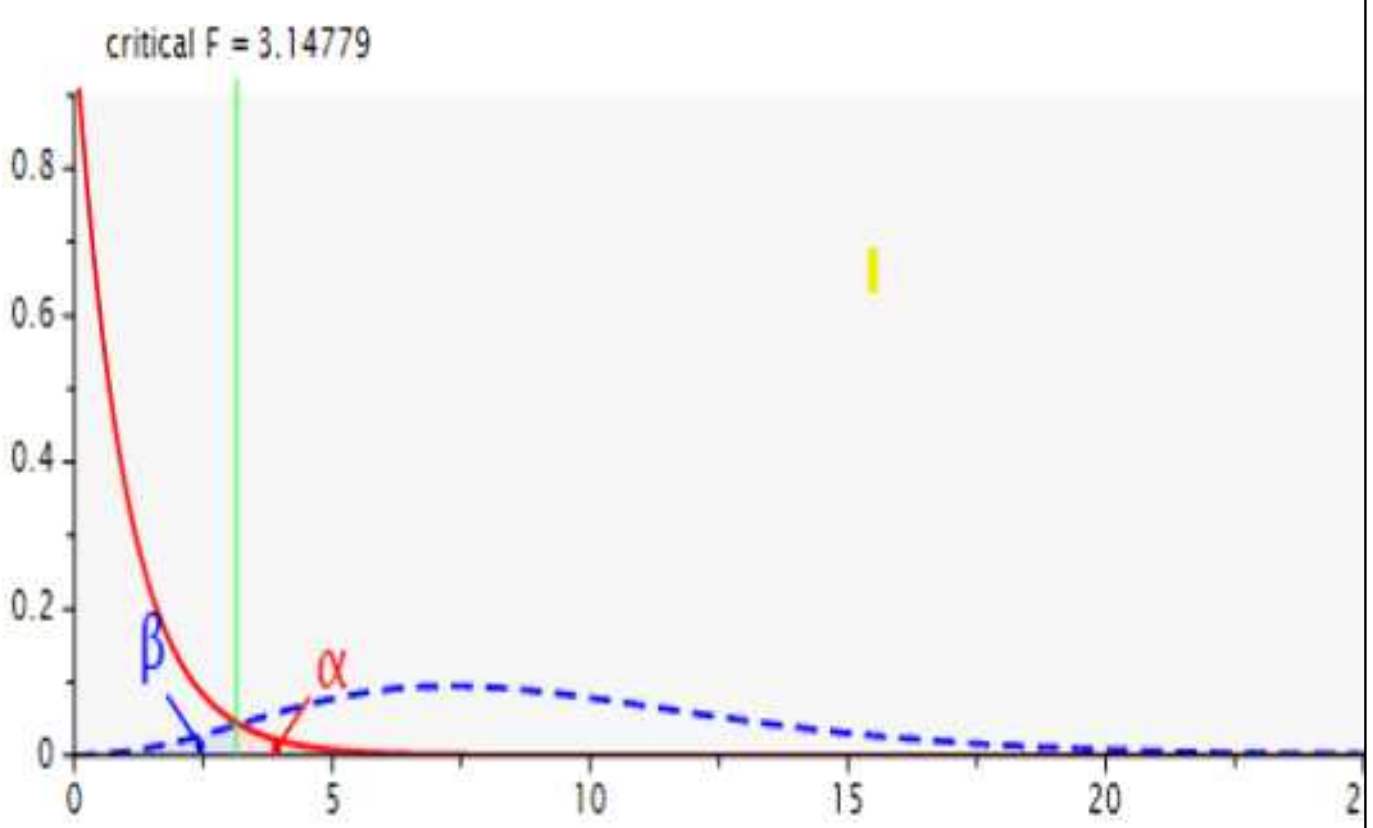

2) 선행연구 근거

Sebastian, B., &Nelms, J. (2017)[41]의 PTSD대상 EFT 효과성을 분석한 메타분석결과에서 선행연구들의 연구대상자 수는 가장 적은 수가 8명에서 가장 많은 수가 32명으로 나타났다.

대부분의 연구들은 10명이내의 연구대상자를 대상으로 연구를 진행하여 그 효과성을 분석하였다.

이에 본 연구에서 각 군당 20명의 피험자 수는 그 이론적 근거를 확보할 수 있는 가능성이 높다.

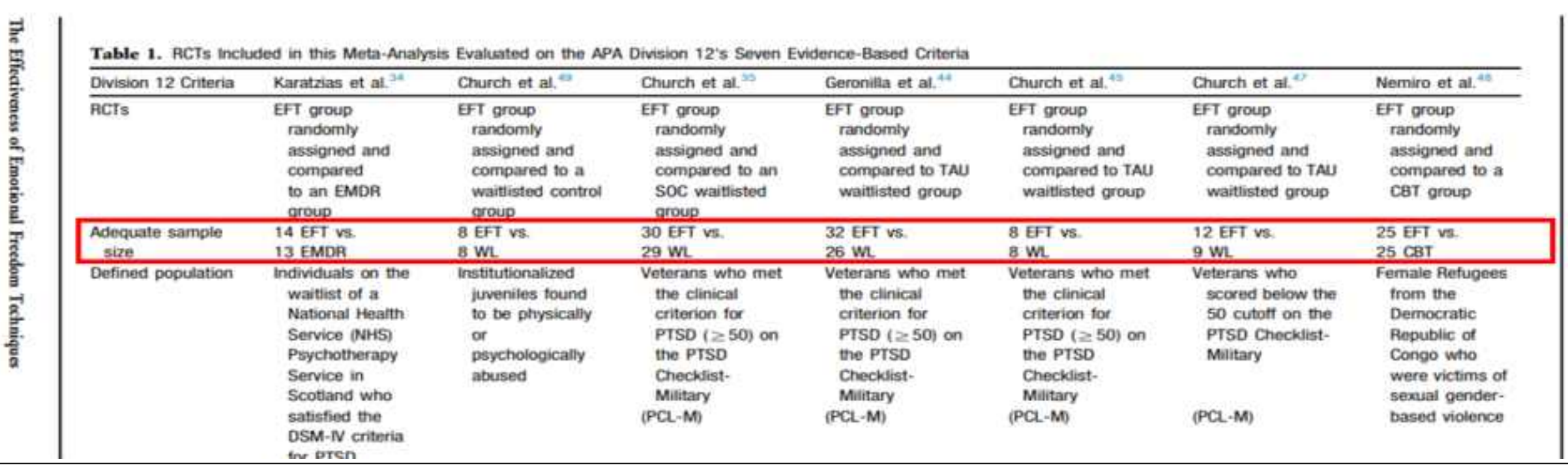

The Effectiveness of Emotional Freedom Techniques

Table 1. RCTs Included in this Meta-Analysis Evaluated on the APA Division 12's Seven Evidence-Based Criteria

Division 12 Criteria	Karatzias et al.[34]	Church et al.[49]	Church et al.[35]	Geronilla et al.[44]	Church et al.[45]	Church et al.[47]	Nemiro et al.[48]
RCTs	EFT group randomly assigned and compared to an EMDR group	EFT group randomly assigned and compared to a waitlisted control group	EFT group randomly assigned and compared to an SOC waitlisted group	EFT group randomly assigned and compared to TAU waitlisted group	EFT group randomly assigned and compared to TAU waitlisted group	EFT group randomly assigned and compared to TAU waitlisted group	EFT group randomly assigned and compared to a CBT group
Adequate sample size	14 EFT vs. 13 EMDR	8 EFT vs. 8 WL	30 EFT vs. 29 WL	32 EFT vs. 26 WL	8 EFT vs. 8 WL	12 EFT vs. 9 WL	25 EFT vs. 25 CBT
Defined population	Individuals on the waitlist of a National Health Service (NHS) Psychotherapy Service in Scotland who satisfied the DSM-IV criteria for PTSD	Institutionalized juveniles found to be physically or psychologically abused	Veterans who met the clinical criterion for PTSD (≥50) on the PTSD Checklist-Military (PCL-M)	Veterans who met the clinical criterion for PTSD (≥50) on the PTSD Checklist-Military (PCL-M)	Veterans who met the clinical criterion for PTSD (≥50) on the PTSD Checklist-Military (PCL-M)	Veterans who scored below the 50 cutoff on the PTSD Checklist-Military (PCL-M)	Female Refugees from the Democratic Republic of Congo who were victims of sexual gender-based violence

Sebastian, B., &Nelms, J. (2017). The effectiveness of Emotional Freedom Techniques in the treatment of posttraumatic stress disorder: A meta-analysis. *Explore*, *13*(1), 16-25.

6. 임상시험 방법

6.1 임상시험의 설계

-무작위배정, 대조군, 단일기관 임상시험

-최종 선정된 PTSD 환자를 대상으로 블록무작위배정에 따라 VR기반 오지상승위개입군(이하 시험군 1), 오지상승위개입군(이하 시험군 2)과 대조군으로 나눈다. 시험군 1은 주 2회 회당 40분, 8주간의 VR기반 오지상승위치료를 실시하고 시험군 2는 오지상승위치료를 시행한다. Follow up은 12주 후에 한다. 대조군은 8주간 평소 일상생활을 하면서 대기한다. 대기하는 동안 스트레스 상황의 변화여부를 2주마다 전화로 check하고, 0, 4, 8주째 내원하여 시험군과 같은 검사 및 설문지를 평가한다. Follow up은 12주 후에 내원하여 실시한다.

-시험군1과 2에 각각 **VR기반 오지상승위치료법과 오지상승위치료법의** 효과를 알아보기 위하여 중재 전후 심박변이도(HRV), 정량화 뇌파(qEEG)분석과 주유효성 평가지와 부유효성 평가지 설문을 실시한다.

-시험군 1과 시험군 2에 VR기반 오지상승위치료법과 오지상승위치료법의 효과와 각 중재 전후 심박변이도(HRV), 정량화 뇌파(qEEG) 결과와 주유효성 평가지와 부유효성 평가지의 중재 전후 PTSD에 영향을 미치는 예측 인자를 확인 할 수 있도록 상관성 분석을 실시한다.

-시험군 1과 시험군 2를 대상으로 오지상승위치료와 VR기반 오지상승위치료가 1단위 증가할 때마다 EQ-5D(EuroQoL-5D), EQ-VAS의 변화정도와 비용측정 설문을 통해 오지상승치료와 인지행동 치료의 유사성을 근거로 비용-효용 분석 경제성 평가를 실시한다.

6.2 임상시험 설계 요약

가. 임상시험 설계 단계

-본 임상시험에 참여를 신청한 지원자 중에서 Structured Clinical Interview for DSM-5에 의해 PTSD로 진단받은 자(이하 SCID-5)를 대상자로 선정한다.
-선정 이후 기초설문으로 경계정충 변증도구를 시행한다.
-시험군1에 VR기반 오지상승위치료를 주 2회 회당 40분, 8주간 실시하고 Follow up은 12주 후에 한다. 시험군2에 오지상승위치료를 주 2회 회당 40분, 8주간 실시하고 Follow up은 12주 후에 한다. 대조군은 8주간 평소 일상생활을 하면서 대기한다. 대기하는 동안 불안 및 스트레스 상황의 변화 여부를 2주마다 전화로 check하고, 0, 4, 8주째 내원하여 시험군과 같은 검사 및 설문지를 평가한다. Follow up은 12주 후에 내원하여 실시한다.
-4주(8회기), 8주(16회기), 개입 종료후 4주 뒤인 12주차 Follow up 시점에서 피험자를 대상으로 하여 이상 반응이 있는지 확인한다.

나. 임상시험 설문 및 측정평가

- **기초설문으로서** 의료진에 의해 0주차 경계정충 변증도구를 탐색한다.
-**증상평가는** 시험자 1과 2군 및 대기대조군 대상 0주차 1회기, 8주차 16회기, Follow up 12주차에 주 평가 설문지 PCL-5-K, 부 평가 설문지 CGI-I, K-PTGI, FFMQ-S, PHQ-15, HADS, ISI-K, 화병척도를

사용하여 측정한다.

-**경제성평가를 실시하기** 위해서 0주차 1회기, 4주차 8회기, 8주차 16회기, Follow up 12주차에 시험군 1과 2, 대기대조군 대상 설문지인 EQ-5D, EQ-VAS, 비용측정 설문을 실시한다.

-**매 회기 전후 평가는** 피험자의 안정과 변화 관찰을 위해 시험군 1과 2를 대상으로 중재 전후 마음의 방그리기(MRM), CSEI-S, SUDS를 실시한다.

-**PTSD로 진단받은 시험자 1과 2군 및 대기대조군을** 대상으로 하여 0(1회), 8주(16회)에 HRV분석을 실시하여, 심박수 변이를 측정한다. 정량화된 뇌파(Quantitative Electroencephalogram)를 측정하여 피험자대상의 각 주파수대별 absolute power(μV2), relative power(%)와 각각의 Z-score, 주파수대별 L 측정값을 확인한다.

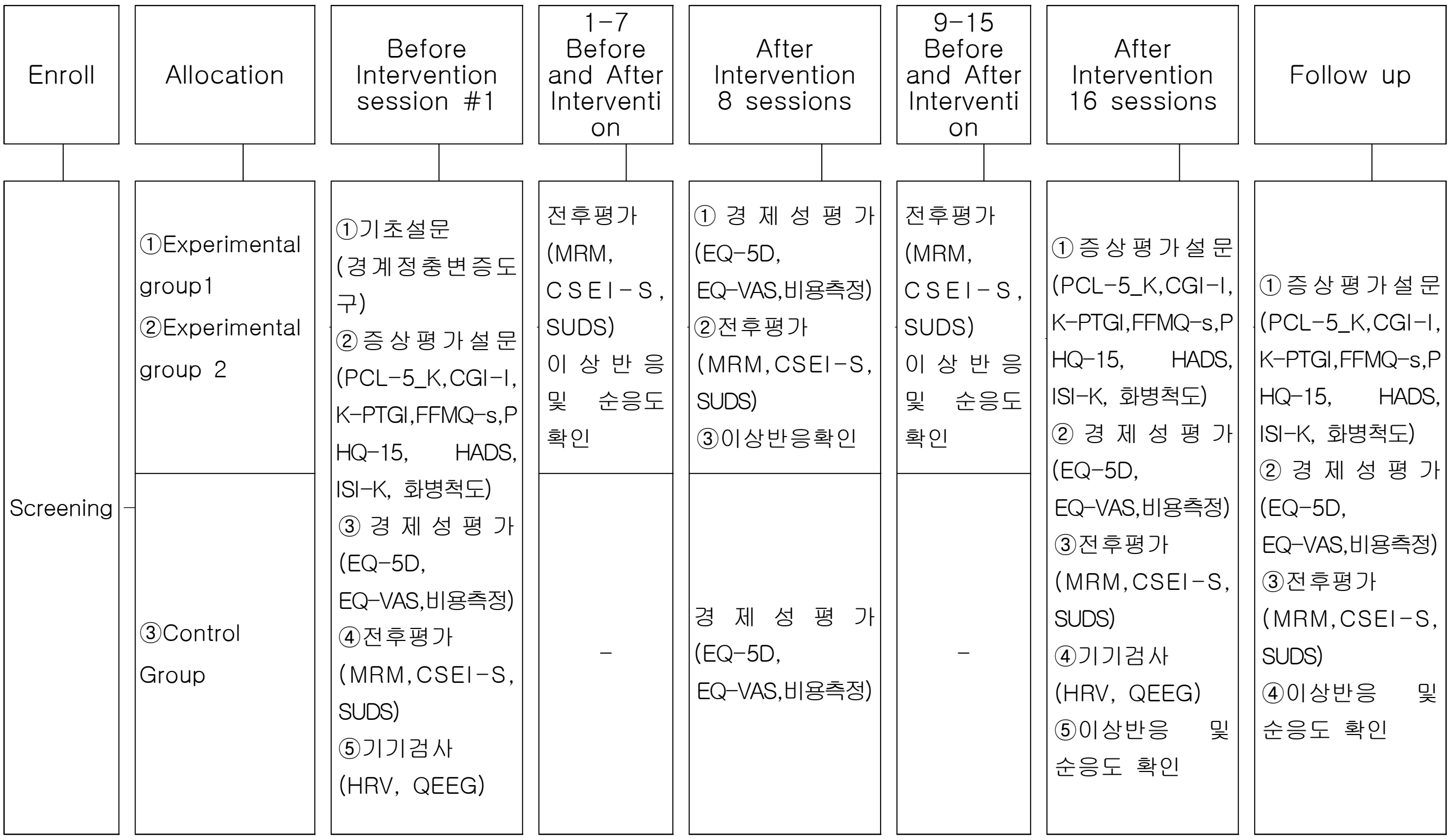

Figure 4. Summary of clinical trial design.

PCL-5-K: The Posttraumatic Stress Disorder Checklist for DSM-5	ISI-K; Korean version of Insomnia severity index
K-PTGI; Korea- Posttraumatic Growth Inventory-Short Form	CSEI-S; The Core Seven-Emotions Inventory Short Form
CGI-I; The Clinical Global Impression-Improvement scale	SUDS; Subjective Units of Distress Scale
FFMQ-S; Five Facet Mindfulness Questionnaire Short Form	MRM; Mentalizing the Rooms of Mind
PHQ-15; Patient Health Questionnaire-15	EQ-5D; EuroQoL-5Dimension
HADS; Hospital Anxiety and Depression Scale	EQ-VAS; EuroQoL-visual analog scale
	HRV; Heart Rate Variation
	QEEG; Quantative Encephalogram

6.3 PTSD환자 대상 오지상승위치료법과 VR 기반 오지상승위치료법

6.3.1 오지상승위치료법

- 한의학의 기본이론인 오행상생상극이론을 심리치료에 응용한 한의학 고유의 정신요법이다. 오장에 각각의 감정을 배정하고, 그 감정은 역동적으로 상호간에 영향을 미친다. 이러한 역동적 관계를 조절함으로써 치료하며, 환자의 정서 및 감정을 분석하고, 오행상에서 이 정서와 감정에 대하여 상대되는 정서 및 감정을 이용하여 환자로 하여금 안정된 상태가 되도록 도와주는 방법으로 진행한다.
- 즉, 외부환경이나 정서적인 자극을 받으면 환자는 과다하게 치우친 병리적 상태의 정서를 보이는데, 치료자가 환자로 하여금 다른 특정한 정서를 유발시켜 환자의 병리적 정서를 조절하고자 하는 기법이다.

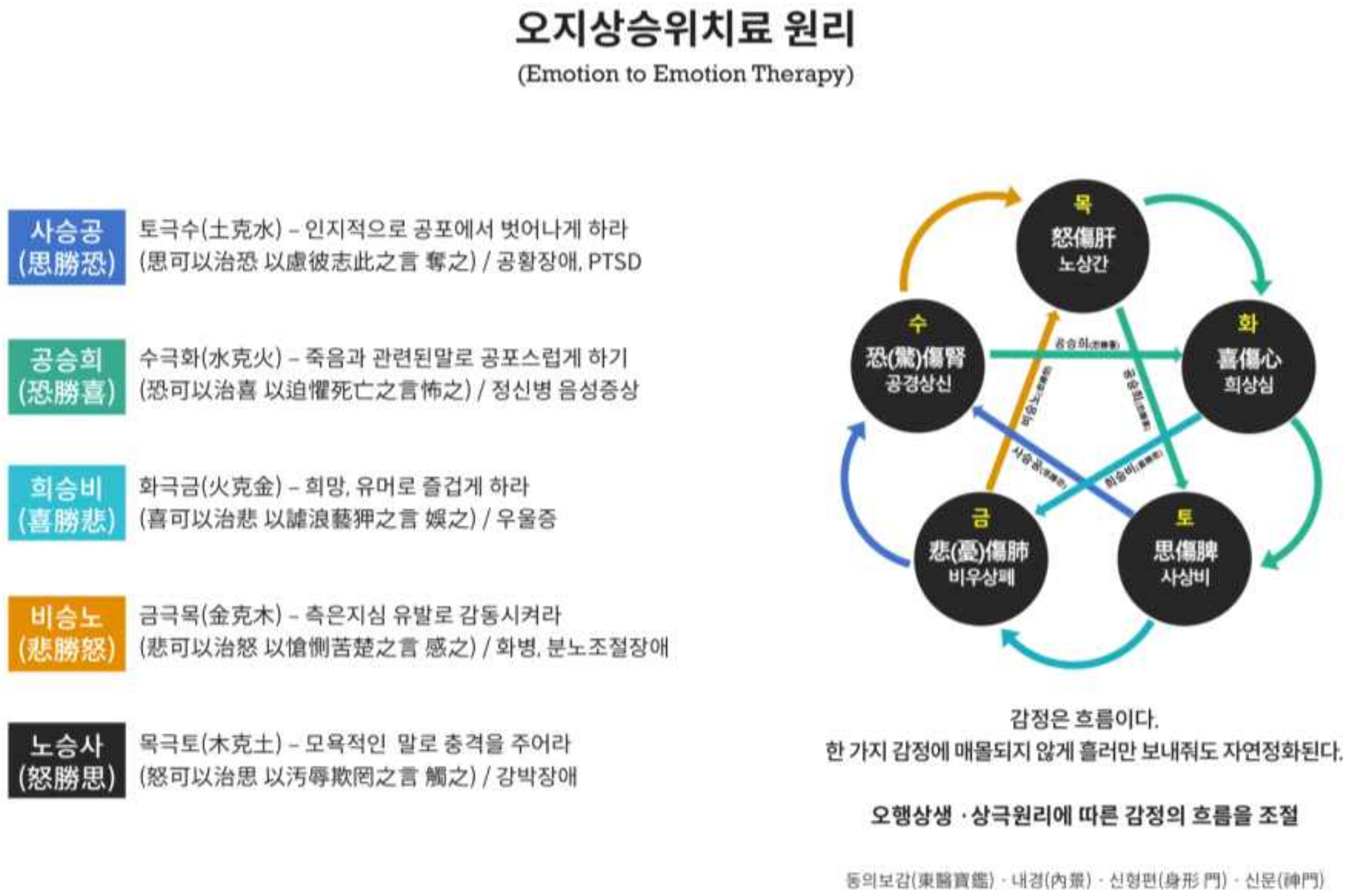

○ 오지상승위치료법 시행방법

- 오지상승위치료법은 평가, 분석, 훈련, 재평가, 교육의 단계로 이루어진다. (총 40분)
  (1) 평가 : 환자의 정서 및 감정 평가, 신체증상 평가 (CSEI-S, MRM, SUDS 평가) (5분)
  (2) 분석 : 감정과 신체증상과의 연관성, 감정과 감각의 음양오행적 분석, 핵심감정 확인, 오지상승치료법 선택 (5분)
  (3) 훈련 : 감정유발 명상, 호흡명상, 신체감각 명상, 상단전 명상, 희희명상, 하단전 명상, 중단전 명상, 리소스 명상, 통합-삼단전 명상 (20분)
  (4) 평가 : 훈련 후 현재 감정 및 신체증상 평가 (CSEI-S, MRM, SUDS 평가) (5분)
  (5) 감정조절 교육 (5분)

[오지상승위치료법(ETE therapy) 진행 순서 및 내용]

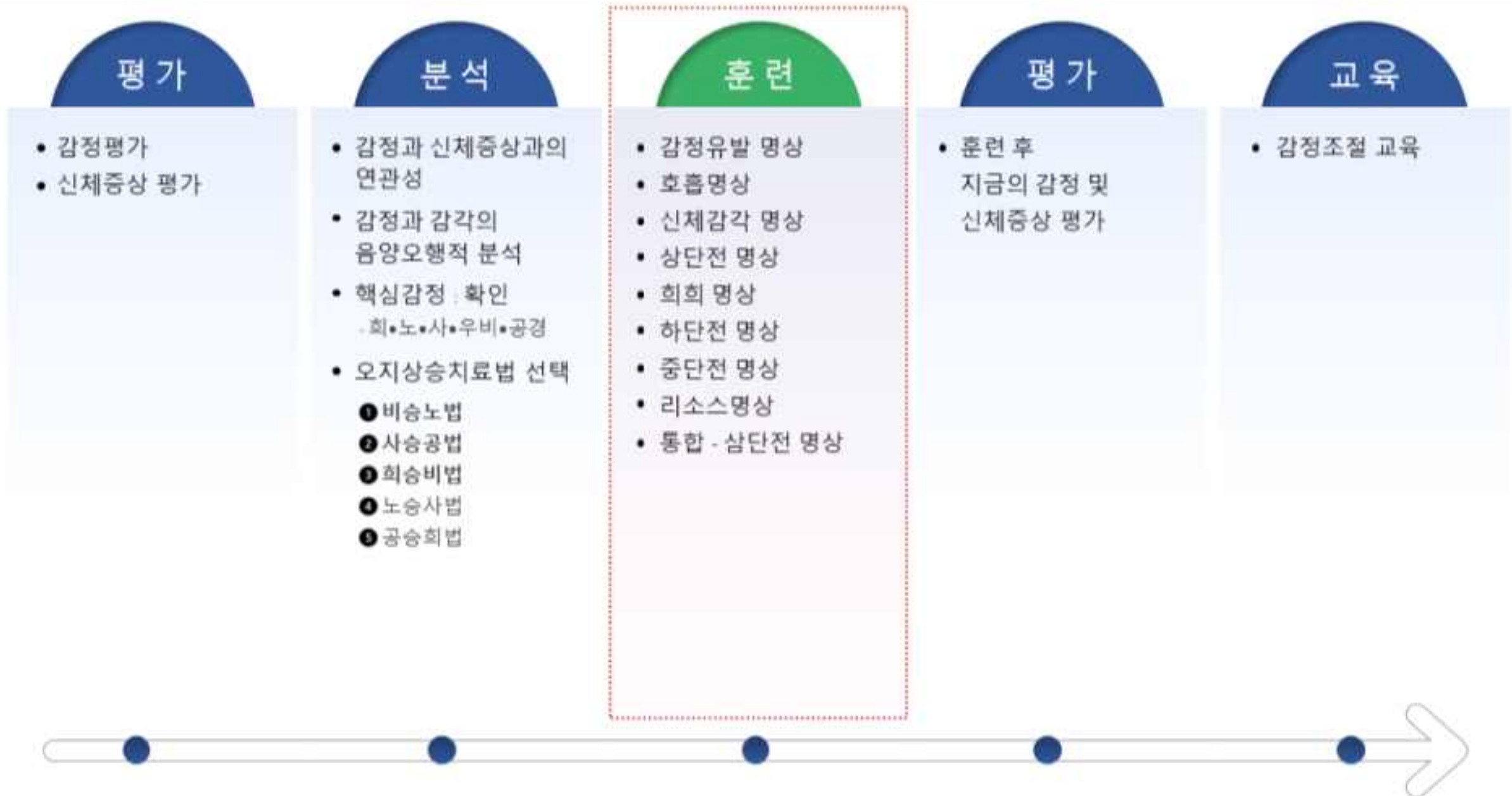

- PTSD환자의 경우 공포가 특징적인 정서로, 오지상승위치료법 중 사승공(思勝恐) 위주로 활용하여 인지적으로 공포에서 벗어나도록 하며, 훈련에서는 상단전 명상에 해당한다. 상단전 명상의 내용은 다음의 표와 같다.

<상단전 마음챙김 명상 가이드><br><br>우선 내 몸이 원하는 편안한 자세를 찾아봅니다.<br>등을 펴고 손을 가볍게 무릎 위에 얹습니다.<br>그리고 최대한 몸에 힘을 빼봅니다.<br>양어깨에 힘이 빠지고 두 손, 손가락도 그대로 내려놓습니다.<br>내 몸이 이완되어 가는 것을 그냥 있는 그대로 느껴보고<br>내 몸이 이렇게 느슨해지는 것을 알아차립니다<br><br>이번에는 잠시 호흡으로 의식을 가져가 봅니다.<br>자연스러운 형태로<br>들이쉬는 숨과 내쉬는 숨의 흐름을 따라가 봅니다.<br>숨이 코로 들어와서 깊게 들어갑니다.<br><br>그리고 천천히... 내 쉽니다. 후~~ (소리내면서)<br>다시 깊게 들이마시고, 천천히 후~~ 내쉽니다.<br>이 자연스러운 호흡의 흐름을 그대로 따라갑니다.<br><br>이제, 이마의 한가운데 상단전으로 의식을 옮겨봅니다.<br>상단전은 알아차리는 자리입니다.<br>통찰하는 자리입니다.<br><br>상단전에 가만히 집중하면서 알아차려지거나. 깨달아진것이 있으면<br>그것을 그대로 언어화 해봅니다.<br><br>아~ 이런거구나?

아~ 이랬구나?<br>이거였구나?<br><br>언어화된 그것을 그대로 그곳에 저장합니다.<br><br>저장된 이 느낌은 당신이 필요하다면 언제든지 다시 느낄 수가 있습니다. 통찰과 알아차림이 있는 내 상단전의 느낌입니다.<br><br>천천히 눈을 뜨고 원래 자리로 돌아옵니다

- 상단전 명상 외에도 환자의 정서상태에 따라 다른 훈련 명상을 시행할 수 있다.

○ VR 기반 오지상승위치료법 시행방법

- VR 기반 오지상승위치료법은 Head Mounted Display(HMD) 혹은 컴퓨터 및 프로젝트 세트를 통해 가상현실을 구현할 수 있는 소프트웨어를 사용하여 진료실 환경에 제한되지 않고 현실감 있게 구현된 가상환경에서 문제 감정에 대한 상극감정으로 감정을 안정화시키는 훈련을 실시하는 방법이다. 기존의 데스크탑 컴퓨터나 기어 VR과 같은 상용화 장비에 오지상승위치료법 콘텐츠가 포함된 소프트웨어, 어플리케이션 등을 설치하여 사용한다.

[PTSD의 VR 기반 오지상승위치료법(ETE therapy) 진행 순서 및 내용]

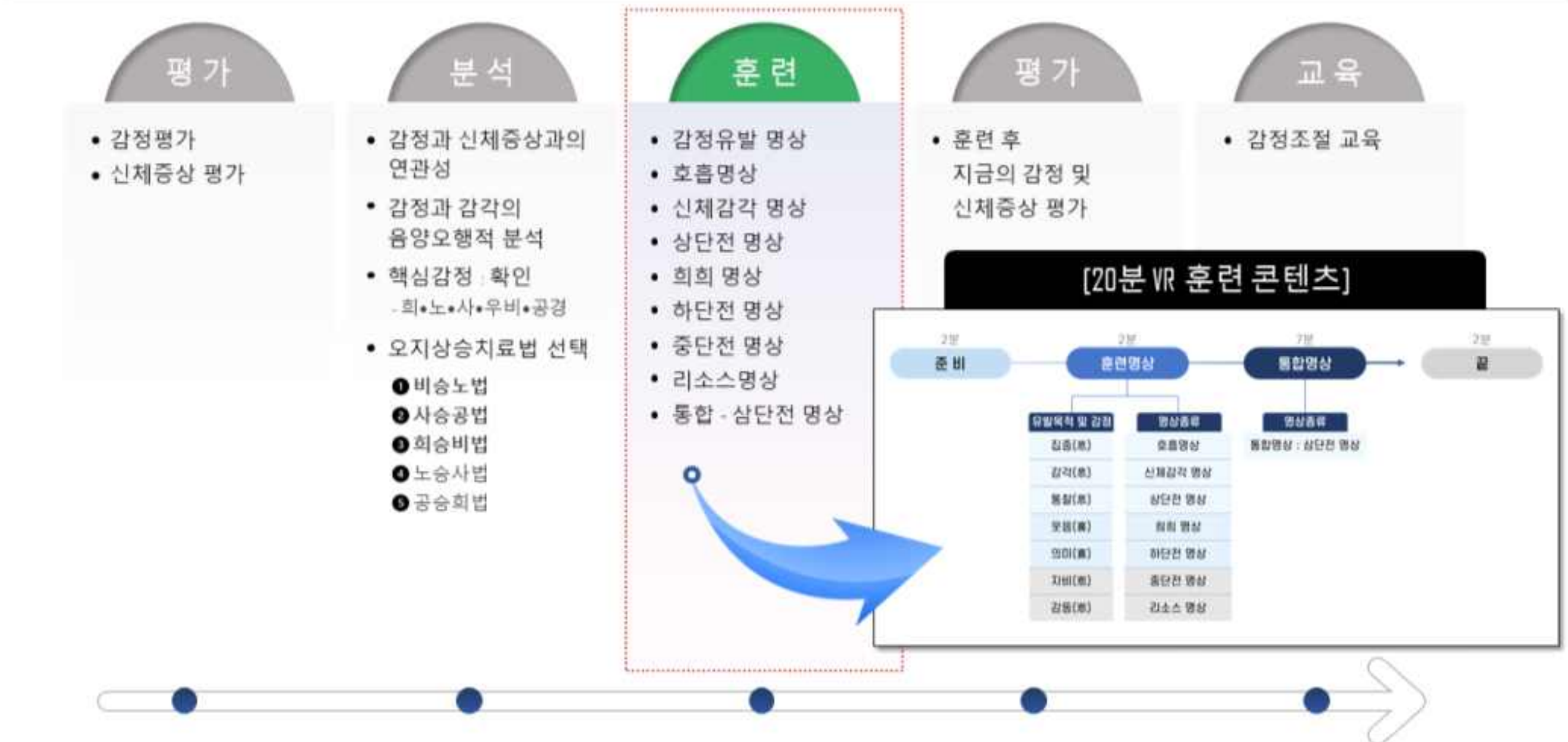

- VR 기반 오지상승위치료법은 기존 오지상승위치료법과 동일하게 평가, 분석, 훈련, 재평가, 교육의 단계로 이루어진다. (총 40분)
  (1) 평가 : 환자의 정서 및 감정 평가, 신체증상 평가 (CSEI-S, MRM, SUDS 평가) (5분)
  (2) 분석 : 감정과 신체증상과의 연관성, 감정과 감각의 음양오행적 분석, 핵심감정 확인, 오지상승치료법 선택 (5분)
  (3) 훈련(VR) : 감정유발 명상, 호흡명상, 신체감각 명상, 상단전 명상, 희희명상, 하단전 명상, 중단전 명상, 리소스 명상, 통합-삼단전 명상 (20분)
  (4) 평가 : 훈련 후 현재 감정 및 신체증상 평가 (CSEI-S, MRM, SUDS 평가) (5분)
  (5) 감정조절 교육 (5분)
- 이 중 훈련 단계를 VR을 통해 시행하며 PTSD환자 대상으로는 오지상승위치료법 중 사승공(思勝恐)

에 해당하는 상단전 명상의 훈련을 위주로 시행한다. 상단전 명상 외에도 환자의 정서상태에 따라 다른 훈련 명상을 시행할 수 있다. (약 20분)

7. 관찰항목, 관찰검사 방법 및 일정

7.1 진행일정표

<표 2> 진행일정표

관찰시기 / 검사항목		screening	시험군: VR 기반 오지상승치료법 혹은 오지상승치료법 시행					Follow up
week		-1	0	1~4	4	5~8	8	12
Visit[1]		스크리닝	1	2~7	8	9~15	16	17
대조군 Visit		스크리닝	1		2		3	4
동의서 취득		●						
SCID-5		●						
PC-PTSD-5		●						
인구학적 조사		●						
흉부단순촬영 검사		●						
심전도 검사		●						
임상검사실 검사[2]		●					●	
임신진단검사[3]		●					●	
활력징후 측정		●	●	●	●	●	●	●
병력 및 치료력 조사		●	●	●	●	●	●	●
선정/제외기준 판정		●						
무작위배정			●					
신장/체중 측정[4]			●				●	●
병용약물 확인			●	●	●	●	●	●
이상반응 확인			●	●	●	●	●	●
기초설문	경계정충 변증도구		●					
증상평가 설문	PCL-5-K		●				●	●
	CGI-I		●				●	●
	K-PTGI		●				●	●
	FFMQ-s		●				●	●
	PHQ-15		●				●	●
	HADS		●				●	●
	ISI-K		●				●	●
	화병척도		●				●	●
경제성평가	EQ-5D		●		4주차설문		●	●
	EQ-VAS		●		4주차설문		●	●
	비용측정[5]		●		4주차설문		●	●
전후평가 (대조군 Visit2제외)	MRM		●		●		●	●
	CSEI-S		●		●		●	●
	SUDS		●		●		●	●
기기검사	HRV		●				●	
	QEEG		●				●	
순응도 확인			●		●	●	●	
방문일정교육			●		●	●	●	

1) Screening 검사는 방문1의 7일전 이내에 행해져야 하며 임상검사실 검사결과를 방문1(0주)에 확인할 수 있어야 한다. 방문1은 Baseline 방문이다.

2) 임상병리검사, 갑상선기능검사: 임상시험 대상자는 채혈하기 전 2시간 금식상태로 내원하여 다음의 항목을 검사한다. 방문1 기준 14일 이내의 검사결과가 있다면 적용 가능하며, 시험자의 판단에 따라 비정상적인 결과에 대한 재검사를 시행할 수 있다.

- 혈액학적 검사 : WBC, RBC, Hemoglobin, hematocrit, Platelet, MCV, MCH, MCHC, Diff count(Neutrophil, Lymphocyte, Monocyte, Eosinophil, Basophil)
- 혈액화학적 검사 : Total Bilirubin, ALP, AST, ALT, γ-GTP, Creatinine, Glucose, globulin, Total Protein, Albumin, BUN, Total cholesterol, Triglyceride, LDL cholesterol, HDL cholesterol, Na, K, Cl, T3, free T4, TSH
- 소변검사 : Specific gravity, pH, Leuko esterase, Nitrite, Protein, Glucose, Ketone, Urobilinogen, Bilirubin, Erythrocyte, 현미경검사(RBC, WBC, Epi.cell, crystal. cast, Other)

PCL-5-K: The Posttraumatic Stress Disorder Checklist for DSM-5<br>K-PTGI; Korea- Posttraumatic Growth Inventory-Short Form<br>CGI-I; The Clinical Global Impression-Improvement scale<br>FFMQ-S; Five Facet Mindfulness Questionnaire Short Form<br>PHQ-15; Patient Health Questionnaire-15<br>HADS; Hospital Anxiety and Depression Scale	ISI-K; Korean version of Insomnia severity index<br>CSEI-S; The Core Seven-Emotions Inventory Short Form<br>SUDS; Subjective Units of Distress Scale<br>MRM; Mentalizing the Rooms of Mind<br>EQ-5D; EuroQoL-5Dimension<br>EQ-VAS; EuroQoL-visual analog scale<br>HRV; Heart Rate Variation<br>QEEG; Quantative Encephalogram

7.2 관찰항목별 방법

7.2.1 피험자 동의서 서명 및 스크리닝 번호 부여

- 본 임상시험을 실시하기에 앞서, 시험자는 '피험자 동의를 얻기 위한 설명서'에 관한 내용을 피험자 본인에게 설명하고, 피험자가 내용을 잘 이해한 것을 확인한 다음, 본인의 자유의사에 따른 임상시험 참가 동의를 문서로 받는다.
- 동의를 받는 순서에 따라 다음의 스크리닝 번호를 부여한다. 피험자 스크리닝 번호는 다음 방법에 따라 기록한다.

• 실시기관 코드 : 원광대학교 산본병원(WS)
• 실시기관 코드-실시년도-Screening의 첫 글자-등록된 순서
  (ex : WS-22-S-015: 원광대학교 산본병원 2022년도 첫번째 임상시험에서 15번째 스크리닝 환자)

7.2.2 인구학적 조사 및 병력 조사

- 임상시험에 들어가기 전에 피험자의 인구학적 조사, KCD 질병코드, 병력 및 약물 투여력 등에 대하여 면담, 질문 등을 통하여 점검 결과를 증례기록지에 기록한다.

7.2.3 피험자 적합성 평가

- 인구학적 조사, 병력 조사, 문진 등을 통하여 피험자 선정 및 제외기준에 적합한지 평가한다.

7.2.4. 피험자 식별코드 부여

- 임상시험 참여에 동의하고, 피험자 적합성 평가를 통하여 피험자 선정 및 제외기준에 적합한자에 한하여 피험자 식별코드를 부여한다.
- 피험자 식별코드는 다음 방법에 따라 기록한다.

• 실시기관 코드 : 원광대학교 산본병원(WS)

7.2.5 임상검사항목

1) 임상병리 및 진단검사실 검사

---

3) 폐경이 되지 않은 여성 대상자에 한해서 Urine HCG를 시행하며 결과는 음성이어야 한다. 단, menstruation에는 visit1에 시행한다.
4) 신장은 방문1에서만 측정하며, 체중은 방문1, 16, 17에 측정하여 기록한다. 신장은 0.1cm, 체중은 0.1kg 단위까지 반올림하여 측정한다.
5) 비용측정은 직접의료비, 직접비의료비, 간접비의 조사를 위하여 별도 개발된 비용 조사지를 사용하여 조사한다.

(1) 임상병리검사

- 혈액학적 검사

: WBC, RBC, Hemoglobin, hematocrit, Platelet, MCV, MCH, MCHC, Diff count(Neutrophil, Lymphocyte, Monocyte, Eosinophil, Basophil), ESR

- 혈액화학적 검사

: Total Bilirubin, ALP, AST, ALT, γ-GTP, Creatinine, Glucose, globulin, Total Protein, Albumin, BUN, Total cholesterol, Triglyceride, LDL cholesterol, HDL cholesterol, Na, K, Cl, T3, free T4, TSH

- 소변검사 : Specific gravity, pH, Leuko esterase, Nitrite, Protein, Glucose, Ketone, Urobilinogen, Bilirubin, Erythrocyte, 현미경검사(RBC, WBC, Epi.cell, crystal. cast, Other)

(2) 임신반응 검사

- 가임기여성 피험자의 임신여부를 알아보기 위해 HCG 검사를 실시한다.
- 폐경이 되지 않은 여성 대상자에 한해서 시행하며 결과는 음성이어야 한다.
  단, menstruation에는 visit1에 시행한다.

(3) 흉부단순촬영 및 심전도 검사

- Chest PA, EKG를 실시한다.

(4) 활력징후 및 신장, 체중 측정

- 매 방문시마다 활력징후를 측정하며 신장은 visit1에, 체중은 visit1, 16, 17에 측정하여 기록한다.
- 신장은 0.1cm, 체중은 0.1kg 단위까지 반올림하여 측정한다.

2) 설문지 및 평가항목

(1) 경계정충 변증도구

- 박 등[42]에 의해 개발된 경계정충 변증도구를 이용하여 대상자의 변증유형을 분석한다.

(2) 외상후 스트레스 장애 척도 한국판(Korean version of PTSD Checklist-5:이하 PCL-5-K)

- DSM-5의 PTSD 증상을 측정하기 위해 Weathers 등[43]이 개발한 20문항의 자기보고 설문지이다. PCL-5는 PTSD의 개인선별, 진단 평가에 도움이 되며 PTSD 증상변화의 관찰목적에 적합한 검사이다. 지난 한 달 동안 과거 스트레스 경험이 일으킨 증상으로 인해 얼마나 고통스러웠는지를 0점부터 4점의 리커트 척도로 평정하며 총점은 0~80점 범위에 있다. PTSD 진단기준의 B항목에서 1개 이상(질문 1~5), C항목에서 1개 이상(질문 6~7), D항목에서 2개 이상(질문 8~14), E항목에서 2개 이상(질문 15~20)의 증상이 있는 경우 PTSD일 가능성이 커지며 20문항의 총점으로 증상의 심각도를 동시에 평가한 다. 김 등[44]이 저작권자인 미국 국립 PTSD 센터로부터 사용허가를 받은 후 한글로 번안하였다(Korean version of PCL-5, PCL-5-K).

(3) The Clinical Global Impression - Improvement scale(이하 CGI-I)[45][46][47]

- 이 척도는 환자에게 치료적 개입을 시작하기 전과 비교했을 때, 치료적 개입 이후에 환자의 정신적 장애가 호전되었는지 또는 악화 되었는지를 평가하여 1~7점의 점수를 매기는 방식으로 사용한다.

척도	변화정도	채점 가이드라인
1	매우 많이 호전됨<br>(Very much improved)	• 거의 모든 것이 좋다(nearly all better)<br>• 좋은 수준의 기능(good level of functioning)<br>• 최소한의 증상(minimal symptoms)<br>• 매우 실질적인 변화를 나타낸다(represents a very substantial change)
2	많이 호전됨<br>(Much improved)	• 유의하게 증상이 현저히 감소하면 더 좋다(notably better with significant reduction of symptoms)<br>• 기능 수준은 더 좋아졌지만, 일부 증상은 남아있다(increase in th level of functioning but some symptoms remain)
3	약간 호전됨<br>(Minimally improved)	• 임상적으로 의미있는 증상 감소가 거의 없거나 전혀 없는 상태에서 약간 더 좋다(slightly better with little or no clinically meaningful reduction of symptoms)<br>• 기본 임상 상태, 치료 수준 또는 기능적 능력에 거의 변화가 없음을 나타낸다(Represents very little change in basic clinical status, level of care, or functional capacity)
4	변화가 없음<br>(No change)	• 증상은 본질적으로 변함이 없다(symptoms remain essentially unchanged)
5	약간 악화됨<br>(Minimally worse)	• 약간 악화되었지만 임상적으로 의미가 없는 정도(slightly worse but may not be clinically meaningful)<br>• 기본 임상 상태 또는 기능적 능력에 약간의 변화가 있을 수 있다 (may represent ver little change in basic clinical status or functional capacity)
6	많이 악화됨<br>(Much worse)	• 임상적으로 유의한 증상의 증가와 기능 저하(clinically significant increase in symptoms and diminished functioning)
7	매우 많이 악화됨<br>(Very much worse)	• 심각한 증상 악화 및 기능 상실(severe exacerbation of symptoms and loss of functioning)

(4) 한국판 외상 후 성장 척도 (Korean version of Posttraumatic Growth Inventory:이하 K-PTGI)

- 외상을 경험한 이후에 이전의 기능 수준을 넘어서는 긍정적인 변화를 가리키는 외상 후 성장을 측정하기 위해 Tedeschi와 Calhoun이 개발한 척도로[48], 본 연구에서는 국내에서 송승훈 등이 타당화한 한국판 외상 후 성장척도를 활용한다[49]. 원 척도는 총 21문항이지만 한국판 척도는 타당화 과정에서 중복 문항 5개를 제거하여 16문항이며, 총 16문항의 내적 일치도(Cronbach's α)는 .95였다. 자기 지각의 변화, 대인관계의 깊이 증가, 새로운 가능성 발견, 영적·종교적 관심의 증가의 4개의 하위요인으로 구성되며 각 문항을 0점부터 5점까지 6점으로 평정하도록 되어있다.

(5) 5요인 마음챙김 단축형척도 (Five Facet Mindfulness Questionnaire Short Form:이하 FFMQ-S)

- 본 척도는 Baer외 (2004)[50]가 개발하고 국내에서 원두리, 김교헌(2006)[51]에 의해 타당화된 5요인 마음 챙김척도(FFMQ)을 바탕으로 간편하게 임상적으로 마음챙김의 전후를 비교 측정할 수 있는 척도로 구성한 강형원외(2017)[52]이 개발한 총 15문항 7점 척도인 5요인 마음챙김 단축형 척도를 사용하였다. 강형원(2017)외에서 보고한 내적합치도는 비자동성 .89, 자각행위 .79, 기술 .86, 비판단 .73, 관찰 .89, 전체 .85로서 신뢰롭고 타당한 척도로 보고하고 있다.

5요인 마음챙김 단축형 척도 신뢰도

하위 요인	문항 번호	문항 수	Cronbach' alpha
비자동성	1,5,15	3	.888
자각행위	2,9,13	3	.787
기술	3,6,11	3	.856
비판단	4,12,14	3	.725
관찰	7,8,10	3	.891
	총 문항	15	.854

(6) 신체화 증상 척도(Patient Health Questionnaire-15:이하 PHQ-15)

- 주로 정신적인 영향으로 나타나는 비특이적인 신체증상을 평가하기 위하여 사용된다. 위통, 허리 통증, 관절 통증, 생리통, 두통, 흉통, 어지러움, 기절할 것 같음, 심장이 빨리 뜀, 숨이 참, 성교 통증, 변비 또는 설사, 메슥거림, 피로감, 불면의 15문항으로 구성되어 있으며 각각 0-2점 척도로 총점의 범위는 0-30점이다[53][54].

(7) 병원 불안-우울 척도(Hospital Anxiety and Depression Scale:이하 HADS)

- 불안과 우울을 짧은 시간에 측정할 수 있도록 Zigmond 등[55]이 고안한 척도로 본 연구에서는 Oh 등[56]이 한국어로 번안한 도구 14문항을 사용하였다. 홀수 번호 7개는 불안에 관한 하부척도이며 짝수 번호 7개는 우울에 관한 하부척도로 각각의 문항은 4점 척도(0-3점)로 구성되어 있으며 점수가 높을수록 불안과 우울이 높음을 의미한다. 간단히 시행할 수 있는 선별검사로 환자가 정신과적 개입을 필요로 하는 불안이나 우울 증상을 가지고 있는지 쉽게 알 수 있다.

(8) 불면증 평가 도구(Korean version of Insomnia severity index:이하 ISI-K)

- ISI는 불면증에 대한 평가도구로서 본 연구에서는 Bastien 등이 개발하고[57], Cho 등이 타당화한 한국판 불면증 심각도 척도(ISI-K)를 사용하였다[58]. 총 7가지 항목으로 되어있는데, 1)입면장애의 정도, 2)수면유지 장애의 정도, 3)쉽게깨는 정도, 4)수면에 대한 만족도, 5)수면장애가 일상생활을 방해하는 정도, 4)수면장애로 인한 삶의 질손상정도, 7)불면증에 관한 걱정정도이다. 각 항목마다 0점부터 4점까지 점수를 매기며 총 28점으로 0-7점은 정상, 8-14점은 경계성, 15-21점은 임상적 불면정(중등도), 22-28점은 임상적 불면증(중증)으로 나눌수 있다[59]. Bastien 등의 연구에서 내적일치도 계수(Cronbach's α)는 0.74이며[57], Cho 등의 타당화 연구에서 내적일치도 계수(Cronbach's α)는 0.92로 나타났다[58].

(9) 화병척도

- 화병 환자의 심리적, 성격적 상태를 평가하기 위하여 권정혜 등[60]이 개발하였으며, 화병에 대한 자기보고식 척도로 화병의 정서 증상과 신체 증상을 반영하는 15개 문항(증상척도, 0~60점)과 화병과 관련된 지속적인 성격 특성을 반영하는 16개 문항(성격척도, 0~60점)으로 이루어져 있다. 점수가 높을 수록 증상이 심한 것을 의미한다. 0-'전혀 그렇지 않다', 1-'그렇지 않은 편이다', 2-'중간정도 그렇다', 3-'상당히 그렇다', 4-'완전히 그렇다'로 평가한다.

(10) 건강관련 삶의 질 평가 척도 (EuroQoL-5 Dimension:이하 EQ-5D)

- EQ-5D는 1987년에 설치된 EuroQol 그룹에 의해 설치되고 연속적으로 개발되었다. Korean EuroQol-5 dimension(KEQ-5D)는 건강관련 삶의 질을 효용(utility)으로 측정하는 도구로 다차원적 선호도 근거 건강관련 삶의 질 측정도구(multidimensional preference based HRQOL measure)이다. 최근 EuroQol Group은 EQ-5D-3L의 단점을 보완하기 위해 EQ-5D-3L의 각 영역별 domain을 3개에서 5개로 널린 새로운 삶의 질 측정 도구를 발표하였다. EQ-5D-3L은 총 243개의 건강상태를 표현할 수 있었으나 EQ-5D-5L은 3,125(=55)개의 건강상태를 표현하기 되면서 EQ-5D-3L의 천장효과를 줄이고 신뢰도와 민감도를 증가시키면서 기술적 풍부함 또한 향상시킬 수 있을 것으로 기대된다. 실제로 국내 1개 병원의 암환자를 대상으로 EQ-5D-3L과 EQ-5D-5L 두 가지 도구를 비교한 연구에서 EQ-5D-5L은 EQ-5D-3L에 비하여 천장효과가 적고 타당도가 높았으며 검사-재검사 신뢰도는 유사하다는 연구 결과를 보고하였다(Seon Ha Kim.2012)[61]. 현재 EuroQol Group에 의해 EQ-5D-5L의 한국어판 공식 버전이 제공되고 있다.

(11) 전반적 건강상태 (EuroQoL-Visual analogue scale:이하 EQ-VAS) 점수[62]

- EQ-VAS는 등급척도(rating scale) 중 하나로 20cm의 수직선 모양으로 나타낸 시각아날로그 척도이다. 상상할 수 있는 최고의 건강상태를 100점으로, 상상할 수 있는 최저의 건강상태를 0점으로 부여하고 주어진 건강상태에 대해 0점과 100점 사이의 점수를 부여하게 된다. 등급 척도로 얻은 점수는 건강결과의 순서를 분명하게 알려주며, 선호도에 대한 강도의 정보도 알려준다. 그러나 등급척도는 측정 비뚤림이 생기기 쉽다. 등급척도는 대체로 다른 도구의 보조적 사용이 권장되며(한국보건의료연구원, 2013), EQ-VAS 역시 EQ-5D-5L 및 EQ-5D-3L 표준검사지에 보조적으로 포함되어 있다.

(12) 비용측정

- 시험군과 대조군의 효과가 비용대비 유의한 차이가 있는지를 평가하기 위하여 직접의료비용, 직접비의료비용, 생산성손실비용을 조사한다.

(13) 마음의 방 그리기(Mentalizing the Rooms of Mind:이하 MRM)

- 마음의 방 그리기는 지금 이 순간 자기 자신의 힘의 근원이자 고통의 근원인 마음의 방들을 구조화, 시각화, 구체화하여 자신의 마음을 보다 더 객관적으로 관찰할 수 있도록 돕는 M&L 심리치료 기법 중의 하나이다[63]. 내담자로서, 치료자로서 자세와 반응 그리고 치료적 관계성을 확립할 수 있고, 막연한 마음의 시각화, 구체화에 도움이 되며 객관적인 경과 관찰의 유용한 도구로 임상적 가치가 높은 것으로 알려져 있다[64][65]. A4 용지와 연필을 앞에 놓고, 다음과 같은 지시문을 제시하며 진행한다.

지금부터는 **씨의 마음이 동그란 원이라고 표현해보겠습니다.
**씨의 마음 안에는 크고 작은 방들이 여럿있습니다.
마음안에 하나의 방만 있는 것이 아니고 여러개의 방들로 이뤄져 있습니다.
큰방, 작은방, 세모난방, 메모난방, 일그러진 방, 별방 혹은 이름 없는 방도 있을 수 있습니다.
혹은 우울의 방, 불안의 방, 분노의 방, 감사의 방... 등이 있을 수도 있습니다.
아래에 그려진 원을 내 마음이라고 생각하고,
지금 이 순간, **씨의 마음을 차지하는 방은 어떤 것이 있는지
하나 하나의 방에 이름을 붙여보겠어요?

이방들 중에서 어느 방이 가장 크게 차지하고 있습니까?
힘든 방도 다른 방도 모두 나름의 역사가 있고 하고 싶은 말도 많이 있겠지요.

관찰자의 눈으로 이방 저방 하나 하나 쳐다보는 상태, 이 방에 들어가 이방에서 빠져있는 것이 아니고 한발 떨어져 있는 그대로 보는 상태. 이것을 명상상태라고 말합니다.

---

(14) 핵심칠정척도 단축형 (The Core Seven-Emotions Inventory Short Form: 이하 CSEI-S)[66] [별첨 4-10호]

- 28문항으로 구성되어 희(喜) 4문항, 노(怒) 4문항, 사(思) 4문항, 우(憂) 4문항, 비(悲) 4문항, 공(恐) 4문항, 경(驚) 4문항이며, 각 문항별로 '전혀 그렇지 않다=1, 약간 그렇다=2, 웬만큼 그렇다=3, 꽤 그렇다=4, 매우 그렇다=5점'의 5점 리커트(Likert) 척도로 구성되어 있다.
- 본 척도는 T점수(평균 50점, 표준편차 10점)를 기준으로 하여 희(喜)를 제외한 노(怒), 사(思), 우(憂), 비(悲), 공(恐), 경(驚)은 감정별로 점수가 높을수록 고위험군에 속한다. 구체적인 절단점은 T점수 55~60점 이하는 주의군, 61~65점은 위험군, 66점 이상은 고위험군이다. 반면에 희(喜)의 경우는 낮을수록 위험군으로서 40~45점 이하는 주의군, 35~39점 이하는 위험군, 34점

이하는 고위험군이다..

- 특히 비(悲), 우(憂), 노(怒)의 정서가 높은 경우 (질병이 있는 사람들이 없다는 사람보다 유의미하게 높았던 정서)에는 더욱 주의를 요하는 것으로 평가한다. 핵심감정평가 단축형 하위요인과 전체 신뢰도는 다음과 같다.

<표 5> CSEI-S의 신뢰도

하위 요인	문항 번호	문항 수	Cronbach' alpha
희(喜)	1, 8, 15, 22	4	.814
노(怒)	2, 9, 16, 23	4	.851
사(思)	3, 10, 17, 24	4	.818
우(憂)	4, 11, 18, 25	4	.771
비(悲)	5, 12, 19, 26	4	.898
공(恐)	6, 13, 20, 27	4	.731
경(驚)	7, 14, 21, 28	4	.931
Total		28	.860

(15) 주관적 고통감척도(Subjective Units of Disturbance Scale:이하 SUDS)

- 주관적 고통감 척도는 주관적 고통지수(Subjective Units of Disturbance Scale)이라고 알려진 척도로서 1950년대 정신과 전문의 조셉울프가 환자의 고통이나 불편함을 느끼는 정도를 측정하기 위해 사용한 것을 0~10점으로 시각화 하여 사용한 것이다[67].
- 구체적으로 주관적 고통지수는 “0”은 고통이 전혀 없는 상태, 기분이 편안하며 완전히 긴장이 풀린상태를 의미하며, “10”은 불안이 더 이상 상상할 수 없을 정도로 최고조에 달한 최악의 상태, 공황상태에 빠져 압도될 것 같은 상태를 의미한다.

(16) 심박변이도(Heart Rate Variation:이하 HRV) 측정

- HRV는 항상성을 유지하기 위해 심장박동이 자율신경의 조절을 통해 지속적으로 변화하는 것을 분석하여, 교감 및 부교감신경 간의 균형 상태와 각각의 활성도를 측정하는 것이다[68].
- 편안하고, 조용하고, 시원한 방안에서 약간 누운 자세(semirecumbent)로, 안정을 취한 상태에서 눈뜬 상태로 측정한다. 측정 중 움직이거나 말을 하지 않고 정상적으로 호흡하게 한다. 디스크 전극을 양쪽 손목과 왼쪽 발목에 부착하여 3분 동안 측정한다.
- 측정기계인 SA-3000P에서 산출한 결과 값을 확인하며 시간영역 분석에서는 Mean HR, SDNN, 주파수 영역분석에서는 TP, LF, HF, LF/HF 수치를 측정한다.

(17) 정량화 뇌파(Quantative Encephalogram:이하 QEEG) 측정

- 편안하고, 조용하고, 시원한 방안에서 약간 누운 자세(semirecumbent)로, 최대한 깨어있는 상태에서 눈 감은 상태와 눈 뜬 상태 각각 5분 동안 측정한다.
- International 10/20 system에 의거하여 위치한 21개의 표면 electrode가 있는 electrodecap(Electro-cap; Mitsar, Russia)을 부착시킨다. 모든 electrode의 impedance는 5kΩ이하로 함. reference electrode는 Fz와 Cz사이에 위치하고, 증폭기로는 Mitsar-EEG 201 machine(Mitsar, Russia)을 사용한다. 모든 신호는 high frequency filter는 50Hz, low frequency filter는 0.3Hz, Notch filter는 55~65Hz로 하여 추출된다.
- 측정자는 피험자의 눈 움직임, 근육 움직임 또는 다른 artifact를 유심히 살피면서 EEG를 녹화한다. 측정 가장 앞, 가장 뒤쪽의 각각 500ms는 제거하고, WinEEG(http://www.mitsar-medical.com)의 automatic algorithms과 육안검사를 통해 artifact를 제거한다.

- Artifact를 제거한 EEG segments가 1분 이상이며, 반분신뢰도(split-half reliability)가 95%이상인 기록만 spectral 분석과 coherence, LORETA 분석을 WinEEG를 이용하여 실시한다. Spectral 분석은 linked ears reference montage와 local average(Laplacian) montage를 사용한다. linked ears reference montage는 일반적인 기준으로 사용되고, local average(Laplacian) montage는 국소적인 부위를 강조할 수 있고, 약물에 의한 뇌의 전반적인 영향을 최소화 시킬 수 있다는 장점(highlight local fields)이 있다. Fast Fourier transform으로 absolute power와 relative power를 각각 delta(0.5~4Hz), theta(4~8Hz), alpha(8~12Hz), beta(12~20Hz)로 계산한다.

(18) 구조화된 임상 면담 도구(Structured Clinical Interview for DSM-5: 이하 SCID-5)

- 본 연구에서 사용한 SCID는 Structured Clinical Interview for DSM-5 Axis I disorder(SCID)중 연구용판이다. 연구용 SCID는 임상용에 비해 질환 아형, 심한 정도(severity)와 경과 세부 진단(course specifiers),과거 기분 삽화에 대해 더 자세하게 기록할 수 있게 되어 있다. SCID는 18세 이상의 성인에 사용되도록 제작되었지만 약간의 수정을 가하면 청소년에게도 사용할 수 있다. 그러나 인지적 손상이 심하거나 불안, 초조, 혹은 심각한 정신증적 증상이 있는 사람들은 SCID를 이용한 면담이 어려울 수 있다. SCID를 이용할 것인가의 여부는 면담의 시작 부분인 개괄 영역에서 판단하도록 되어 있다. 이외에도 개괄 부분에서 인구학적 자료, 교육력, 직업력, 치료 상태에 대한 정보를 수집할 수 있다.

(19) PC-PTSD-5(The Primary Care PTSD Screen for DSM-5

- PC-PTSD-5는 DSM-5 진단 규준에 맞추어 일차의료기관에서 PTSD를 선별하기 위한 목적으로 개발되었다[69]. 지난 1개월 전에 재경험, 회피, 마비, 과각성을 측정하는 네 개의 문항에 정서에 대한 부정적 변화의 문항이 더해져 다섯 개의 문항에 '예', '아니오'로 응답하게 되어있다. 본 연구에서는 스크리닝 단계에서 박 등(2016)[70]이 번안한 척도를 사용하여 절단점인 3점 이상인 대상자를 연구에 포함한다.

7.2.6 순응도 평가

-중재에 대한 순응도를 아래와 같이 평가하도록 한다.

-시험 참여기간 동안의 전체 순응도는 75% 이상이어야 하며, 75% 미만인 경우 해당 시험대상자는 순응도가 나쁜 것으로 간주하여 PP분석군에서 제외한다.

$$순응도 = \frac{실제\ 실수된\ 중재횟수}{계획된\ 중재횟수} \times 100$$

7.2.7 이상반응 조사

-시험자는 임상시험 참여 후 나타나는 이상반응 여부를 시험 종료 직전 피험자에 대한 진찰로서 관찰하여 시험 치료법과의 인과관계에 대하여 '이상반응 기록표'에 기록한다.

7.3 관찰검사 방법 및 일정

7.3.1 선정방문과 스크리닝

- 내원 혹은 전화를 통하여 임상시험 참여를 신청한 지원자를 다음 순서에 따라 스크리닝을 한다.

1) 지원자가 서명한 피험자 동의서를 얻는다. 이 시험에 참여하는 지원자는 피험자 동의를 얻기 위한 설명을 거쳐 자유의사에 의해 피험자 동의서에 서면 동의를 한 후 시험에 참여시킨다.
2) **임상시험 대상자로서의 적합성을 파악하기 위해** 아래와 같은 검사를 시행한다.
   - 인구학적 정보: 피험자 성명, 성별, 생년월일, 주소, 휴대전화 번호
   - SCID-5(Structured Clinical Interview for DSM-5)에 의해 PTSD로 진단받은 자
   - PC-PTSD-5 평가시 3점 이상인 자
   - 문진 및 병력 조사: 현병력, 과거력, 흡연 여부, 음주 여부, 치료력, 약물 복용
   - 이학적소견: 혈압, 맥박, 체온, 호흡, 체중, 신장
   - 흉부단순촬영, 심전도검사
   - 임상병리 검사, 임신반응검사
3) 스크리닝을 통하여 선정, 제외기준 적합 여부를 평가하여 시험 참여가 적합한 경우에 무작위배정에 따른 피험자 식별코드를 부여한 후 증례기록지에 기록한다.

7.3.2 시험시행

- 무작위 배정에 맞게 시험군1, 시험군2, 대조군으로 나누어 진행을 하게 된다.

가. 대조군으로 배정된 경우 다음과 같이 시행된다.

-처음(baseline) ①기초설문(경계정충변증도구) ②증상평가설문(PCL-5-K, CGI-I, K-PTGI, FFMQ-S, PHQ-15, HADS, ISI-K 화병척도), ③경제성평가(EQ-5D, EQ-VAS, 비용측정), ④전후평가(MRM, CSEI-S, SUDS), ⑤기기검사(HRV, QEEG) 측정을 실시한다.

-8주간 평소 일상생활을 하도록 하고, 임상시험 연구담당자와 최소한의 접촉을 위해 2주에 한 번씩 전화로 근황을 확인한다.

-4주 후(시험군 8회기 후) 경제성평가(EQ-5D, EQ-VAS, 비용측정)를 실시한다.

-8주 후(시험군 16회 최종 종결 후)에 ①증상평가설문(PCL-5-K, CGI-I, K-PTGI, FFMQ-S, PHQ-15, HADS, ISI-K 화병척도), ②경제성평가(EQ-5D, EQ-VAS, 비용측정), ③전후평가(MRM, CSEI-S, SUDS), ④기기검사(HRV, QEEG)측정을 실시한다.

-12주 후 Follow up에는 시험군과 같이 대기대조군에게 ①증상평가설문(PCL-5-K, CGI-I, K-PTGI,

FFMQ-S, PHQ-15, HADS, ISI-K 화병척도), ②경제성평가(EQ-5D, EQ-VAS, 비용측정), ③전후평가(MRM, CSEI-S, SUDS)측정을 실시한다. 이후 모든 시험이 종결 후 원하는 경우 8주간 주 2회 8회차에 걸쳐 VR기반 오지상승위치료를 실시한다.

나. 시험군에 해당하는 경우 다음과 같은 임상시험이 진행된다.

- **시험군 1에는 VR기반** 오지상승위치료, **시험군 2는** 오지상승위치료를 실시한다.
- -처음(baseline) ①기초설문(경계정충변증도구) ②증상평가설문(PCL-5-K, CGI-I, K-PTGI, FFMQ-S, PHQ-15, HADS, ISI-K 화병척도), ③경제성평가(EQ-5D, EQ-VAS, 비용측정), ④전후평가(MRM, CSEI-S, SUDS),⑤기기검사(HRV, QEEG) 측정을 실시한다.
- -4주 후(시험군 8회기 후) 경제성평가(EQ-5D, EQ-VAS, 비용측정)를 실시한다.
- -8주 후(시험군 16회 최종 종결 후)에 ①증상평가설문(PCL-5-K, CGI-I, K-PTGI, FFMQ-S, PHQ-15, HADS, ISI-K 화병척도), ②경제성평가(EQ-5D, EQ-VAS, 비용측정), ③전후평가(MRM, CSEI-S, SUDS), ④기기검사(HRV, QEEG)측정을 실시한다.
- -12주 후 Follow up에는 ①증상평가설문(PCL-5-K, CGI-I, K-PTGI, FFMQ-S, PHQ-15, HADS, ISI-K 화병척도), ②경제성평가(EQ-5D, EQ-VAS, 비용측정), ③전후평가(MRM, CSEI-S, SUDS)측정을 실시한다. 이후 모든 시험이 종결 후 원하는 경우 8주간 주 2회 8회차에 걸쳐 VR기반 오지상승위치료를 실시한다.

-매회 차 훈련 전후에는 MRM, CSEI-S, SUDS를 실시하고, 이상반응은 매회 차 훈련 후 및 12주차 Follow up때 관찰한다.

7.3.2.1 1회차(개입시작)

-선정 방문 후 3일 이내(선정여부는 전화통보)에 이루어지고 다음의 평가가 실시된다.

① 흉부단순촬영검사, 심전도, 임상병리검사 등 선정 방문의 결과를 확인한다.
② 선정 방문 이후 피험자의 병력이나 치료약물의 변화여부를 확인하여 기록한다.
③ 혈압, 맥박, 체온, 호흡수 및 신장, 체중을 측정한다.
④ 개입 전, 기초설문(경계정충변증도구), 증상평가설문(PCL-5-K, CGI-I, K-PTGI, FFMQ-s, PHQ-15, HADS, ISI-K 화병척도), 경제성평가(EQ-5D, EQ-VAS, 비용측정), 전후평가(MRM, CSEI-S, SUDS), 기기검사(HRV, QEEG)를 측정하고 기록한다.
⑤ 시험군1은 VR기반 오지상승위치료를, 시험군2는 오지상승위치료를 40분간 시행한다.
⑥ 개입 전후로 MRM(다음의 방 그리기)와 CSEI-S, SUDS를 평가한다.
⑦ 다음 방문 일을 지정한다.

7.3.2.2 2-7회차(개입)

① 혈압, 맥박, 체온, 호흡수를 측정한다.
② 개입 전, MRM(마음의 방 그리기)와 CSEI-S, SUDS를 평가한다.
③ 시험군1은 VR기반 오지상승위치료를, 시험군2는 오지상승위치료를 40분간 시행한다.
④ 개입 후, MRM(마음의 방 그리기)와 CSEI-S, SUDS를 평가한다.
⑤ 병용약물과 이상반응여부 및 순응도를 확인한다.
⑥ 다음 방문 일을 지정한다.

7.3.2.3 8회차(개입)

① 혈압, 맥박, 체온, 호흡수를 측정한다.
② 개입 전, MRM(마음의 방 그리기)와 CSEI-S, SUDS를 평가한다.
③ 시험군1은 오지상승위치료를, 시험군2는 VR기반 오지상승위치료를 40분간 시행한다.
④ 개입 후, MRM(마음의 방 그리기)와 CSEI-S, SUDS 및 경제성평가(EQ-5D, EQ-VAS, 비용측정)를 시행한

다.
⑤ 병용약물과 이상반응여부 및 순응도를 확인한다.
⑥ 다음 방문 일을 지정한다.

7.3.2.4 9-15회차(개입)
① 혈압, 맥박, 체온, 호흡수를 측정한다.
② 개입 전, MRM(마음의 방 그리기)와 CSEI-S, SUDS를 평가한다.
③ 시험군1은 VR기반 오지상승위치료를, 시험군2는 오지상승위치료를 40분간 시행한다.
④ 개입 후, MRM(마음의 방 그리기)와 CSEI-S, SUDS를 평가한다.
⑤ 병용약물과 이상반응여부 및 순응도를 확인한다.
⑥ 다음 방문 일을 지정한다.

7.3.2.3 16회차(개입)
① 혈압, 맥박, 체온, 호흡수 및 체중을 측정한다.
② 개입 전, MRM(마음의 방 그리기)와 CSEI-S, SUDS를 평가한다.
③ 시험군1은 VR기반 오지상승위치료를, 시험군2는 오지상승위치료를 40분간 시행한다.
④ 개입 후, MRM(마음의 방 그리기)와 CSEI-S, SUDS 및 증상평가설문(PCL-5-K, CGI-I, K-PTGI, FFMQ-s, PHQ-15, HADS, ISI-K 화병척도), 경제성평가(EQ-5D, EQ-VAS, 비용측정), 기기검사(HRV, QEEG) 측정을 실시하고 기록한다.
⑤ 병용약물과 이상반응여부 및 순응도를 확인한다.
⑥ 다음 방문 일을 지정한다.

7.3.2.6 17회차(12주차 추적방문)
12주차 방문하여 다음과 같은 설문지 평가를 실시한다.
① 혈압, 맥박, 체온, 호흡수 및 체중을 측정한다.
② 개입종결 후 이상반응이나 특이사항을 확인하여 기록한다.
③ MRM(마음의 방 그리기)와 CSEI-S, SUDS 및 증상평가설문(PCL-5-K, CGI-I, K-PTGI, FFMQ-s, PHQ-15, HADS, ISI-K 화병척도), 경제성평가(EQ-5D, EQ-VAS, 비용측정)을 실시하고 평가한다.
④ 임상시험 종결한다.

7.3.2.7 추가 방문
- 추가방문은 예정된 방문 외에 시험대상자 요청 또는 시험자의 판단에 의해 필요하다고 판단될 때 수시로 이루어질 수 있다. 시험대상자가 예정되지 않은 날에 방문한 경우에는 이상반응, 병용약물, 시행한 검사의 결과 및 그에 따른 의학적 처치 등을 증례기록지에 기록하여야 한다. 중도 탈락된 시험대상자의 경우라도 추가방문을 실시하면 임상검사실검사, 임신진단검사, 유효성평가 검사를 시행할 수 있다.

8. 예측 부작용

- 본 VR기반 오지상승위치료법은 아래와 같은 부작용이 발생할 수 있으나, 미미하여 잠시 사용 중지 후 다시 사용하거나 상태 확인 후 재차 실시 여부 결정할 수 있다.
• 간의 두통/눈의 피로/눈 또는 근육경련/눈의 불수의 운동/메스꺼움/어지럼증/머리 또는 눈의 불쾌감 또는 통증/졸림/피로감/눈, 피부나 두피에 전염성 증상/피부 자극 같은 부작용 증세가 발생할 수도 있다.

9. 중지 및 탈락 기준

9.1 중지의 정의

- 부작용, 이상사례 발생 등으로 인하여 임상시험을 진행할 수 없거나 임상시험의 진행이 대상자의 안전보호를 위협하여 그 진행을 멈추는 것으로 정의한다.

9.2 중지의 기준

1) 이상반응이 발생한 경우
2) 기타 시험자의 판단에 의해 시험 진행이 적합하지 못하다고 판단되는 경우

9.3 탈락의 정의

- 계획서에 명시되어 있는 대로 훈련시행과 그 전후의 관찰과정을 완료한 경우 피험자가 시험을 완료한 것으로 정의한다. 이상반응 또는 다른 이유로 인해 시험을 완료하지 못한 경우 피험자가 탈락된 것으로 본다.

9.4 탈락의 기준

- 시험자는 치료와 관찰을 중단시키고 환자를 탈락시킬 수 있으며, 피험자는 언제나 시험에서 자유의사로 탈락할 수 있다.
- **탈락의 기준은 다음과 같다.**

1) 시험자의 지시에 불응하는 피험자의 경우
2) 계획서 위반
   피험자나 시험자가 계획서에 기록된 내용을 준수하지 않는 경우
3) 중대한 이상반응이 발생한 경우
4) 순응도 75%미만인 경우
5) 기타
   피험자가 동의를 철회하거나 치료의 중단을 요청한 경우

9.5 중지 및 탈락의 처리 과정

- 피험자가 탈락 및 중지한 날짜와 시간, 사유를 종료보고서에 기록한다. 어떠한 사유든지 탈락 및 중지한 피험자를 추적 관찰하기 위한 모든 노력을 기울여야 하며 안전성에 대한 추적관찰 평가를 치료 시행 후 1주일 이내에 수행하여야 한다. 피험자는 어떠한 사유든지 언제나 시험에서 자유의사로 탈락할 수 있으며 탈락 사유에 대하여 진술할 의무는 없다. 그러나 시험자는 탈락 및 중지한 사유 또는 사유 파악의 실패 등을 종료보고서에 기록하여야 하며 탈락한 피험자를 추적 관찰하는데 모든 노력을 기울여야 한다. 시험자는 피험자를 시험에서 제외시킬 수 있는 선택권 또한 가지고 있다.
- 임상시험 참여 중 발생한 부작용 등의 사유로 인해 연구 참여를 중단하는 경우에는 예측하지 못한 추가 비용의 발생이 가능하므로, 해당 비용 발생 항목 및 금액에 대해 수집하기 위하여 경제성평가 증례기록 설문을 실시할 수 있다.

9.6 순응도와 임상시험 계획서 위반에 대한 처리

- 임상시험 책임자와 담당자는 임상시험 계획서의 위반이 발생하지 않도록 계획서에 대해 충분히 숙지하고 철저히 이행한다. 시험담당자는 대상자의 방문, 검사 일정 준수를 위하여 적절한 조치, 예를 들어 휴대

폰 문자메세지, 전화 모니터링, 서면통보, 이메일 등을 실시한다. 부득이하게 발생된 계획서 위반 사항에 대해서는 다음에 따라 처리한다.

- 중대한 임상시험계획서 위반의 경우 예를 들어 선정/제외기준 위반, 동의서 미취득, 임상시험 결과에 영향을 미칠 수 있는 약물 등을 복용한 경우 등 시험대상자의 안전과 시험결과에 중대한 영향을 미칠 수 있는 위반의 경우, 해당 시험대상자의 정보는 분석에서 제외함을 원칙으로 한다.
- 기타 경미한 위반사항은 위반 또는 지연 정도와 사유를 정확히 기재하여 분석할 때 임상시험 결과에 영향을 주었는지 평가한다.

10. 평가기준, 평가방법

10.1. 평가기준

10.1.1 VR 기반 오지상승위요법 및 오지상승위요법 전후 평가 분석

- 주 유효성 설문지 평가분석을 실시한다.
- 부 유효성 설문지 평가분석을 실시한다.

10.1.2 정량화뇌파(QEEG) 분석

① Frequency spectral analysis:

- 각 주파수대별 absolute power (μV2), relative power(%)의 오지상승위요법 및 VR 기반 오지상승위요법 전후 변화를 비교한다.
- normative databases을 이용한 absolute power와 relative power의 z-score의 오지상승위요법 및 VR 기반 오지상승위요법 전후 변화를 비교한다.

② Source localization-LORETA(low resolution brain electromagnetic tomography):

- 주파수대별 LORETA(low resolution brain electromagnetic tomography)를 통하여 해당 주파수의 피질 부위의 current densities를 추정하여 활성화된 피질부위에 해당하는 브로드만 영역을 오지상승위요법 및 VR 기반 오지상승위요법 전후 변화를 비교한다.

11. 통계분석 방법

11.1. 인구학적 자료와 시험 시작 시 임상병력 자료의 분석

- 기술통계 분석

본 임상시험에 포함된 피험자의 인구학적(인구통계학적 특성) 자료 및 시험 시작 시 임상병력 자료(이하 기초자료라 함)들은 연속형 자료는 평균, 표준편차, 최소, 최대치 등을 제시하고 범주형 자료는 빈도표를 제시한다. 이를 위해 기술 통계(Descriptive Statistics)와 빈도 분석(Frequency Analysis)를 실시한다.

- 정규성 검증

각 측정 시점에서 관측된 평가변수들의 정규성을 검증한다.
가. 정규성을 만족한 경우 모수 검정 실시
나. 정규성을 만족하지 못한 경우 비모수 검정을 실시한다.

11.2. 평가변수의 분석방법

11.2.1. VR 기반 오지상승위요법 및 오지상승위요법의 효과성 분석

1. 차이검증

(1) 주 유효성 평가 측정 도구

① PCL-5-K에 대하여 연구대상자 시험군 1,2와 대조군의 중재 개입 전 후(0주차 1회기_8주차 16회기) 실시 및 12주차 방문 변수에 대하여 기술통계(평균, 표준편차, 중위값 등)분석을 실시한다.

② PCL-5-K 에 대하여 연구대상자 시험군1,2와 대조군의 기술통계값을 분석한 후 개입 전 후 측정값에 대하여 **정규성 검증을 만족할 경우, 모수 차이검증을, 만족하지 못할 경우 비모수 검증을** 실시한다.

가. 모수 검증

-각 군내 차이검증을 실시할 시 0주차 1회기와 8주차 16회기(개입전후) 대응표본 T-test를 실시한다

- 각 세군(가상현실기반 오지상승위개입군, 오지상승위개입군과 대조군)간 차이검증을 실시할 경우 모수검증법인 ANOVA(one-way analysis of variance)를 실시하고, 사후검증(Scheffe, 등분산이 가정되지 않을 경우 여Dunnett T3 혹은 Games-Howell)을 수행한다.

나. 비모수 검증

- 정규성을 만족하지 못할 시 각 군내 차이검증은 0주차 1회기와 8주차 16회기(개입 전 후)에 대하여 비모수 윌콕슨-맨위트만 순위 차이검증(Wilcoxon's Rank Sum Test, Mann-Whitney U Test) 차이검증을 실시한다.
- 정규성을 만족하지 못할 시 세 군 간(시험군 1_2, 대기대조군)의 비모수적 ANOVA인 Kruskal Wallis 검정을 실시하고, 자유도와 검정통계량(H) 및 유의확률을 보고한다. 더불어 비모수 사후 검증 실시는 Friedman을 통한 집단 간 순위분포를 분석한다.
- 이를 통해 군 간의 차이 및 변화를 분석하여, VR기반 오지상승위치료법 및 오지상승위치료법에 대한 효과성의 차이검증을 실시할 것이다.

(3) 부유효성 평가 측정 도구

① 부유효성 평가 측정 설문지(CGI-I, CSEI-S, K-PTGI, FFMQ-S, PHQ-15, HADS, ISI-K, 화병척도, 주관적 불편감) 및 기기검사(QEEG, HRV) 시행 결과 점수에 대해서 연구 대상자 시험군 1,2와 대조군에 대한 기술통계분석을 실시한다.

② 각 부유효성 평가 척도(CGI-I, CSEI-S, K-PTGI, FFMQ-S, PHQ-15, HADS, ISI-K, 화병척도, 주관적 불편감) 및 기기검사(QEEG, HRV) 시행 결과 점수에 대하여 전후 측정값에 대한 세군(VR오지상승위개입군, 오지상승위개입군과 대조군)의 군내 대응표본 T-test 및 군간 차이검증을 실시할 것이다. 이때, 군내 차이검증은 정규성 검증을 통하여 정규성을 만족할시 모수검증을, 정규성을 만족하지 못할시에는 비모수 검증을 통해 각각 대응표본 T-test를 실시한다.

가. 모수검증

- 각 군내 차이검증을 실시할 시 0주차 1회기와 8주차 16회기(개입전후) 각 부 유효성 평가 설문지에 대해 대응표본 T-test를 실시한다

- 각 세군(가상현실기반 오지상승위개입군, 오지상승개입군과 대조군)간 각 부 유효성 평가 설문지에 대해 차이검증을 실시할 경우 모수검증법인 ANOVA(one-way analysis of variance)를 실시하고, 사후검증(Scheffe, 등분산이 가정되지 않을 경우 Dunnett T3 혹은 Games-Howell)을 수행한다.

나. 비모수 검증

- 정규성을 만족하지 못할 시 각 부 유효성 평가 설문지에 대해 각 군내 차이검증은 0주차 1회기와 8주차 16회기(개입 전 후)에 대하여 비모수 윌콕슨-맨위트만 순위 차이검증(Wilcoxon's Rank Sum Test, Mann-Whitney U Test) 차이검증을 실시한다.
- 정규성을 만족하지 못할 시 각 부 유효성 평가 설문지에 대해 세 군 간(시험군 1_2, 대기대조군)의 비모수적 ANOVA인 Kruskal Wallis 검정을 실시하고, 자유도와 검정통계량(H) 및 유의확률을 보고한다. 더불어 비모수 사후 검증 실시는 Friedman을 통한 집단 간 순위분포를 분석한다.
- 이를 통해 군 간의 차이 및 변화를 분석하여, VR기반 오지상승위치료법 및, 오지상승 치료법에 대한 효과성의 차이검증을 실시할 것이다.

③ 정량화뇌파(QEEG)분석

가. Frequency spectral analysis:

-Baseline을 기준으로 하여, 전체 피험자의 시험군1,2와 대조군에 따른 각 electrode의 위치의 주파수 대별 absolute power (μV2), relative power(%)를 기술통계(평균, 표준편차) 분석을 실시한다.

나.Source localization-LORETA(low resolution brain electromagnetic tomography): 주파수대와 브로드만 영역을 구분하여 LORETA(low resolution brain electromagnetic tomography) 1회차, 16회차의 두뇌 활성화 영역의 차이검증을 $p<.05$의 수준에서 분석을 실시한다.

11.2.2. 오지상승위치료 또는 VR기반 오지상승위치료 전후 측정값들 간의 상관성 분석

정신요법 주 유효성 평가(PCL-5-K)척도와 부 유효성 평가척도의 정규성 검증이후 정규성을 만족하면 모수(pearson), 정규성을 만족하지 못 할 시에는 비모수 spearman 상관관계 분석을 실시함으로써 상관관계 분석을 실시한다.

11.2.3. 경제성 평가

- 경제성 평가 분석은 FAS 분석을 원칙으로 하며 결측 분석 결과에 대한 민감도를 확인하기 위한 PP 분석을 같이 시행한다. 경제성평가의 비용 및 효과(Utility) 추정은 임상시험의 효과추정과 동일한 방식으로 missing data에 대한 기전 등을 분석한 후 적합한 imputation 방식을 선택하여 시행한다.
- 임상시험과 동시에 진행하는 경제성평가는 임상연구 대상 치료와 기존치료(Usual care)간의 비용효과성을 확인하고자 시행한다. 주 경제성평가지표(Primary economic endpoint)는 EQ-5D로 산출된 Quality Adjusted Life Years(cost per QALY) gained이고, 이차평가지표는 EQ-VAS로 산출된 cost per EQ-VAS 등 효과성 평가지표들이다.
- 1차 분석기간은 8주(총 추적 기간)로 시행하며, 이후의 기간에 대한 추정이 필요할 경우 추적기간 이후의 비용과 효과를 회귀모형 등을 통하여 외삽(Extrapolation)하여 추정하거나 Decision modelling analysis 등을 시행하는 2차 분석을 시행한다.
- 임상시험과 관련하여 발생하는 치료비용은 치료 횟수와 단위비용을 결합하여 산출하며, 임상시험 기관 내에서 발생하는 치료비용은 맹검 해제 후 연구자가 전산 자료를 조사하여 기록한다. QALY 산출을 위한 삶의 질 추정은 EQ-5D로 도출된 삶의 질을 주 평가변수로 사용하고, 산출 방식은 Area under the curve method(Brazier J, Ratcliffe J, Salomon JA, Tsuchiya A: Measuring and Valuing Health Benefits for Economic Evaluation. Oxford, UK: Oxford University Press; 2007.)를 이용한다.

- 총 분석 기간(Time Horizon)을 임상시험 기간 이후에 대한 외삽을 할 경우 12개월 이상이 될 경우 비용 단위는 2021년 대한민국 화폐단위(원)으로 통일하고, 건강보험심사평가원 경제성평가 지침에 근거하여 5%의 할인율을 적용한다.
- 본 연구의 분석관점은 사회적 관점이며, Baseline analysis에서는 연구에서 사용되는 모수들(parameter)의 대표값(평균 등)을 사용하여 시행하고, 민감도분석은 가능한 모든 추정모수들의 분포와 대표값 들을 사용하여 확률적 민감도 분석(Probabilistic Sensitivity Analysis)을 시행한다.
- 분석 결과의 제시는 Incremental cost effectiveness ratio를 포함한 결과 분석 테이블들과 함께, 비모수적 방법으로 확인된 신뢰구간을 포함하는 Cost Effectiveness plane과, National threshold 의 변화에 따른 비용효과성의 민감도를 확인할 수 있는 Cost Effectiveness Acceptability Curve(CEAC), 대상 인구집단의 정보의 가치를 추정할 수 있는 Value of information analysis 결과 그래프 등을 함께 제시한다.
- 본 연구의 통계분석은 표본 수가 30 이하인 경우 모든 연속형 변수들의 정규성을 Shapiro-Wilk test 등을 통해 먼저 검증한 후 시행하며, 통계적 유의수준은 p-value 0.05로 검정하고, 분석 프로그램은 Stata 14 MP version과 R program(4.0.2 version)을 사용하여 시행하며, 모델링을 통한 외삽을 수행한 경우에는 Treeage Pro 2016을 사용하여 연구를 수행한다.

11.2.4. 정성적 평가

- VR오지상승위치료군, 오지상승위치료군과 대조군의 MRM에 대한 질적 평가를 실시한다.
- MRM은 투사적 검사의 일종으로서 객관적 평가와 다른 질적 내용을 분석한다.
- 투사적 심리검사를 평가할 수 있는 임상심리사 1인과 한방신경정신과 전문의 1인이 투사적 평가를 실시한다.

11.3. 안전성 변수의 분석방법

- 발현된 모든 이상반응은 자세한 설명과 함께 나열한다. VR기반 오지상승위치료법 또는 오지상승위치료법과 연관성이 있는 이상반응과 연관성이 없는 이상반응의 빈도를 기록한다.
- 이상 반응의 경우 이상반응의 빈도의 총합 혹은 각각의 빈도에 대해 McNemar 검정법을 사용하여 빈도에 유의미한 차이가 있는지 검증함. 이상반응의 강도(혹은 수준)를 조사한다면 paired t-test 혹은 윌콕슨 맨위트만 순위 차이검증(Wilcoxon's Rank Sum Test, Mann-Whitney U Test)을 실시한다.

12. 이상반응을 포함한 안전성의 평가기준, 평가방법 및 보고방법

- 피험자 안전성

본 연구는, 외상후 스트레스 장애 환자에 대한 가상현실 기반 오지상승위치료법의 안전성, 유효성 및 경제 성 평가하는 연구로서, 본 연구의 개입방법인 오지상승위치료법은 기존 진료에서 진행되고 있는 시술임. 가상현실 기반 오지상승위치료법은 기존 치료법 중 훈련 단계만을 가상현실 기기로 활용하는 것으로, 의학적으로 판단하여, 연구로 인한 추가적 위험이 기존 진료 과정에서 진행되고 있는 시술, 또는 치료 방법보다 현저하지 않으므로, 이 연구로 인해 연구대상자가 추가적으로 입게 되는 신체적, 정신적 위해 및 특이 손상 은 없을 것으로 예측됨. 또한 본 임상시험에 사용 기기는 비침습적이며 짧은 기간동안 임상시험에 참여하기 때문에 안전에 대한 위험은 없다 할 수 있음.

12.1 안정성 관련 용어의 정의

12.1.1 이상반응

- 이상반응(Adverse Event, AE)은 임상시험 중 피험자에게 발생하는 바람직하지 않고, 의도되지 않은 증후(sign), 증상(symptom), 질병을 말하며, 해당 임상시험에 사용된 치료법과 반드시 인과관계를 가져야 하는 것은 아니다.

12.1.2 중대한 이상반응

- 중대한 이상반응 (Serious AE)은 임상시험에 사용한 침치료로 인하여 발생되는 이상반응 중에서 다음 각목의 1에 해당하는 경우를 말한다.
  - ·사망을 초래하거나 생명을 위협하는 경우
  - ·입원 또는 입원 기간의 연장이 필요한 경우
  - ·지속적 또는 의미 있는 불구나 기능 저하를 초래하는 경우
  - ·선천적 기형 또는 이상을 초래하는 경우

12.2 이상반응의 평가

12.2.1 중증도 평가

- 이상반응이 발생하면 다음의 중증도(Severity) 평가기준에 의해 보고하여야 한다.
  - ·경증(mild): 피험자의 정상적인 일상생활(기능)을 저해하지 않고, 최소한의 불편을 야기하며 피험자가 쉽게 견딜 수 있는 경우
  - ·중등도(moderate): 피험자의 정상적인 일상생활(기능)을 유의하게 저해하는 불편을 야기하는 경우
  - ·중증(severe): 피험자의 정상적인 일상생활(기능)을 불가능하게 하는 경우

12.2.2 시험치료법과의 인과관계 평가

- 이상반응 발현 시 침 치료와의 관련성 여부는 시험자가 다음 기준에 의하여 평가하며, 시험자의 의견을 기술한다.

1) 관련성이 명백함(Definitely related)
- VR기반 오지상승위치료법 또는 오지상승위치료법과 이상반응 발현의 시간적 순서가 타당한 경우
- 이상반응이 다른 어떤 이유보다 오지상승위치료법 또는 VR기반 오지상승위치료법에 의해 가장 개연성이 있게 설명되는 경우
- VR기반 오지상승위치료법 또는 오지상승위치료법 중단으로 이상반응이 사라지는 경우
- 이상반응이 오지상승위치료법 또는 VR기반 오지상승위치료법에 대해 이미 알려져 있는 정보와 일관된 양상을 보이는 경우

2) 관련성이 많음(Probably related)
- VR기반 오지상승위치료법 또는 오지상승위치료법과 이상반응 발현의 시간적 순서가 타당한 경우
- 이상반응이 다른 원인보다 오지상승위치료법 또는 VR기반 오지상승위치료법에 의해 더욱 개연성이 있게 설명되는 경우
- VR기반 오지상승위치료법 또는 오지상승위치료법 중단으로 이상반응이 사라지는 경우

3) 관련성이 의심됨(Possibly related)
- VR기반 오지상승위치료법 또는 오지상승위치료법과 이상반응 발현의 시간적 순서가 타당한 경우
- 이상반응이 다른 가능성이 있는 원인들과 같은 수준으로 오지상승위치료법 또는 VR기반 오지상승위치료법에 기인한다고 판단되는 경우
- 오지상승위치료법 또는 VR기반 오지상승위치료법 중단으로(실시된 경우) 이상반응이 사라지는 경우

4) 관련성이 적음(Probably not related)
- 이상반응에 대해 보다 가능성 있는 원인이 있는 경우
- VR기반 오지상승위치료법 또는 오지상승위치료법 중단결과(실시된 경우)가 음성이거나 모호한 경우
- VR기반 오지상승위치료법 또는 오지상승위치료법(실시된 경우) 결과가 음성이거나 모호한 경우
5) 관련성이 없음(Definitely not related)
- VR기반 오지상승위치료법 또는 오지상승위치료법과 이상반응 발현간의 시간적 순서가 타당하지 않은 경우
- 이상반응에 대해 다른 명백한 원인이 있는 경우
6) 평가 불가능(Unknown)

12.3 안전성 통계처리

-11.3. 의 통계분석법에 따른다.

12.4 이상반응 보고

12.4.1 이상반응 교육

- 시험책임자는 시험담당자 및 피험자 또는 보호자에게 VR기반 오지상승위치료법 혹은 오지상승위치료법 시행 후 나타날 수 있는 모든 이상반응에 대하여 설명하고 치료 후 나타나는 모든 현상에 대하여 보고하도록 교육을 실시한다.

12.4.2 중대한 이상반응의 보고

- 시험담당자는 시험 기간 중 발생한 모든 중대한 이상반응을 오지상승위치료법 혹은 VR 기반 오지상승위치료법과의 관련성 여부와 상관없이 24시간 이내에 아래 원광대학교 산본병원 연락처란에 기록되어 있는 사람에게 보고하여야 하며, 늦어도 다음 근무일에는 보고하여야 한다. 이외에도 시험담당자가 중대하다고 간주하거나, 오지상승위치료법 또는 VR기반 오지상승위치료법과의 사용과 연관 지을 수 있는 유의한 위험, 금기, 부작용, 주의사항을 시사하는 사건 등도 중대한 이상반응으로 기록한다.

성명	소속	연락처
강형원	원광대학교 산본병원	031-390-2763

- 최종 보고 시에는 가능하다면 다음의 정보가 제공되어야 한다. 발생시간, 정도, 처치, 경과, 오지상승위치료법 혹은 VR 기반 오지상승위치료법 시행과의 인과관계 등에 대한 정보를 중대한 이상반응 보고서에 기록하여야 한다.

12.4.3 이상반응 발생 시 조치사항

- 본 시험기간 중 시험책임자, 시험담당자는 환자의 안전에 만전을 기하여야 하며, 예측되지 않은 중대한 이상반응 발생 시에는 신속하고 적절한 조치를 취하여 이상반응을 최소화하여야 한다.
- 임상시험 중 '중대한 이상반응' 발생 시 각 담당자의 의무는 다음과 같다.

시험책임자의 의무
시험책임자는 임상시험 중 중대한 이상반응이 발생한 때에는 즉시 기관임상시험심사위원회에 보고하고 별도의 지시가 있을 때까지 해당 오지상승위요법 및 VR 기반 오지상승위요법에 대한 임상시험의 일부 또는 전부를 중지해야 한다.

시험담당자의 의무<br>시험담당자는 임상시험 실시 중에 중대한 이상반응 등이 발생한 경우에는 즉시 시험책임자 및 기관임상시험심사위원회에 보고하여야 한다.<br><br>의뢰자의 의무<br>의뢰자는 시험책임자 또는 시험담당자로부터 중대한 이상반응을 보고 받은 경우 중대한 이상반응 보고서에 시험책임자 또는 시험담당자로부터 제출 받은 보고서 사본을 첨부하여 보관하고 즉시 적당한 조치를 취하도록 한다.

12.4.4 이상반응의 추적관찰

- 시험자는 이상반응이 나타난 피험자에 대해 증상이 소실되고 상태가 안정될 때까지 피험자를 추적 관찰해야 하며, 의뢰자가 요청하는 경우 이상반응의 이후 진행 경과에 대한 보고서를 제출하여야 한다.

13. 피험자에 대한 이익 및 혜택

-연구 시행을 통해 VR기반 오지상승위치료법 및 오지상승위치료법의 효과성에 대한 과학화에 기여할 수 있고 추후 국가보건의료 정책 수립에 이바지할 수 있다. 피험자가 연구에 참여하는 동안 검사비와 훈련비 등은 무료이고, 1회 내원 시 마다 소정의 교통비(5만원)를 지급한다. 다만, 임상시험 종료 후 대조군의 VR기반 오지상승위치료법은 무료이지만, 교통비는 지급되지 않는다.

14. 피험자의 안전 보호에 관한 대책

- 이 시험은 임상시험 관리기준의 지침 및 헬싱키 선언에 따라 수행된다.

14.1 시험자의 역할

- 시험자(Investigator)는 시험책임자, 시험담당자를 말한다. 시험자는 계획서를 준수하여 임상시험을 실시하여야 한다. 임상시험 중 또는 임상시험 이후에도, 시험자는 임상적으로 의미 있는 임상병리적 검사치의 이상을 포함하여 임상시험에서 발생한 모든 이상반응에 대해 피험자가 적절한 의학적 처치를 받을 수 있도록 조치하여야 하고, 시험자가 알게 된 피험자의 병발 질환에 대해 의학적 처치가 필요한 경우 이를 피험자에게 알려주어야 한다. 시험자는 임상시험계획을 정확히 분석 및 숙지하고, 대상 피험자의 문제점을 적극적으로 대응한다.

14.2 모니터링의 역할

- 모니터링(Monitoring)은 임상시험 진행 과정을 감독하고, 해당 임상시험이 계획서, 표준작업지침서, 임상시험 관리기준 및 관련규정에 따라 실시, 기록되는지 여부를 검토, 확인한다.
- 임상시험에 대한 모니터링은 모니터링 담당자의 정기적인 시험자 방문과 전화로 이루어지도록 한다. 방문 시 모니터링 담당자는 환자기록 원본, 자료보관(연구파일) 등을 확인한다. 또한 모니터링 담당자는 임상시험 진행과정을 잘 살피고, 문제가 있을 경우 시험자와 상의한다.

14.3 피험자 동의

- 피험자 동의(Patient Informed Consent)는 피험자가 임상시험 참여 유무를 결정하기 전에 피험자를 위한 설명서를 통해 해당 임상시험과 관련된 모든 정보를 제공받고, 서명과 서명 날짜가 포함된 문서를 통해 본인이 자발적으로 임상시험에 참여함을 확인하여야 한다.
- 피험자 본인 또는 대리인이 동의서 서식, 피험자설명서 및 기타 문서화된 정보를 읽을 수 없는 경우에는 공

정한 입회자가 동의를 얻는 전 과정에 참석하여야 한다.

- 동의를 얻기 전에 시험자는 피험자 또는 대리인이 임상시험의 세부 사항에 대해 질문하고 해당 임상시험의 참여 여부를 결정할 수 있도록 충분한 시간과 기회를 주어야 하며, 모든 임상시험 관련 질문에 대해 피험자 또는 대리인이 만족할 수 있도록 대답해 주어야 한다.

14.4 임상시험계획서의 승인과 변경

- 임상시험계획서는 기관임상시험심사위원회에 보고하고 승인받아야 한다.
- 임상시험계획서를 시험절차가 더 광범위해지거나 위험도가 높아지거나 환자 선정기준에 변화가 있거나 추가적인 안전성 정보로 인해 임상시험계획서를 변경하는 경우에는 수정을 해야 한다. 임상시험계획서를 수정할 때에는 개정 일자, 개정 이유, 개정 내용 등을 기록하여 보관하여야 하며 기관임상시험심사위원회에 보고해야 한다.
- 시험자는 피험자에게 발생한 즉각적 위험 요소의 제거가 필요한 경우를 제외하고는, 계획서와 다르게 임상시험을 실시하여서는 안 된다.

14.5 피험자 기록의 비밀보장

- 피험자의 신원을 파악할 수 있는 기록은 비밀로 보장될 것이며, 임상시험의 결과가 출판될 경우에도 피험자의 신원을 비밀상태로 유지한다.
- 본 시험에 관련된 의뢰자, 모니터 및 점검자는 본 시험의 모니터링과 점검 및 진행사항 관리를 위한 목적으로 피험자의 기록을 열람할 수 있다. 시험자는 본 계획서에 서명함으로써, 국내의 법규와 윤리적 측면에서 임상시험 의뢰자 또는 임상시험수탁기관의 모니터 및 점검자가 피험자의 차트와 증례기록지 기록을 검증하기 위하여 해당 문서를 검토하거나 복사할 수도 있음을 인정한다. 이러한 정보들은 기밀로 보관되어야 하며, 기밀 보관을 위한 시설과 그 관리기준을 갖추고 있어야 한다.
- 한편, 증례기록지 등 임상시험에 관련된 모든 서류에는 피험자 이름이 아닌 피험자 식별코드(일반적으로 피험자 이니셜)로 기록하고 구분한다.

14.6 자료의 보관

- 임상시험 실시와 관련된 각종 자료 및 기록을 잘 보존하도록 보관하는 장소가 따로 준비되어 있고 보안을 유지하도록 한다. 결과보고서 작성 완료 이후에는 보관책임자를 정하여 임상시험 관련 문서를 임상시험 종료일로부터 3년간 보존하도록 한다.

15. 참고문헌

1) American Psychiatric Association, Diagnostic and statistical manual of mental disorders (DSM-5®). 2013: American Psychiatric Pub.
2) Hong, J., et al., The survey of mental disorders in Korea 2016. Seoul: Ministry of Health and Welfare, 2017.
3) van Minnen, A., et al., Changes in comorbid conditions after prolonged exposure for PTSD: a literature review. Current psychiatry reports, 2015. 17(3): p. 17.
4) Jonas, D.E., et al., Psychological and pharmacological treatments for adults with posttraumatic stress disorder (PTSD). 2013.
5) Lee, D.J., et al., Psychotherapy versus pharmacotherapy for posttraumatic stress disorder: Systemic review and meta-analyses to determine first-line treatments. Depression and anxiety, 2016. 33(9): p. 792-806.
6) Choi, Y.K., Evidence-based treatment of posttraumatic stress disorder. Korean Journal of Clinical Psychology, 2017. 36(4): p. 526-49.
7) Park, A., et al., A Comparative Study between the Korean-medical and the Western-medical Psychotherapy on PTSD. Journal of Oriental Neuropsychiatry, 2018. 29(1): p. 1-8.
8) Kim, J.-H., et al., Development of Korean medical psychotherapy and preliminary clinical trial for Post Traumatic Stress Disorder. Journal of oriental neuropsychiatry, 2015. 26(1): p. 49-61.
9) 서호준; 채정호. 외상 후 스트레스 장애에 대한 인지행동치료의 최근 동향. 인지행동치료, 2006, 6: 117-129.
10) 최수미. 외상 사건 후 PTSD 예방을 위한 치료적 개입 및 모델의 동향분석. 상담학연구, 2015, 16.3: 537-556.
11) Lawyer, Steven R., et al. Predictors of peritraumatic reactions and PTSD following the September 11th terrorist attacks. Psychiatry: Interpersonal and Biological Processes, 2006, 69.2: 130-141.
12) 김명식. PTSD 의 인지행동치료. 한국심리치료학회지, 2009, 1: 45-61.
13) 서호준; 채정호. 외상 후 스트레스 장애에 대한 인지행동치료의 최근 동향. 인지행동치료, 2006, 6: 117-129.
14) Rizzo, Albert A., et al. VR PTSD exposure therapy results with active duty OIF/OEF combatants. Studies in health technology and informatics, 2009, 142: 277-282.
15) 채정호. 외상 후 스트레스 장애의 진단과 병태 생리. 대한정신약물학회지, 2004, 15.1: 14-21.
16) 송창진. 외상후 스트레스 장애의 기전과 치료. 東國醫學,2004,11.1:102-112.
17) Ehlers, Anke; MAERCKER, Andreas; BOOS, Anne. Posttraumatic stress disorder following political imprisonment: The role of mental defeat, alienation, and perceived permanent change. Journal of abnormal psychology, 2000, 109.1: 45.
18) Foa, Edna B., et al. The impact of fear activation and anger on the efficacy of exposure treatment for posttraumatic stress disorder. Behavior Therapy, 1995, 26.3: 487-499.
19) Shapiro Francine; SOLOMON, Roger M. Eye movement desensitization and reprocessing. John Wiley & Sons, Inc., 1995.
20) Shipherd, Jillian C.; STREET, Amy E.; RESICK, Patricia A. Cognitive therapy for posttraumatic stress disorder. Cognitive-behavioral therapies for trauma, 2006, 96-116.
21) 최대헌; 심호규. 심리극집단프로그램이 성매매경험여성의 외상 후 스트레스와 자아존중감에 미치는 영향. 예술심리치료연구, 2013, 9.3: 161-183.
22) 김유경; 최외선; 김갑숙. 제주 4· 3 생존자의 트라우마와 미술치료적 접근. 미술치료연구, 2012, 19: 537-563.
23) 정명선. 성폭력으로 인한 PTSD 아동의 외상중심 미술치료적 접근을 통한 사례연구. 특수교육재활과학연

구, 2012, 51.3: 99-120.
24) 이영애. 학대받은 아동의 게임놀이치료 과정에서 나타난 사회-정서 요인과 치료과정 분석. 한국놀이치료학회지 (놀이치료연구), 2016, 19.2: 69-101.
25) Kim, Young-Dae, et al. Acupuncture for posttraumatic stress disorder: a systematic review of randomized controlled trials and prospective clinical trials. Evidence-Based Complementary and Alternative Medicine, 2013, 2013.
26) Gerardi M, Rothbaum BO, Ressler K, Heekin M, Rizzo A. Virtual reality exposure therapy using a virtual Iraq: case report. Journal of Traumatic stress: official Publication of The International society for Traumatic stress studies, 2008;21(2):209-213.
27) Imreratori C, Dakanalis A, Farina B, Pallavicini F, Colmegna F, Mantovani F, Clerici M. Global Storm of Stress-Related Psychopathological Symptoms: a brief overview on the usefulness of virtual reality in facing the mental health impact of COVID-19. Cyberpsychology, Behavior, and Social Networking, 2020;23(11):782-88.
28) FREEMAN, Daniel, et al. Automated psychological therapy using immersive virtual reality for treatment of fear of heights: a single-blind, parallel-group, randomised controlled trial. The Lancet Psychiatry, 2018, 5.8: 625-632.
29) RIVA, Giuseppe. Virtual reality in psychotherapy. Cyberpsychology & behavior, 2005, 8.3: 220-230.
30) 김지혁, 황의완 編著. 동의정신의학. 서울:현대의학서적사. 1992:157, 783-791.
31) 전황수. 가상현실(VR)의 의료분야 적용 동향. 전자통신동향분석. 2019;34(2):19-28
32) Ku JH, Jang DP, Shin MH, Cho JJ, Ahn HB, Jo BH, Kim IYg, Kim SI. Experimental production of a virtual speech simulator for the treatment of interphobia. Journal of the Korean Society of Medical Biomedical Engineering. 2000;21(6):615-21
33) JANG, DP, Ku JH, Choi YH, Wiederhold BK, Nam SW, Kim IY, Kim SI. The development of virtual reality therapy (VRT) system for the treatment of acrophobia and therapeutic case. IEEE Transactions on Information Technology in Biomedicine, 2002;6(3):213-217.
34) Kim W, Jeong CH, Lee WI, Che JH. The Effects of Exoposure Therapy using Head Mount Display on Test Anxiety of the Students Preparing College Entrance Examination: A preliminary study. Cognitive Behavior Therapy in Korea, 2004;4(2):85-95.
35) North MM, North, SM, Joseph R. Virtual reality therapy: an effective treatment for the fear of public speaking. International Journal of Virtual Reality, 1998;3(3):1-6.
36) Yoon HY, Park KW, Cho SH. Development of virtual reality exposure program in the treatment of social anxiety disorder. Journal of Research Methodology 2019;4(3):11, 63-88.
37) Park SE, Ryu JH, Beack SH. The Development of Virtual Reality Therapy(VRT) System for the Treatment of Acrophobia.Journal of the Korean Institute of Electrical Engineers D, 2001;50(10):486-93.
38) Lee TS, Kim MJ, Kim HY, Bang EB, Kim YR. The Application of Virtual Reality to Anxiety Reduction. The Korean Psychological Association, 2018:183-183.
39) Gerardi M, Rothbaum BO, Ressler K, Heekin M, Rizzo A. Virtual reality exposure therapy using a virtual Iraq: case report. Journal of Traumatic stress: official Publication of The International society for Traumatic stress studies, 2008;21(2):209-213.
40) Imreratori C, Dakanalis A, Farina B, Pallavicini F, Colmegna F, Mantovani F, Clerici M. Global Storm of Stress-Related Psychopathological Symptoms: a brief overview on the usefulness of virtual reality in facing the mental health impact of COVID-19. Cyberpsychology, Behavior, and Social Networking, 2020;23(11):782-88.
41) Sebastian, B., &Nelms, J. (2017). The effectiveness of Emotional Freedom Techniques in the

treatment of posttraumatic stress disorder: A meta-analysis. Explore, 13(1), 16-25.
42) 박대명, 이상룡, 강위창, 정인철: 驚悸怔忡변증도구 개발을 위한 기초 연구, 동의신경정신과학회지, 2010;21(2):1-16.
43) Weathers FW, Litz BT, Herman DS, Juska JA, Keane TM. The PTSD Checklist (PCL): Reliability, validity, and diagnostic utility. annual meeting of the International Society for Traumatic Studies. San Antonio, TX;1993.
44) 김종원, 정혜경, 최진희, 소형석, 강석훈, 김동수, 문정윤, 김태용. 월남전 참전 노인에서 한글판 외상후 스트레스 장애 체크리스트-5의 정신측정학적 특성. Anxiety and Mood. 2017;13(2):123-131.
45) Wikipedia <Clinical Global Impression> 2017.7.17. (https://en.wikipedia.org/wiki/Clinical_Global_Impression)
46) Guy, William (1976). "Clinical Global Impressions". ECDEU Assessment Manual for Psychopharmacology—Revised. Rockville, MD: U.S. Department of Health, Education, and Welfare; Public Health Service, Alcohol; Drug Abuse, and Mental Health Administration; National Institute of Mental Health; Psychopharmacology Research Branch; Division of Extramural Research Programs. pp. 218–222. OCLC 2344751. DHEW Publ No ADM 76-338 - via Internet Archive.
47) Guy, W. (2000). "Clinical Global Impressions (CGI) Scale, Modified". In Rush, John A.; Task Force for the Handbook of Psychiatric Measures. Handbook of Psychiatric Measures (1st ed.). Washington, DC: American Psychiatric Association. ISBN 978-0-89042-415-5. OCLC 43483679.
48) Tedeschi, R.G. and L.G. Calhoun, The Posttraumatic Growth Inventory: measuring the positive legacy of trauma. J Trauma Stress, 1996. 9(3): p. 455-71.
49) 송승훈, 김교헌, 이홍석, 박준호. 한국판 외상후 성장 척도의 타당도 및 신뢰도 연구. 한국심리학회지: 건강, 2009;14(1):193-214.
50) Baer, R. A., Smith, G. T., &Allen, K. B. (2004). Assessment of mindfulness by self-report: The Kentucky Inventory of Mindfulness Skills. Assessment , 11 (3), 191-206.
51) 원두리, &김교헌. (2006). 한국판 5 요인 마음챙김 척도의 타당화 연구. 한국심리학회지: 건강 , 11 (4), 871-886.
52) 정문주, 채은영, 류영수, &강형원. (2017). 한국판 5 요인 마음챙김 단축형 척도 타당화 연구. 동의신경정신과학회지 , 28 (3), 207-216.
53) Kroenke, K., R.L. Spitzer, and J.B. Williams, The PHQ-15: validity of a new measure for evaluating the severity of somatic symptoms. Psychosom Med, 2002. 64(2): p. 258-66.
54) Han, C., et al., Psychometric properties of the Patient Health Questionnaire-15 (PHQ-15) for measuring the somatic symptoms of psychiatric outpatients. Psychosomatics, 2009. 50(6): p. 580-5.
55) Zigmond AS, Snaith RP: The hospital anxiety and depression scale. Acta Psychiatr Scand 1983;7:361-370.
56) Oh SM, Min KJ, Park DB: A study on the standardization of the hospital anxiety and depression cale for Koreans: a comparison of normal, depressed and anxious groups. J Korean Neuropsychiatr Assoc 1999;8:289-296.
57) Bastien CH, Vallières A, Morin CM. Validation of the Insomnia Severity Index as an outcome measure for insomnia research. Sleep Med. 2001 Jul;2(4):297-307.
58) Cho YW, Song ML, Morin CM. Validation of a Korean version of the insomnia severity index. J Clin Neurol. 2014 Jul;10(3):210-5.
59) Mimeault V, Morin CM. Self-help treatment for insomnia: bibliotherapy with and without professional guidance. J Consult Clin Psychol. 1999 Aug;67(4):511-519.
60) 권정혜, 김종우, 박동건, 이민수, 민성실, 권호인 : 화병척도의 개발과 타당소 연구, 한국심리학회지 임상, 2008. 27(1):237-252.

61) Kim TH, Jo MW, Lee SI, et al. Psychometric properties of the EQ-5D-5L in the general population of South Korea. Qual Life Res. 2013;22:2245-2253.

62) Feng Y, Parkin D, Devlin NJ. Assessing the performance of the EQ-VAS in the NHS PROMs programme. Qual Life Res. 2014;23(3):977-989.

63) Kim JH, Sue JH, Lee GE, Kim NK, Choi SY, Lyu YS, Kang HW. Development of Korean medical psychotherapy and preliminary clinical trial for post traumatic stress disorder. J. of Oriental Neuropsychiatry. 2015;26(1):49-61.

64) 유수양, 강형원. 마음의 방 그리기에 관한 양적, 질적 분석 연구. 동의신경정신과학회지 2017;28(3):275-286

65) 유수양. 관계성 확립이 신문혈 자침의 득기감과 정량화 뇌파변화에 미치는 임상연구. 원광대학교 일반대학원 박사학위. 2018.

66) Cheong MJ, Lee GE, Lee YS, Bae KH, Kang YK, Kim JH, Lyu YS, Kang HW. Validation of the Core Seven-Emotions Inventory - short form. Integrative Medicine Research. 2019;8:116-119.

67) Wolpe, J. (1989). The Practice of Behavior Therapy. (1st ed.). New York, NY:Pergamon Press.

68) Pomeranz B, Macaulay RJ, Caudill MA, Kutz I, Adam D, Gordon D, Kilborn KM, Barger AC, Shannon DC, Cohen RJ, Benson H. Assessment of autonomic function in humans by heart rate spectral analysis. Am J Physiol. 1985;248:H151-3.

69) Prins A, Bovin MJ, Smolenski DJ, Marx BP, Kimerling R, Jenkins-Guarnieri MA, Kaloupek DG, Schnurr PP, Kaiser AP, Leyva YE, Tiet QQ. The primary care PTSD screen for DSM-5 (PC-PTSD-5): development and evaluation within a veteran primary care sample. Journal of General Internal Medicine. 2016;31(10):1206-11.

70) Park JE, Kim WH, Roh DY, Won SD, Kim HK, Kang SH, Hong NR, Park SY, Kim DH, Chae JH. Workbook for assessment in disaster behavioral health. Seoul:Korean academy of anxiety and mood. 2016:5-6.
