# Supplementary material for: Virtual reality-based emotion-to-emotion therapy for post-traumatic stress disorder: Protocol for a randomized controlled trial of efficacy, safety, and cost-effectiveness
Source: PLoS One. 2026 Sep 2;21(9):e0357593. doi: 10.1371/journal.pone.0357593 (PMC13537590; doi:10.1371/journal.pone.0357593)
Supplement: Supplementary file 3 [file pone.0357593.s003.docx]

**PROTOCOL**

Clinical trial to evaluate the safety, efficacy, and economic feasibility of virtual reality-based ‘Emotion to Emotion Therapy’ for post-traumatic stress

disorder patients

| **Protocol No.** | **WS_NP_2022_01** |
| --- | --- |
| **Protocol Ver.** | **Ver. 2.0** |
| **Study Center** | Wonkwang University Sanbon Hospital |
| **Principle Investigator** | **Hyung Won Kang** |
| **Date** | **2022.09.25** |

***CONFIDENTIAL***

All information related to this protocol shall be kept confidential and may not be disclosed without prior written consent..

**▣ Protocol Revision History**

| 버전 번호 | 일자 | 주요 내용 |
| --- | --- | --- |
| Ver. 1.0 | 2022.07.28. | Initial draft of the clinical study protocol. |
| Ver. 2.0 | 2022.09.25. | Added economic evaluation-related content and revised CRF |
|  |  |  |
|  |  |  |
|  |  |  |
|  |  |  |
|  |  |  |
|  |  |  |
|  |  |  |
|  |  |  |
|  |  |  |
|  |  |  |
|  |  |  |

Protocol Agreement

Clinical trial to evaluate the safety, efficacy, and economic feasibility of virtual reality-based ‘Emotion to Emotion Therapy’ for post-traumatic stress disorder patients

Protocol Number: WS_NP_2022_01

Version: 2.0

Date Prepared: 2022.09.25.

- I have read the protocol and hereby agree to conduct this study in accordance with all applicable regulations and guidelines.
- I hereby agree to hold in strict confidence all information developed or obtained in relation to this protocol.

### PrincipaL Investigator

Hyung Won Kang

Wonkwang University Sanbon Hospital

**2022.09.25.**

**Date**

**Table of Contents**

## ▣ Clinical Trial Protocol Overview · 1

1. Clinical Trial Title	7
2. Clinical Trial Institution, Principal Investigator/Manager, and Sponsor	7

## 3. Purpose and Background of the Clinical Trial 7

## 4. Taget Disease and Clinical Trial Period · 10

## 5. Subject Inclusion Criteria, Exclusion Criteria, and Target subjects · 10

## 6. Clinical Trial Methods · 12

## 7. Observational Items, Observational Test Methods, and Schedule · 17

## 8. Expected Adverse Events · 27

9. **Discontinuation and Withdrawal Criteria	·** 28

## 10. Evaluation Criteria and Methods · 29

11. **Statistical Analysis Methods	· 29**

## 12. Safety Evaluation Criteria, Evaluation Methods, and Reporting Methods, Including Adverse Reactions · 33

| **13. Benefits to Subjects** | **·** 35 |
| --- | --- |
| **14. Mesures to Protect Subject Safety** | **·** 35 |
| **15. References** | **·** 37 |

# 1. Clinical Trial Title

**Clinical trial to evaluate the safety, efficacy, and economic feasibility of virtual reality-based ‘Emotion to Emotion Therapy’ for post-traumatic stress disorder patients**

# 2. Clinical Trial Institution, Clinical Trial Principal Investigator/Person in Charge, and Sponsor

**2.1 Lead Institution and Address**

**Wonkwang University Industry-Academic Cooperation Foundation**

**4th Floor, Wonkwang University Main Building, 460 Iksan-daero, Iksan-si, Jeollabuk-do, Korea**

**Industry-Academic Cooperation Foundation Phone: 063-850-5952**

**2.2 Implementing Institution and Address**

**Wonkwang University Sanbon Hospital**

**321 Sanbon-ro, Gunpo-si, Gyeonggi-do, Korea**

**Phone: 031-390-2763**

**2.3 Clinical Trial Sponsor and Address**

**Korea Health Industry Development Institute**

**Osong Health and Medical Administration Town, 187 Osongsaengmyeong 2-ro, Osong-eup, Heungdeok-gu, Cheongju-si, Chungcheongbuk-do, Korea**

**Korea Health Industry Development Institute Phone: 034-713-8000**

# 3. Purpose and background of the clinical trial

**3.1 Purpose of the Clinical Trial**

**○ This study aimed to evaluate the efficacy, safety, and economic feasibility of a virtual reality-based Ojisangeunwi therapy for patients with post-traumatic stress disorder (PTSD) to explore its clinical applicability and to propose a VR-based Korean medicine treatment model.**

**3.2 Background of the Clinical Trial**

○ Overview of PTSD

Posttraumatic stress disorder (PTSD) is a condition characterized by the following symptoms following exposure to a traumatic event, such as death, serious injury, or sexual violence. These symptoms include recurrent and distressing memories of the traumatic event, intrusive symptoms such as dreams, persistent avoidance of stimuli associated with the traumatic event, negative cognitive and emotional changes related to the traumatic event, and marked changes in arousal and reactivity related to the traumatic event [1]. According to a 2016 report on the epidemiological survey of mental disorders, the lifetime prevalence of PTSD was reported to be 1.5% [2]. Patients with PTSD often also have coexisting depressive, substance use, and anxiety disorders [3].

### The first treatment considered for PTSD is trauma-focused psychotherapy [4, 5]. Cognitive behavioral therapy/cognitive therapy, cognitive processing therapy, exposure therapy/prolonged exposure therapy, eye movement desensitization and reprocessing (EMDR), narrative exposure therapy, stress inoculation training, and stage-based therapy are being applied as psychotherapy for PTSD [6]. Traditional Korean medicine psychotherapy includes Gyeongjapyeong Ji therapy, Jieongoron therapy, Ijeongbyeongi therapy, and Ojisangseung therapy [7], and a PTSD psychotherapy program based on traditional Korean medicine psychotherapy has also been developed [8].

**○ Virtual Reality (VR) as a Technology Suitable for Mental Health Management, Including PTSD**

- Virtual reality (VR) is a useful technology for education and training to relieve psychological stress, such as anxiety and depression. Since its use in the treatment of acrophobia in the 1990s, VR has been utilized in research on mental illness, stress, and pain relief.
- Due to the COVID-19 pandemic, VR use has increased in various fields since 2020. Interest in VRT, particularly in psychotherapy, where face-to-face contact is essential, has grown.
- The application of VRT (Virtual Reality Therapy) is increasing in various fields, particularly in the United States, with a particular surge in psychiatric treatment. VR-based psychotherapy is being applied in a variety of fields, from the diagnosis of various mental disorders to the treatment of PTSD to addiction, neurosis, depression, and anxiety [26][27]. For PTSD patients (those who have experienced the World Trade Center attack or war scenes), VR is being used as an alternative imaginary technique by reenacting situations that would be impossible to apply in real life. In particular, VR has been consistently reported to reduce the risk of actual exposure and enhance therapeutic effectiveness by enabling treatment tailored to the patient's level in a controlled environment.

### ○ Limitations of VR Technology in Psychotherapy and Overcoming Strategies

- Recently, interest in non-face-to-face therapeutic interventions has increased due to advancements in science and technology and the social environment [28].

- The advantage of VR psychotherapy is that it treats patients with psychological distress and emotional anxiety by altering their emotions, mindset, and emotional states in a virtual space similar to reality [29].

- However, since the early 1990s, commercialization of content for treating post-traumatic stress disorder, phobias, and anxiety has failed. This is believed to be due to the limited availability of psychotherapy content, which is also a result of the limitations of VR technology.

- The rich content of Korean culture is a valuable medium for psychotherapy. Combining Korean medicine psychotherapy with VR technology to leverage this content could usher in a new era in the field of VR psychotherapy.

## 3.4 4. Study Content, Scope, and Methods

## ○ Subject Selection

## - This clinical trial will recruit subjects who voluntarily signed a consent form through open recruitment. Subjects will be selected based on the inclusion and exclusion criteria outlined in the clinical trial protocol if deemed appropriate.

## ○ Blocked Randomization

## - A randomization officer (or statistician) not involved in the conduct or evaluation of the clinical trial will generate a randomization list using a specific plan and reproducible method. The randomization list will be sealed and stored in a manner that allows for verification of unsealing. The principal investigator will manage the randomization list separately.

- The randomization officer uses the statistical program StataMP 16 (StataCorp LLC, 4905 Lakeway Drive, College Station, Texas 77845 USA) to randomly assign subjects to each group with an equal chance of selection. Randomization into experimental groups 1 and 2 and the control group is performed in a 1:1:1 ratio using block randomization, regardless of the conducting site.
- The investigator opens the randomization envelopes in front of the subjects in order to assign subjects. The opened envelopes are then marked with the date of opening and the investigator's signature and stored separately.
- The subject identification codes on the randomization envelopes and randomization sheets represent the randomization numbers.

○ Evaluation of Test Groups 1 and 2 (VR-based Remote Ascension Therapy Group, Remote Ascension Therapy Group) and the Control Group

- - Both Test Groups 1 and 2 and the control group will administer a baseline questionnaire and a borderline-jeongchung dialectical assessment tool. Test Group 1 will receive VR-based Remote Ascension Therapy, Test Group 2 will receive Remote Ascension Therapy, and the control group will receive no other program.
- VR-based Remote Ascension Therapy or Remote Ascension Therapy will be administered for 8 weeks. The test and control groups will be administered questionnaires assessing the primary and secondary efficacy at Weeks 0, 8, and 12. A questionnaire assessing economic feasibility will be administered at Weeks 0, 4, and 12. Device testing will be performed at Weeks 0 and 8. The primary validity assessment will be conducted using the PCL-5-K, while secondary validity assessments will include the CGI-I, CSEI-S, K-PTGI, FFMQ-S, PHQ-15, HADS, ISI-K, Hwabyung Scale, SUDS, and MRM. However, the CSEI-S, SUDS, and MRM will be assessed before and after each session. For the economic evaluation, the EQ-5D, EQ-VAS, and cost-effectiveness questionnaires will be administered.
- Heart rate variability (HRV) and quantitative electroencephalography (QEEQ) analyses will be performed through device testing. Quantitative EEG measurements will be taken for 10 minutes, with eyes closed for 5 minutes and eyes open for 5 minutes before and after the intervention.
- After all experiments are completed, the desired control group will undergo follow-up with either virtual reality or Ojisangseungyo therapy.

○ Evaluation Method and Procedure

- Each evaluation will conduct a difference test and correlation analysis to analyze the effectiveness between and within the three groups (Trial Group 1, Trial Group 2, and the control group).
- An economic evaluation will then be conducted to determine the cost-benefit ratio of both VR-based and Ojiseungseungheup Therapy.

## 3.5 Expected Effects and Application of Research Results

## ○ This study presents a case study for the development of a new technology that can enhance the effectiveness of Korean medicine psychotherapy through the integration of VR and Ojisangeunwi therapy.

## ○ It presents the potential and basic evidence for developing treatments utilizing new technologies like VR for psychiatric disorders such as PTSD.

## ○ By identifying the correlation between the main and secondary efficacy assessment scales and heart rate variability and brainwave changes before and after VR-based Ojisangeunwi therapy, the respective effectiveness of VR-based Ojisangeunwi therapy for PTSD can be predicted.

## ○ The improvement of PTSD symptoms through Korean medicine treatment can contribute to an improved quality of life for patients, their families, and members of society.

# 4. Target Conditions and Clinical Trial Period

4.1 Target Conditions

Patients diagnosed with PTSD according to the Structured Clinical Interview for DSM-5 (SCID-5)

4.2 Clinical Trial Period

From the date of clinical trial approval to December 31, 2026

# 5. Subject selection criteria, exclusion criteria, and target number of subjects and basis for selection

**5.1 Selection Criteria**

**Patients who meet all of the following criteria will be selected for the study:**

**1) Adults aged 18 to 70 years.**

**2) Diagnosed with post-traumatic stress disorder using the Structured Clinical Interview for DSM-5 (SCID-5).**

**3) Scoring 3 or higher on the PC-PTSD-5.**

**4) Voluntarily participating and signing a consent form.**

**5.2 Exclusion Criteria**

**Patients who meet the following criteria will be excluded: 1) Patients with a severe psychiatric disorder**

**① Those with recent or past medical history of delusions or hallucinations**

**② Those with at least one episode of mania**

**③ Those with a history of alcohol abuse or dependence**

**④ Those at risk of suicide**

**2) Those requiring continuous administration of substances deemed to affect symptom induction, excluding concomitant medications**

**3) Those with congenital diseases, diseases of the central nervous system, peripheral nervous system, endocrine system, immunological diseases, or serious heart, liver, or kidney problems**

**4) Those who are pregnant or breastfeeding**

**5) Those who have participated in another clinical trial within the past month or are currently participating in another clinical trial**

**6) Those who are deemed difficult to conduct by the clinical trial manager or clinical trial principal investigator**

**5.3 Concomitant Medications**

**In principle, non-pharmacological treatments other than VR-based or orthostatic hypotension therapy will not be administered during the trial. However, the following non-pharmacological treatments and concomitant medications may be considered for participation. However, if necessary, they may be administered at the trial manager's discretion based on the following criteria:**

**1) Counseling treatment for PTSD must have been administered stably for at least 3 months prior to screening.**

**2) For patients taking hypnotics, antidepressants, antipsychotics, or anxiolytics, the dosage must have been stable for the past 4 weeks.**

**3) All medications for other conditions (e.g., hypertension, diabetes, hyperlipidemia, painkillers) previously administered prior to the trial are permitted.**

**5.4 Target Number of Subjects and Basis for Estimation**

**Number of Subjects for Efficacy Assessment: 60; Number of Subjects for Efficacy Assessment: 20/Group**

**Considering a 20% dropout rate, final number of subjects assigned: 24/Group; Target Number of Subjects: 72.**

**♠ Basis for Determining the Subject Size**

**1) Quantitative Calculation using G*Power 3.1.9.4**

**The quantitative calculation results from G*Power 3.1.9.4 indicate an effect size of 0.5, a 95% confidence interval for power, three groups, and a required number of subjects for covariance analysis of 65. This provides statistical basis for reliably securing significance within ±10% of the 60 subjects suggested in this study, which is 72 subjects.**

61

65

0.9502209

Total sample size =

Actual power	=

=

3.1477912

Critical F =

Denominator df

1

=

Numerator df

0.95

0.5

0.05

= 2

3

=

=

Power (1-β err prob)

Number of groups = Number of covariates

**Output:** Noncentrality parameter ë

16.2500000

**F tests -** ANCOVA: Fixed effects, main effects and

interactions

**Analysis:** A priori: Compute required sample size **Input:**	Effect size f	=

α err prob	=

**2) Prior Research Evidence**

**Sebastian, B., & Nelms, J. (2017)[41] conducted a meta-analysis of the effectiveness of EFT for PTSD, with the study subjects ranging from 8 to 32.**

**Most studies analyzed the effectiveness of EFT with fewer than 10 subjects. Therefore, the study's inclusion of 20 subjects per group likely provides a theoretical basis for its effectiveness.**

Sebastian, B., &Nelms, J. (2017). The effectiveness of Emotional Freedom Techniques in the treatment of posttraumatic stress disorder: A meta-analysis. *Explore*, *13*(1), 16-25.

# 6. Clinical trial method

6.1 Clinical Trial Design

- Randomized, Controlled, Singlecenter Clinical Trial

- The final PTSD patients will be randomly assigned to a VR-based remote ascent therapy group (hereafter, Test Group 1), a remote ascent therapy group (hereafter, Test Group 2), and a control group, based on block randomization. Test Group 1 will receive VR-based remote ascent therapy twice a week for 40 minutes per session for 8 weeks, while Test Group 2 will receive remote ascent therapy alone. Follow-up will occur after 12 weeks. The control group will wait for 8 weeks, continuing their normal daily routine. During this wait, changes in stressful situations will be monitored by phone every two weeks, and patients will undergo the same assessments and questionnaires as the test group at weeks 0, 4, and 8. Follow-up will occur after 12 weeks.

- In order to investigate the effects of VR-based remote ascending posture therapy and remote ascending posture therapy in test groups 1 and 2, heart rate variability (HRV) and quantifiable electroencephalogram (qEEG) analysis were conducted before and after the intervention, and a questionnaire was administered using the main efficacy assessment and secondary efficacy assessment.

- We will conduct a correlation analysis to determine the effects of VR-based euphoria therapy and euphoria therapy in Study Groups 1 and 2, as well as the predictors of pre- and post-intervention PTSD using heart rate variability (HRV) and quantified electroencephalography (qEEG) data. We will also examine the effects of both the VR-based euphoria therapy and euphoria therapy on pre- and post-intervention outcomes ..., as well as the predictors of PTSD on the primary and secondary efficacy assessments.

-For Study Groups 1 and 2, we will conduct a cost-utility analysis to assess the similarities between euphoria therapy and cognitive behavioral therapy. We will assess the changes in the EQ-5D (EuroQoL-5D) and EQ-VAS scores for each unit increase in VR-based euphoria therapy and euphoria therapy. We will also conduct a cost-effectiveness assessment based on the similarities between euphoria therapy and cognitive behavioral therapy.

6.2 Summary of Clinical Trial Design

A. Clinical Trial Design Phase

- Among the applicants who applied for this clinical trial, those diagnosed with PTSD using the Structured Clinical Interview for DSM-5 (SCID-5) will be selected as subjects.

- After selection, the Borderline Intimacy Diagnosis Tool will be administered as a baseline questionnaire.

- Experimental Group 1 will receive VR-based 40-minute therapy twice a week for 8 weeks, with a follow-up 12 weeks later. Experimental Group 2 will receive 40-minute therapy twice a week for 8 weeks, with a follow-up 12 weeks later. The control group will remain in their normal daily routine for 8 weeks. During this waiting period, participants will be phoned every two weeks to check for any changes in anxiety and stress levels. Participants will also visit the clinic at weeks 0, 4, and 8 to complete the same tests and questionnaires as the experimental group. A follow-up will be conducted 12 weeks later. - At the 4th week (8 sessions), 8th week (16 sessions), and 12th week follow-up points, 4 weeks after the end of the intervention, subjects were subjected to check for any adverse reactions.

### B. Clinical Trial Questionnaire and Measurement Evaluation

### - As a baseline survey, medical staff will explore the borderline symptom assessment tool at Week 0.

### Symptom assessment will be conducted for study groups 1 and 2 and the control group at Week 0 (session 1), Week 8 (session 16), and Follow-up Week 12. The primary assessment questionnaire, the PCL-5-K, and the secondary assessment questionnaires, the CGI-I, K-PTGI, FFMQ-S, PHQ-15, HADS, ISI-K, and Hwabyung Scale, will be used.

### - To conduct an economic evaluation, the EQ-5D, EQ-VAS, and cost measurement questionnaires will be administered to study groups 1 and 2 and the control group at Week 0 (session 1), Week 4 (session 8), Week 8 (session 16), and Follow-up Week 12. - Pre- and post-session evaluations will be conducted on groups 1 and 2 to assess the subjects' stability and changes. The Mind Map (MRM), CSEI-S, and SUDS will be administered before and after the intervention.

### - HRV analysis will be conducted on groups 1 and 2 diagnosed with PTSD and the control group at time 0 (session 1) and week 8 (session 16) to measure heart rate variability. Quantitative electroencephalograms (QEs) will be measured to determine the subjects' absolute power (μV2), relative power (%), Z-score, and L-measurement values for each frequency band.

| Enroll | |
| --- | --- |
|  |  |
| Screening | |

| Allocation | |
| --- | --- |
|  |  |
| ①Experimental group1  ②Experimental group 2 | |
| ③Control Group | |

| Before Intervention session #1 | |
| --- | --- |
|  |  |
| ①기초설문 (경계정충변증도 구)  ② 증 상 평 가 설 문 (PCL-5_K,CGI-I, K-PTGI,FFMQ-s,P HQ-15, HADS,  ISI-K, 화병척도)  ③ 경 제 성 평 가 (EQ-5D,  EQ-VAS,비용측정)  ④전후평가 ( MRM, CSEI-S, SUDS)  ⑤기기검사 (HRV, QEEG) | |

| 1-7  Before and After Interventi  on | |
| --- | --- |
|  |  |
| 전후평가 (MRM,  C S E I - S , SUDS)  이 상 반 응 및 순응도 확인 | |
| - | |

| After Intervention 8 sessions | |
| --- | --- |
|  |  |
| ① 경 제 성 평 가 (EQ-5D,  EQ-VAS,비용측정)  ②전후평가 ( MRM, CSEI-S, SUDS)  ③이상반응확인 | |
| 경 제 성 평 가 (EQ-5D,  EQ-VAS,비용측정) | |

| 9-15  Before and After Interventi  on | |
| --- | --- |
|  |  |
| 전후평가 (MRM,  C S E I - S , SUDS)  이 상 반 응 및 순응도 확인 | |
| - | |

| After Intervention 16 sessions | |
| --- | --- |
|  |  |
| ① 증 상 평 가 설 문 (PCL-5_K,CGI-I, K-PTGI,FFMQ-s,P HQ-15, HADS,  ISI-K, 화병척도)  ② 경 제 성 평 가 (EQ-5D,  EQ-VAS,비용측정)  ③전후평가 ( MRM, CSEI-S, SUDS)  ④기기검사 (HRV, QEEG)  ⑤이상반응 및 순응도 확인 | |

| Follow up | |
| --- | --- |
|  |  |
| ① 증 상 평 가 설 문 (PCL-5_K,CGI-I, K-PTGI,FFMQ-s,P HQ-15, HADS,  ISI-K, 화병척도)  ② 경 제 성 평 가 (EQ-5D,  EQ-VAS,비용측정)  ③전후평가 ( MRM, CSEI-S, SUDS)  ④이상반응 및 순응도 확인 | |

→

## Figure 4. Summary of clinical trial design.

ISI-K; Korean version of Insomnia severity index CSEI-S; The Core Seven-Emotions Inventory

Short Form

SUDS; Subjective Units of Distress Scale MRM; Mentalizing the Rooms of Mind EQ-5D; EuroQoL-5Dimension

EQ-VAS; EuroQoL-visual analog scale HRV; Heart Rate Variation

QEEG; Quantative Encephalogram

PCL-5-K:	The Posttraumatic	Stress	Disorder Checklist for DSM-5

K-PTGI;	Korea-	Posttraumatic	Growth Inventory-Short Form

CGI-I; The Clinical Global Impression–Improvement scale

FFMQ-S;	Five	Facet	Mindfulness	Questionnaire Short Form

PHQ-15; Patient Health Questionnaire-15

HADS; Hospital Anxiety and Depression Scale

6.3 Oji Sangseungwi Therapy and VR-Based Oji Sangseungwi Therapy for PTSD Patients

6.3.1 Oji Sangseungwi Therapy and Its Implementation Method

- Oji Sangseungwi Therapy is a unique psychotherapy method in Korean medicine that applies the theory of the Five Elements (Oxygen and Inhibition), a fundamental theory of Korean medicine, to psychotherapy. It assigns emotions to each of the five viscera, and these emotions dynamically influence each other. Treatment involves regulating this dynamic relationship. The method involves analyzing the patient's emotions and feelings and utilizing the corresponding emotions and feelings within the Five Elements theory to help the patient achieve a stable state.

- In other words, when exposed to external environmental or emotional stimuli, patients exhibit excessive, pathological emotions. This technique involves the therapist inducing specific emotions in the patient to regulate these pathological emotions.

○ Oji Sangseungwi Therapy Method Implementation

- Oji Sangseungwi Therapy consists of the following stages: assessment, analysis, training, reevaluation, and education. (Total 40 minutes)

(1) Assessment: Patient's emotional and affective assessment, physical symptom assessment (CSEI-S, MRM, SUDS assessment) (5 minutes)

(2) Analysis: The connection between emotions and physical symptoms, yin-yang and five elements analysis of emotions and sensations, identification of core emotions, Oji Sangseungwi Therapy method selection (5 minutes)

(3) Training: Emotion-inducing meditation, breathing meditation, bodily sensation meditation, upper tian meditation, joy meditation, lower tian meditation, middle tian meditation, resource meditation, integrated-samdanjeon meditation (20 minutes)

(4) Evaluation: Post-training assessment of current emotions and physical symptoms (CSEI-S, MRM, SUDS assessment) (5 minutes)

(5) Emotion regulation training (5 minutes)

For PTSD patients, fear is a characteristic emotion. The Oji Sangseungwi therapy focuses on the Sasenggong (思勝恐) method, which helps them overcome fear cognitively. This training involves meditation on the upper abdomen. The contents of this meditation are outlined in the table below.

- In addition to upper body meditation, other training meditations can be performed depending on the patient's emotional state.

<Sangdanjeon Mindfulness Meditation Guide>

First, find a comfortable posture that suits your body. Straighten your back and place your hands lightly on your knees. Then, try to relax as much as possible.

Release the tension in your shoulders and let your hands and fingers rest. Simply feel your body relax,

noticing your body becoming loose like this.

Now, let's briefly bring your awareness to your breath. Follow the natural flow of your inhale and exhale. Breathe in through your nose and breathe deeply.

Then, slowly... exhale. "Whew" (making a sound). Inhale deeply again, and slowly, "Whew" (making a sound). Exhale. Follow this natural flow of breathing.

Now, shift your awareness to the Sangdanjeon, located in the center of your forehead. The Sangdanjeon is the seat of awareness.

It's the seat of insight.

Concentrate quietly on the Sangdanjeon, and if anything comes to mind or insight, try to verbalize it.

Ah, so that's it? Ah, that's how it was?

Store the verbalized sensation there.

You can relive this stored sensation whenever you need it. It's the sensation of my upper body, with insight and awareness.

Slowly open your eyes and return to your original position.

6.3.2 VR-Based Ojiseunghyang Therapy Implementation Method

- VR-based Ojiseunghyang Therapy utilizes software that can create virtual reality (VR) through a head-mounted display (HMD) or a computer and projector set. This method utilizes a realistically implemented virtual environment, not confined to the clinical setting, to train patients to stabilize their emotions by counteracting problematic emotions. Software and applications containing Ojiseunghyang Therapy content are installed on commercially available equipment, such as a desktop computer or Gear VR.
- VR-based Ojiseunghyang Therapy consists of the same stages as conventional Ojiseunghyang Therapy: assessment, analysis, training, reevaluation, and education. (Total 40 minutes)

(1) Assessment: Patient's emotional and affective assessment, physical symptom assessment (CSEI-S, MRM, SUDS assessment) (5 minutes)

(2) Analysis: The connection between emotions and physical symptoms, Yin-Yang and Five Elements analysis of emotions and sensations, identification of core emotions, selection of Oji Sangseung therapy (5 minutes)

(3) Training (VR): Emotion-inducing meditation, breathing meditation, bodily sensation meditation, Sangdanjeon meditation, joy meditation, Hadanjeon meditation, pre-cession meditation, resource meditation, integrated-Samdanjeon meditation (20 minutes)

(4) Evaluation: Post-training assessment of current emotions and physical symptoms (CSEI-S, MRM, SUDS assessment) (5 minutes)

(5) Emotion regulation training (5 minutes)

The training phase is conducted through VR. For PTSD patients, training in Sangdanjeon meditation, corresponding to the Sasenggong (思勝恐) aspect of Oji Sangseung therapy, is primarily implemented. In addition to Sangdanjeon meditation, other training meditations can be implemented depending on the patient's emotional state. (about 20 minutes)

# 7. Observation items, observation inspection methods, and schedule

## 7.1 Progress Schedule

<Table 2> Progress Schedule

| **Period**  **Items** | | **screening** | **Test group: VR-based remote elevation therapy or remote elevation therapy** | | | | | **Follow**  **up** |
| --- | --- | --- | --- | --- | --- | --- | --- | --- |
| Week | | **-1** | **0** | **1~4** | **4** | **5~8** | **8** | **12** |
| Visit6) | | **Screening** | **1** | **2~7** | **8** | **9-15** | **16** | **17** |
| Control group Visit | | **Screening** | **1** |  | **2** |  | **3** | **4** |
| Obtain consent form | | ● |  |  |  |  |  |  |
| SCID-5 | | ● |  |  |  |  |  |  |
| PC-PTSD-5 | | ● |  |  |  |  |  |  |
| Demographic survey | | ● |  |  |  |  |  |  |
| Chest X-ray | | ● |  |  |  |  |  |  |
| Electrocardiogram | | ● |  |  |  |  |  |  |
| Laboratory tests7) | | ● |  |  |  |  | ● |  |
| Pregnancy diagnostic tests8) | | ● |  |  |  |  | ● |  |
| Vital signs | | ● | ● | ● | ● | ● | ● | ● |
| Medical history | | ● | ● | ● | ● | ● | ● | ● |
| Determination of inclusion/exclusion criteria | | ● |  |  |  |  |  |  |
| Randomization | |  | ● |  |  |  |  |  |
| Height/weight measurements9) | |  | ● |  |  |  | ● | ● |
| Concurrent medications | |  | ● | ● | ● | ● | ● | ● |
| Check for adverse reactions | |  | ● | ● | ● | ● | ● | ● |
| Baseline Questionnaire | Pattern identification tool for Jing Ji and Zheng Chong |  | ● |  |  |  |  |  |
| Sympto Evaluation Questionnaire | PCL-5-K |  | ● |  |  |  | ● | ● |
|  | CGI-I |  | ● |  |  |  | ● | ● |
|  | K-PTGI |  | ● |  |  |  | ● | ● |
|  | FFMQ-s |  | ● |  |  |  | ● | ● |
|  | PHQ-15 |  | ● |  |  |  | ● | ● |
|  | HADS |  | ● |  |  |  | ● | ● |
|  | ISI-K |  | ● |  |  |  | ● | ● |
|  | 화병척도 |  | ● |  |  |  | ● | ● |
| Economic evaluation | EQ-5D |  | ● |  | ● |  | ● | ● |
|  | EQ-VAS |  | ● |  | ● |  | ● | ● |
|  | 비용측정10) |  | ● |  | ● |  | ● | ● |
| Pre-Post Assessment | MRM |  | ● | ● | ● | ● | ● | ● |
|  | CSEI-S |  | ● | ● | ● | ● | ● | ● |
|  | SUDS |  | ● | ● | ● | ● | ● | ● |
| Device Exam | HRV |  | ● |  |  |  | ● |  |
|  | QEEG |  | ● |  |  |  | ● |  |
| Adherence Check | |  | ● | ● | ● | ● | ● |  |
| Visit Schedule Education | |  | ● | ● | ● | ● | ● |  |

6) Screening tests must be performed within 7 days prior to Visit 1, and laboratory test results must be available at Visit 1 (Week 0). Visit 1 is the Baseline visit.
7) Clinical pathology test, thyroid function test: Clinical trial subjects must fast for 2 hours before blood collection and undergo the following tests. Test results from within 14 days of Visit 1 are applicable, and retesting for abnormal results may be performed at the investigator's discretion
    Hematological Tests: WBC, RBC, Hemoglobin, Hematocrit, Platelet, MCV, MCH, MCHC, Diff Count (Neutrophil, Lymphocyte, Monocyte, Eosinophil, Basophil)
    Hematological Chemistry Tests: Total Bilirubin, ALP, AST, ALT, γ-GTP, Creatinine, Glucose, Globulin, Total Protein, Albumin, BUN, Total Cholesterol, Triglyceride, LDL Cholesterol, HDL Cholesterol, Na, K, Cl, T3, Free T4, TSH
    Urine Tests: Specific Gravity, pH, Leuko Esterase, Nitrite, Protein, Glucose, Ketone, Urobilinogen, Bilirubin, Erythrocyte, Microscopic Examination (RBC, WBC, Epi.cell, Crystal.cast, Other)
8) Urine HCG is performed only for premenopausal women, and the result must be negative. However, it is performed at Visit 1 during menstruation.
9) Height is measured only at Visit 1, and weight is measured and recorded at Visits 1, 16, and 17. Height is measured to the nearest 0.1 cm, and weight is measured to the nearest 0.1 kg.
10) Costs are measured using a cost questionnaire developed specifically for assessing direct medical costs, direct non-medical costs, and indirect costs.

ISI-K; Korean version of Insomnia severity index CSEI-S; The Core Seven-Emotions Inventory

Short Form

SUDS; Subjective Units of Distress Scale MRM; Mentalizing the Rooms of Mind EQ-5D; EuroQoL-5Dimension

EQ-VAS; EuroQoL-visual analog scale HRV; Heart Rate Variation

QEEG; Quantative Encephalogram

PCL-5-K: The	Posttraumatic	Stress	Disorder Checklist for DSM-5

K-PTGI;	Korea-	Posttraumatic	Growth Inventory-Short Form

CGI-I; The Clinical Global Impression–Improvement scale

FFMQ-S;	Five	Facet	Mindfulness	Questionnaire Short Form

PHQ-15; Patient Health Questionnaire-15

HADS; Hospital Anxiety and Depression Scale

## 7.2 Mehods by observation item

### 7.2.1 Subject Consent Form Signing and Screening Number Assignment

### - Prior to conducting this clinical trial, the investigator will explain the contents of the "Information for Obtaining Subject Consent" to the subject, confirm that the subject has fully understood the contents, and obtain the subject's written consent to participate in the clinical trial based on their own free will.

### - The following screening numbers will be assigned in the order of consent. The subject screening numbers will be recorded according to the following method: • Institution Code: Wonkwang University Sanbon Hospital (WS)

### • Institution Code - Year of Implementation - First Letter of Screening - Registration Order

### (e.g., WS-22-S-015: 15th patient screened in the first clinical trial at Wonkwang University Sanbon Hospital in 2022)

### 7.2.2 Demographic and Medical History Survey

### - Before starting the clinical trial, the subject's demographics, KCD disease codes, medical history, and medication history are reviewed through interviews and questionnaires. The results are recorded in the case record.

### 7.2.3 Subject Eligibility Assessment

### - The subject's eligibility for inclusion and exclusion criteria is assessed through demographic surveys, medical history surveys, and questionnaires.

7.2.4. Subject Identification Code Assignment

- Subject identification codes will be assigned only to individuals who consent to clinical trial participation and meet the inclusion and exclusion criteria through a subject eligibility assessment.

- Subject identification codes will be recorded according to the following method:

• Institution Code: Wonkwang University Sanbon Hospital (WS),

• Institution Code - Year of Implementation - Initial Letter of Enrollment - Order of Registration

(e.g., WS-22-E-015: 15th patient enrolled in the first clinical trial at Wonkwang University Sanbon Hospital in 2022)

7.2.5 Clinical Test Items

1) Clinical Pathology and Diagnostic Laboratory Tests

### (1) Clinical Pathology Tests

### - Hematology Tests

### : WBC, RBC, Hemoglobin, Hematocrit, Platelet, MCV, MCH, MCHC, Diff Count (Neutrophil, Lymphocyte, Monocyte, Eosinophil, Basophil), ESR

### - Hematology Tests

### : Total Bilirubin, ALP, AST, ALT, γ-GTP, Creatinine, Glucose, Globulin, Total Protein, Albumin, BUN, Total Cholesterol, Triglyceride, LDL Cholesterol, HDL Cholesterol, Na, K, Cl, T3, Free T4, TSH

### - Urine Tests: Specific Gravity, pH, Leuko Esterase, Nitrite, Protein, Glucose, Ketone, Urobilinogen, Bilirubin, Erythrocyte, Microscopic Examination (RBC, WBC, Epi.cell, Crystal Cast, Other)

### (2) Pregnancy Test

### - An HCG test is performed to determine the pregnancy status of female subjects of childbearing age. This test is limited to premenopausal women and must be negative. However, it is performed at visit 1 during menstruation.

### (3) Chest X-ray and Electrocardiogram

### - A chest X-ray and electrocardiogram are performed.

### (4) Vital Signs, Height, and Weight Measurement

### - Vital signs are measured at each visit. Height is measured at visit 1, and weight is measured and recorded at visits 1, 16, and 17.

### - Height is measured to the nearest 0.1 cm, and weight is measured to the nearest 0.1 kg.

2) Questionnaire and Assessment Items

(1) Borderline Stress Test

- The Borderline Stress Test developed by Park et al. [42] was used to analyze the subjects' stress patterns.

(2) Korean version of PTSD Checklist-5 (PCL-5-K)

- This 20-item self-report questionnaire was developed by Weathers et al. [43] to measure PTSD symptoms in the DSM-5. The PCL-5 is useful for individual screening and diagnostic assessment of PTSD and is suitable for monitoring changes in PTSD symptoms. Participants rate the level of distress caused by symptoms triggered by past stressful experiences over the past month on a Likert scale from 0 to 4, with a total score ranging from 0 to 80. If one or more symptoms in item B (questions 1-5), one or more symptoms in item C (questions 6-7), two or more symptoms in item D (questions 8-14), and two or more symptoms in item E (questions 15-20) of the PTSD diagnostic criteria are present, the possibility of PTSD increases, and the severity of symptoms is simultaneously evaluated with the total score of 20 items. Kim et al. [44] obtained permission to use the PCL-5 from the National PTSD Center in the United States, the copyright holder, and translated it into Korean (Korean version of PCL-5, PCL-5-K).

(3) The Clinical Global Impression – Improvement scale (CGI-I)[45][46][47]

- This scale assesses whether a patient's mental impairment has improved or worsened after a therapeutic intervention compared to before the intervention, and assigns a score from 1 to 7.

| Scale | Change | Scoring Guidelines |
| --- | --- | --- |
| 1 | 매우 많이 호전됨  (Very much improved) | - 거의 모든 것이 좋다(nearly all better) - 좋은 수준의 기능(good level of functioning) - 최소한의 증상(minimal symptoms) - 매우 실질적인 변화를 나타낸다(represents a very substantial change) |
| 2 | 많이 호전됨  (Much improved) | - 유의하게 증상이 현저히 감소하면 더 좋다(notably better with significant reduction of symptoms) - 기능 수준은 더 좋아졌지만, 일부 증상은 남아있다(increase in th level of functioning but some symptoms remain) |
| 3 | 약간 호전됨  (Minimally improved) | - 임상적으로 의미있는 증상 감소가 거의 없거나 전혀 없는 상태에서 약간 더 좋다(slightly better with little or no clinically meaningful reduction of symptoms) - 기본 임상 상태, 치료 수준 또는 기능적 능력에 거의 변화가 없음을   나타낸다(Represents very little change in basic clinical status, level of care, or functional capacity) |
| 4 | 변화가 없음  (No change) | - 증상은 본질적으로 변함이 없다(symptoms remain essentially unchanged) |
| 5 | 약간 악화됨  (Minimally worse) | - 약간 악화되었지만 임상적으로 의미가 없는 정도(slightly worse but may not be clinically meaningful) - 기본 임상 상태 또는 기능적 능력에 약간의 변화가 있을 수 있다   (may represent ver little change in basic clinical status or functional capacity) |
| 6 | 많이 악화됨  (Much worse) | - 임상적으로 유의한 증상의 증가와 기능 저하(clinically significant increase in symptoms and diminished functioning) |
| 7 | 매우 많이 악화됨  (Very much worse) | - 심각한 증상 악화 및 기능 상실(severe exacerbation of symptoms and loss of functioning) |

(4) Korean version of the Posttraumatic Growth Inventory (K-PTGI)

- This scale, developed by Tedeschi and Calhoun [48], measures posttraumatic growth, which refers to positive changes beyond the previous level of functioning after experiencing trauma. This study utilizes the Korean version of the PTGI, validated in Korea by Song Seung-hoon et al. [49]. The original scale consists of 21 items, but the Korean version consists of 16 items after removing five duplicate items during the validation process. The internal consistency (Cronbach's α) of the 16 items was .95. It consists of four subscales: changes in self-perception, increased depth of interpersonal relationships, discovery of new possibilities, and increased spiritual and religious interest. Each item is rated on a 6-point scale, ranging from 0 to 5.

### (5) Five Facet Mindfulness Questionnaire Short Form (FFMQ-S)

- This scale was developed based on the Five-Factor Mindfulness Questionnaire (FFMQ) developed by Baer et al. (2004)[50] and validated in Korea by Won Du-ri and Kim Kyo-heon (2006)[51]. This scale was designed to facilitate clinical comparisons of mindfulness before and after interventions. The Five-Factor Mindfulness Questionnaire Short Form (FFMQ-S) developed by Kang Hyung-won et al. (2017)[52] consists of 15 items and a 7-point scale. The internal consistency values reported by Kang Hyung-won et al. (2017) were .89 for non-automatism, .79 for awareness, .86 for description, .73 for non-judgment, .89 for observation, and .85 for the total score, indicating a reliable and valid scale.

Reliability of the 5-Factor Mindfulness Short Form Scale

| Subfactors | Question Number | Number of Quesions | Cronbach' alpha |
| --- | --- | --- | --- |
| Non-automatism | 1,5,15 | 3 | .888 |
| Awareness | 2,9,13 | 3 | .787 |
| Skill | 3,6,11 | 3 | .856 |
| Non-judgment | 4,12,14 | 3 | .725 |
| Observation | 7,8,10 | 3 | .891 |
|  | Total | 15 | .854 |

(6) Patient Health Questionnaire-15 (PHQ-15)

- This scale is primarily used to assess nonspecific somatic symptoms that appear to be psychologically related. It consists of 15 items, including stomach pain, back pain, joint pain, menstrual cramps, headache, chest pain, dizziness, feeling faint, racing heart, shortness of breath, painful intercourse, constipation or diarrhea, nausea, fatigue, and insomnia. Each item is scored on a 0-2 point scale, resulting in a total score of 0-30 [53][54].

(7) Hospital Anxiety and Depression Scale (HADS)

- This scale was developed by Zigmond et al. [55] to measure anxiety and depression in a short period of time. This study used a 14-item version of the instrument translated into Korean by Oh et al. [56]. The seven odd-numbered items are the anxiety subscale, and the seven even-numbered items are the depression subscale. Each item is rated on a 4-point scale (0-3), with higher scores indicating greater anxiety and depression. This simple screening test can easily determine whether a patient has anxiety or depression symptoms that require psychiatric intervention.

(8) Insomnia Severity Index (ISI-K)

- The ISI is an assessment tool for insomnia. This study used the Korean version of the Insomnia Severity Index (ISI-K), developed by Bastien et al. [57] and validated by Cho et al. [58]. It consists of seven items: 1) difficulty falling asleep, 2) difficulty maintaining sleep, 3) difficulty waking up, 4) satisfaction with sleep, 5) the extent to which sleep disturbance interferes with daily life, 4) the extent of quality of life impairment due to sleep disturbance, and 7) worry about insomnia. Each item is scored from 0 to 4, resulting in a total score of 28. Scores of 0-7 indicate normal sleep, 8-14 indicate borderline sleep, 15-21 indicate clinical insomnia (moderate), and 22-28 indicate clinical insomnia (severe) [59]. In the study by Bastien et al., the internal consistency coefficient (Cronbach’s α) was 0.74 [57], and in the validation study by Cho et al., the internal consistency coefficient (Cronbach’s α) was 0.92 [58].

(9) Hwabyung Scale

- Developed by Kwon Jeong-hye et al. [60] to assess the psychological and personality status of Hwabyung patients, this self-report scale for Hwabyung consists of 15 items (symptom scale, 0-60 points) reflecting the emotional and physical symptoms of Hwabyung and 16 items (personality scale, 0-60 points) reflecting enduring personality traits related to Hwabyung. A higher score indicates more severe symptoms. It is rated as 0 - "Not at all," 1 - "Not much," 2 - "Moderately," 3 - "Quite a bit," and 4 - "Completely."

(10) Health-Related Quality of Life Assessment Scale (EuroQoL-5 Dimension: EQ-5D)

- The EQ-5D was established and continuously developed by the EuroQol Group in 1987. The Korean EuroQoL-5 Dimension (KEQ-5D) is a multidimensional preference-based HRQOL measure that measures health-related quality of life in terms of utility. Recently, the EuroQol Group announced a new quality of life measurement tool that expands the number of domains in each EQ-5D-3L from three to five to address the shortcomings of the EQ-5D-3L. While the EQ-5D-3L could express a total of 243 health states, the EQ-5D-5L is expected to reduce the ceiling effect of the EQ-5D-3L, increase reliability and sensitivity, and improve technical richness by expressing 3,125 (=55) health states. In fact, a study comparing the two tools, EQ-5D-3L and EQ-5D-5L, targeting cancer patients at a domestic hospital reported that the EQ-5D-5L had a lower ceiling effect and higher validity than the EQ-5D-3L, and that the test-retest reliability was similar (Seon Ha Kim. 2012) [61]. Currently, the official Korean version of the EQ-5D-5L is provided by the EuroQol Group.

(11) Overall Health Status (EuroQoL-Visual Analogue Scale: EQ-VAS) Score [62]

- The EQ-VAS is a rating scale, a visual analogue scale represented by a 20-cm vertical line. 100 points represents the best imaginable health state, and 0 points represents the worst imaginable health state. A score between 0 and 100 is assigned for each health state. Scores obtained from the rating scale clearly indicate the order of health outcomes and also provide information on the intensity of preferences. However, rating scales are prone to measurement bias. Rating scales are generally recommended as an adjunct to other instruments (Korea Evidence-based Healthcare Collaborating Agency, 2013), and the EQ-VAS is also included as an adjunct to the EQ-5D-5L and EQ-5D-3L standardized questionnaires.

(12) Cost Measurement

- To assess whether there is a significant difference in the effectiveness of the experimental and control groups relative to their costs, direct medical costs, direct medical expenses, and lost productivity costs are investigated.

(13) Mentalizing the Rooms of Mind (MRM)

- Mentalizing the Rooms of Mind is a M&L psychotherapy technique that helps clients observe their minds more objectively by structuring, visualizing, and specifying the rooms of the mind, which are the source of both strength and pain in the present moment [63]. It is known to have high clinical value as a useful tool for objective progress observation, helping clients and therapists establish attitudes, responses, and a therapeutic relationship. It also aids in visualizing and specifying vague thoughts [64][65]. Participants are given A4 paper and a pencil, and the following instructions are provided.

From now on, let's represent **'s mind as a circle.

There are many rooms, large and small, within **'s mind.

There isn't just one room; it's made up of many.

There might be a large room, a small room, a triangular room, a memo room, a distorted room, a star room, or even a nameless room. There might also be a room of depression, a room of anxiety, a room of anger, a room of gratitude...etc.

Consider the circle drawn below as your mind.

Can you name each room that occupies **'s mind at this very moment?

Which room takes up the most space among these?

Each room, both difficult and other, has its own history and many things to say.

A state of observing each room with the eyes of an observer, a state of entering a room and observing it from a distance, without being removed from it. This is called a meditative state.

(14) The Core Seven-Emotions Inventory Short Form (CSEI-S) [66]

- Consists of 28 items, including 4 items on joy (喜), 4 items on anger (怒), 4 items on thought (思), 4 items on worry (憂), 4 items on sadness (悲), 4 items on fear (恐), and 4 items on surprise (驚). Each item is rated on a 5-point Likert scale: 1 = not at all, 2 = a little, 3 = somewhat, 4 = quite, 5 = very much. This scale is based on T-scores (mean 50, standard deviation 10). Except for joy (喜), higher scores for anger (怒), thought (思), worry (憂), sadness (悲), fear (恐), and surprise (驚) indicate higher risk. Specifically, T-scores of 55-60 indicate caution, 61-65 indicate risk, and 66 or higher indicate high risk. Conversely, for joy (喜), lower scores indicate higher risk: 40-45 indicate caution, 35-39 indicate risk, and 34 or lower indicate high risk.

- In particular, individuals with elevated levels of sadness (悲), worry (憂), and anger (怒)—emotions significantly higher in those with the disease than in those without—are assessed as requiring increased attention.

(15) Subjective Units of Disturbance Scale (SUDS)

- The Subjective Units of Disturbance Scale (SUDS) is a visualized version of the scale used by psychiatrist Joseph Wolff in the 1950s to measure the degree of pain or discomfort patients experience, using a scale from 0 to 10 [67].

- Specifically, the SUDS is divided into three categories: "0" represents no pain at all, a state of complete relaxation, and a state of complete comfort. "10" represents the worst state, where anxiety reaches the peak, beyond imagination, and a state where one feels as if one is about to fall into a state of panic and be overwhelmed.

(16) Heart Rate Variability (HRV) Measurement

- HRV analyzes the continuous changes in heart rate through autonomic nervous system regulation to maintain homeostasis, thereby measuring the balance between the sympathetic and parasympathetic nervous systems and their respective activity levels [68].

- Measurements are performed in a comfortable, quiet, and cool room, with the subject in a semi-recumbent position, resting and with eyes open. During the measurement, the subject is instructed to breathe normally without moving or speaking. Disc electrodes are attached to both wrists and left ankle, and the measurement is performed for 3 minutes.

- The results from the SA-3000P measurement device are checked. In the time domain, the Mean HR and SDNN values are measured, and in the frequency domain, the TP, LF, HF, and LF/HF values are measured.

(16) Quantitative Encephalogram (QEEG) Measurement

- In a comfortable, quiet, and cool room, the subject is semi-reclined, as awake as possible, and measured for 5 minutes with both eyes closed and eyes open.

- An electrodecap (Electro-cap; Mitsar, Russia) with 21 surface electrodes positioned according to the International 10/20 system is attached. All electrodes must have an impedance of 5 kΩ or less. The reference electrode is positioned between Fz and Cz, and a Mitsar-EEG 201 machine (Mitsar, Russia) is used as an amplifier. All signals are extracted using a high-frequency filter of 50 Hz, a low-frequency filter of 0.3 Hz, and a notch filter of 55–65 Hz.

- The EEG is recorded while the observer carefully monitors the subject's eye movements, muscle movements, or other artifacts. The first 500ms and the last 500ms of the measurement are removed, and artifacts are removed through automatic algorithms of WinEEG (http://www.mitsar-medical.com) and visual inspection**.** EEG segments with artifact removal exceeding 1 minute and split-half reliability exceeding 95% were used.

- Spectral analysis, coherence analysis, and LORETA analysis were performed using WinEEG. Spectral analysis utilized linked ears reference montage and local average (Laplacian) montage. Linked ears reference montage is generally used as a reference, while local average (Laplacian) montage has the advantage of highlighting local fields while minimizing the overall brain effects of drugs. Absolute power and relative power were calculated using a fast Fourier transform, with delta (0.5–4 Hz), theta (4–8 Hz), alpha (8–12 Hz), and beta (12–20 Hz), respectively.

(17) Structured Clinical Interview for DSM-5 (SCID-5)

- The SCID used in this study is the research version of the Structured Clinical Interview for DSM-5 Axis I Disorders (SCID). Compared to the clinical version, the research version allows for more detailed recording of disorder subtypes, severity, course specifiers, and past mood episodes. The SCID is designed for use with adults 18 years of age and older, but with some modifications, it can also be used with adolescents. However, individuals with severe cognitive impairment, anxiety, agitation, or severe psychotic symptoms may find the SCID difficult to interview. The decision to use the SCID is made in the introduction section at the beginning of the interview. Additionally, the introduction section can collect information on demographics, education, occupation, and treatment status.

(18) PC-PTSD-5 (The Primary Care PTSD Screen for DSM-5)

- The PC-PTSD-5 was developed to screen for PTSD in primary care settings in accordance with the DSM-5 diagnostic criteria [69]. It consists of five items, including four questions measuring re-experiencing, avoidance, numbing, and hyperarousal in the past month, plus an item on negative emotional changes, with responses of “yes” or “no.” In this study, subjects with a cutoff score of 3 or higher were included in the screening phase using the scale adapted by Park et al. (2016) [70].

### 7.2.5 Compliance Assessment

- Compliance with the intervention will be assessed as follows:

- Overall compliance throughout the trial period must be 75% or higher. If the compliance rate falls below 75%, the subject will be considered to have poor compliance and will be excluded from the PP analysis group.

Actual Number of Intervensions

Compliance =

Planned Number of intervension
× 100

7.2.6 Adverse Reaction Investigation

- The investigator will observe the subjects immediately prior to the end of the trial for any adverse reactions that may occur after participation in the clinical trial and record the causal relationship with the trial treatment in the "Adverse Reaction Record."

**7.3 Observational Test Methods and Schedule**

7.3.1 Selection Visit and Screening

- Applicants who apply for clinical trial participation in person or by phone will be screened according to the following procedure:

1) Obtain a signed informed consent form. Applicants participating in this trial will be provided with an explanation of the consent process and will be allowed to participate after voluntarily giving written consent on the informed consent form.

2) The following tests will be performed to determine eligibility as a clinical trial subject.

• Demographic information: Subject name, gender, date of birth, address, mobile phone number

• Subjects diagnosed with PTSD using the Structured Clinical Interview for DSM-5 (SCID-5)

• Subjects scoring 3 or higher on the PC-PTSD-5

• Interview and medical history: Current medical history, past medical history, smoking status, alcohol consumption, treatment history, medication use

• Physical findings: Blood pressure, pulse, body temperature, respiration, weight, height

• Chest X-ray, electrocardiogram

• Clinical pathology tests, pregnancy test

2) Subjects will be screened to determine eligibility for the study and meet the exclusion criteria. If eligible for participation, a subject identification code will be assigned based on the randomization and recorded in the case record.

7.3.2 Study Implementation

- Subjects will be divided into study group 1, study group 2, and control group according to randomization.

If assigned to the control group, the following measures will be taken:

- Initial (baseline) ① Baseline questionnaire (Borderline Confusion Scale), ② Symptom Assessment Questionnaire (PCL-5-K, CGI-I, K-PTGI, FFMQ-S,PHQ-15, HADS, ISI-K Hwabyung Scale), ③ Economic evaluation (EQ-5D, EQ-VAS, cost measurement), ④ Pre- and Post-evaluation (MRM, CSEI-S, SUDS), and ⑤ Instrumental testing (HRV, QEEG).

- Subjects will be instructed to carry out their normal daily activities for 8 weeks, and to minimize contact with the clinical trial investigator, they will be contacted by phone every two weeks to check their status.

- Four weeks later (after 8 sessions in the experimental group), an economic evaluation (EQ-5D, EQ-VAS, cost measurement) will be conducted. - After 8 weeks (after the final completion of the 16th trial in the experimental group), ① the Symptom Assessment Questionnaire (PCL-5-K, CGI-I, K-PTGI, FFMQ-S, PHQ-15, HADS, ISI-K Hwabyung Scale), ② the Economic Evaluation (EQ-5D, EQ-VAS, Cost Measurement), ③ the Pre- and Post-evaluation (MRM, CSEI-S, SUDS), and ④ the Instrumental Evaluation (HRV, QEEG) will be administered.

- At the 12-week follow-up, the control group will be administered the following: ① the Symptom Assessment Questionnaire (PCL-5-K, CGI-I, K-PTGI, FFMQ-S, PHQ-15, HADS, ISI-K Hwabyung Scale), ② the Economic Evaluation (EQ-5D, EQ-VAS, Cost Measurement), and ③ the Pre- and Post-evaluation (MRM, CSEI-S, SUDS). After all tests are completed, VR-based remote elevation therapy is performed twice a week for 8 weeks for 8 sessions, if desired.

### B. If eligible for the test group, the following clinical trial will be conducted.

### - Test Group 1 will receive VR-based remote elevation therapy, and Test Group 2 will receive remote elevation therapy.

### - At baseline, ① a baseline questionnaire (Borderline Confusion Scale), ② a symptom assessment questionnaire (PCL-5-K, CGI-I, K-PTGI, FFMQ-S,

### PHQ-15, HADS, ISI-K Hwabyung Scale), ③ an economic evaluation (EQ-5D, EQ-VAS, cost assessment), ④ a pre- and post-evaluation (MRM, CSEI-S, SUDS), and ⑤ instrumental testing (HRV, QEEG).

### - After 4 weeks (after 8 sessions in the test group), an economic evaluation (EQ-5D, EQ-VAS, cost assessment) will be conducted. - After 8 weeks (after the final completion of the 16th trial group), ① symptom assessment questionnaires (PCL-5-K, CGI-I, K-PTGI, FFMQ-S, PHQ-15, HADS, ISI-K Hwabyung Scale), ② economic evaluation (EQ-5D, EQ-VAS, cost measurement), ③ pre- and post-evaluations (MRM, CSEI-S, SUDS), and ④ instrumental testing (HRV, QEEG) will be administered.

### - After 12 weeks, follow-up measurements will include ① symptom assessment questionnaires (PCL-5-K, CGI-I, K-PTGI, FFMQ-S, PHQ-15, HADS, ISI-K Hwabyung Scale), ② economic evaluation (EQ-5D, EQ-VAS, cost measurement), and ③ pre- and post-evaluations (MRM, CSEI-S, SUDS). After all trials are completed, VR-based orthopedic therapy is administered twice a week for eight weeks, for eight sessions, if desired. - MRM, CSEI-S, and SUDS are administered before and after each session. Adverse reactions are observed after each session and during the 12-week follow-up.

7.3.2.1 Session 1 (Start of Intervention)

- The following assessments will be conducted within 7 days of the selection visit (selection will be confirmed by phone).

① The results of the selection visit, including chest X-rays, electrocardiograms, and clinical pathology tests, will be reviewed.

② Any changes in the subject's medical history or medications after the selection visit will be reviewed and recorded.

③ Blood pressure, pulse, body temperature, respiratory rate, height, and weight will be measured.

④ Before the intervention, baseline questionnaires (Borderline Severity Index), symptom assessment questionnaires (PCL-5-K, CGI-I, K-PTGI, FFMQ-s, PHQ-15, HADS, ISI-K Hwabyung Scale), economic evaluations (EQ-5D, EQ-VAS, cost measurement), pre- and post-evaluations (MRM, CSEI-S, SUDS), and instrumental assessments (HRV, QEEG) will be measured and recorded. ⑤ Test Group 1 will receive VR-based 40-minute upright positioning therapy, while Test Group 2 will receive 40-minute upright positioning therapy.

⑥ The MRM (Mapping the Next Room), CSEI-S, and SUDS will be assessed before and after the intervention.

⑦ The next visit date will be designated.

7.3.2.2 Sessions 2-7 (Intervention)

① Measure blood pressure, pulse, body temperature, and respiratory rate.

② Before the intervention, assess the Mind Map (MRM), CSEI-S, and SUDS.

③ Test Group 1 receives VR-based 40-minute

7.3.2.3 Session 8 (Intervention)

① Measure blood pressure, pulse, body temperature, and respiratory rate.

② Before the intervention, assess the Mind Map (MRM), CSEI-S, and SUDS.

③ Test Group 1 receives VR-based 40-minute

7.3.2.4 Sessions 9-15 (Intervention)

① Measure blood pressure, pulse, body temperature, and respiratory rate.

② Before the intervention, assess the Mind Map (MRM), CSEI-S, and SUDS.

③ Test Group 1 receives VR-based 40-minute ...

### 7.3.2.5 Session 16 (Intervention)

### ① Measure blood pressure, pulse, body temperature, respiratory rate, and weight.

### ② Before the intervention, assess the Mind Room Drawing (MRM), CSEI-S, and SUDS.

### ③ Test Group 1 receives VR-based euphoric therapy for 40 minutes, while Test Group 2 receives euphoric therapy for 40 minutes.

### ④ After the intervention, perform and record the MRM, CSEI-S, SUDS, symptom assessment questionnaires (PCL-5-K, CGI-I, K-PTGI, FFMQ-s, PHQ-15, HADS, ISI-K Hwabyung Scale), economic evaluation (EQ-5D, EQ-VAS, cost measurement), and instrumental assessments (HRV, QEEG).

### ⑤ Check for concomitant medications, adverse reactions, and compliance.

### ⑥ Set the next visit date.

7.3.2.6 17th Visit (Week 12 Follow-up Visit)

The following questionnaires will be administered at the Week 12 visit.

① Blood pressure, pulse, body temperature, respiratory rate, and weight will be measured.

② Adverse events and unusual findings will be identified and recorded after the intervention.

③ The MRM (Mind Room Drawing), CSEI-S, SUDS, symptom assessment questionnaires (PCL-5-K, CGI-I, K-PTGI, FFMQ-s, PHQ-15, HADS, ISI-K Hwabyung Scale), and economic evaluation (EQ-5D, EQ-VAS, cost measurement) will be administered and evaluated.

④ The clinical trial will be concluded.

7.3.2.6 Additional Visits

- Additional visits may be conducted at any time, outside of the scheduled visits, at the subject's request or at the investigator's discretion, as deemed necessary. If a subject visits on an unscheduled day, adverse reactions, concomitant medications, test results, and any subsequent medical treatment should be recorded in the case record. Even for subjects who drop out, additional visits may be conducted to conduct clinical laboratory tests, pregnancy tests, and efficacy assessment tests.

# 8. Expected Side Effects

**-** This VR-based otolithiasis treatment may cause the following side effects, but they are minimal. You can temporarily stop using it and then resume using it, or check your condition before deciding whether to use it again.

• Side effects such as mild headache/eye fatigue/eye or muscle spasms/involuntary eye movements/nausea/dizziness/discomfort or pain in the head or eyes/drowsiness/fatigue/infectious symptoms of the eyes, skin, or scalp/skin irritation may occur..

# 9. Criteria for suspension and elimination

9.1 Definition of Discontinuation

- Discontinuation is defined as the suspension of a clinical trial when it cannot proceed due to adverse events, adverse reactions, etc., or when the trial threatens the safety of the subjects.

9.2 Criteria for Discontinuation

1) When adverse reactions occur

2) When the investigator determines that the trial is not appropriate to proceed

9.3 Definition of Withdrawal

- A subject is defined as having completed the trial when he or she has completed the training and pre- and post-training observations as specified in the protocol. A subject is considered to have withdrawn from the trial if he or she fails to complete the trial due to adverse reactions or other reasons.

9.4 Criteria for Withdrawal

- The investigator may discontinue treatment and observation and withdraw the patient, and the subject may withdraw from the study at any time at his or her own discretion.

- Criteria for Withdrawal are as follows:

1) Subjects who fail to comply with the investigator's instructions

2) Protocol violations

If the subject or investigator fails to comply with the protocol

3) Serious adverse reactions occur

4) Compliance is below 75%

5) Other

If the subject withdraws consent or requests discontinuation of treatment

9.5 Process for Discontinuation and Withdrawal

- The date, time, and reason for a subject's withdrawal or discontinuation must be recorded in the termination report. Every effort must be made to follow-up subjects who withdraw or discontinue for any reason, and a safety follow-up assessment must be conducted within one week of treatment initiation. Subjects may voluntarily withdraw from the study at any time for any reason and are not required to provide a reason for withdrawal. However, the investigator must record the reason for withdrawal or discontinuation, or any failure to identify the reason, in the termination report and make every effort to follow-up the subject who has withdrawn. The investigator also has the option to exclude the subject from the study.

- If a subject discontinues participation due to adverse events or other reasons during the clinical trial, unexpected additional costs may arise. Therefore, an economic evaluation case record questionnaire may be conducted to collect information on the items and amounts of such costs.

9.6 Handling Compliance and Protocol Violations

- The clinical trial director and staff must be fully familiar with and thoroughly implement the protocol to prevent any violations. The investigators must take appropriate measures, such as mobile phone text messages, telephone monitoring, written notifications, and emails, to ensure subject compliance with visit and testing schedules. Unavoidable protocol violations will be handled as follows:

- In the case of serious protocol violations, such as violations of inclusion/exclusion criteria, failure to obtain informed consent, or use of medications that may affect the clinical trial results, the subject's information will be excluded from analysis.

- For other minor violations, the degree and reason for the violation or delay must be accurately described and analyzed to assess whether it affected the clinical trial results.

# 10. Evaluation criteria and methods

10.1. Evaluation Criteria

10.1.1 Pre- and Post-Analysis of VR-Based Ojiseungseung Therapy and Ojiseungseungseung Therapy

- Conduct an evaluation analysis of the primary efficacy questionnaire.

- Conduct an evaluation analysis of the secondary efficacy questionnaire.

10.1.2 Quantitative Electroencephalography (QEEG) Analysis

① Frequency spectral analysis:

- Compare changes in absolute power (μV2) and relative power (%) for each frequency band before and after OAS and VR-based OAS.

- Compare changes in absolute power and relative power z-scores using normative databases before and after OAS and VR-based OAS.

② Source localization-LORETA (low-resolution brain electromagnetic tomography):

- Estimate current densities in cortical regions at each frequency using LORETA (low-resolution brain electromagnetic tomography). Compare changes in Brodmann areas corresponding to activated cortical regions before and after OAS and VR-based OAS.

# 11. Statistical analysis methods

11.1. Analysis of Demographic Data and Clinical History at Trial Entry

- Descriptive Statistics Analysis

The demographic data (demographic characteristics) and clinical history at trial entry (hereinafter referred to as "baseline data") of the subjects included in this clinical trial are presented as continuous data, including mean, standard deviation, minimum, and maximum values. For categorical data, frequency tables are provided. Descriptive statistics and frequency analysis are performed for this analysis.

- Normality Verification

The normality of the observed endpoints at each measurement point is verified. a. If normality is satisfied, parametric tests are performed. b. If normality is not satisfied, nonparametric tests are performed.

11.2. Analysis Methods for Evaluation Variables

11.2.1. Effectiveness Analysis of VR-Based Ojiseunghwa Therapy and Ojiseunghwa Therapy

1. Difference Test

(1) Main Effectiveness Evaluation Measurement Tools

① Descriptive statistics (mean, standard deviation, median, etc.) will be analyzed for PCL-5-K measures for subjects in groups 1 and 2 and the control group before and after the intervention (week 0, session 1 - week 8, session 16) and at the 12th visit.

② After analyzing the descriptive statistics for PCL-5-K measures for subjects in groups 1 and 2 and the control group, if the pre- and post-intervention measurements meet the normality test, a parametric difference test will be performed. If the normality test is not met, a nonparametric test will be performed.

A. Parametric Test

- When testing for differences within each group, a paired-samples t-test is conducted for the first session of Week 0 and the 16th session of Week 8 (before and after the intervention).

- When testing for differences between the three groups (VR-based remote elevation therapy group, remote elevation therapy group, and control group), a parametric test, ANOVA (one-way analysis of variance), is conducted, followed by a post-hoc test (Scheffe, Dunnett T3 or Games-Howell if equal variances are not assumed).

B. Nonparametric Test

- When normality is not met, a nonparametric Wilcoxon-Mann-Whitney U test is conducted for the first session of Week 0 and the 16th session of Week 8 (before and after the intervention). If normality is not met, a nonparametric ANOVA (Kruskal-Wallis test) will be performed between the three groups (test group 1 and 2, control group), and the degrees of freedom, test statistic (H), and significance level will be reported. Furthermore, a nonparametric post hoc test will be performed to analyze the rank distribution between groups using Friedman.

Through this analysis, we will analyze differences and changes between groups and verify the differential effectiveness of VR-based Oji-seung-hyeop treatment and Oji-seung-hyeop treatment.

(2) Second Efficacy Assessment Measurement Tools

① Descriptive statistical analysis will be conducted on the scores from the floating efficacy assessment questionnaires (CGI-I, CSEI-S, K-PTGI, FFMQ-S, PHQ-15, HADS, ISI-K, Hwabyung Scale, and subjective discomfort) and instrumental tests (QEEG, HRV) for study subjects in groups 1 and 2 and the control group.

② For each floating efficacy assessment scale (CGI-I, CSEI-S, K-PTGI, FFMQ-S, PHQ-15, HADS, ISI-K, Hwabyung Scale, and subjective discomfort) and instrumental tests (QEEG, HRV), a within-group paired t-test and a between-group difference test will be conducted for the three groups (VR Ojisangseung Position Treatment Group, Ojisangseung Position Treatment Group, and Control Group) on the pre- and post-test scores. At this time, the intra-group difference test is performed through normality verification, and if normality is satisfied, parametric verification is performed. If normality is not satisfied, a non-parametric verification is performed and a paired sample T-test is performed.

A. Parametric Test

- When testing for differences within each group, a paired-samples t-test will be conducted on the validity assessment questionnaires from Week 0, Session 1, and Week 8, Session 16 (before and after the intervention).

- When testing for differences between the three groups (VR-based remote ascent therapy group, remote ascent therapy group, and control group), a parametric test, one-way analysis of variance (ANOVA), will be conducted, followed by post hoc tests (Scheffe, Dunnett T3, or Games-Howell if equal variances are not assumed).

B. Nonparametric Test

- If normality is not met, a nonparametric Wilcoxon-Mann-Whitney U test will be performed on each sub-validation questionnaire to verify within-group differences between Week 0, Session 1, and Week 8, Session 16 (before and after the intervention).

- If normality is not met, a nonparametric ANOVA, the Kruskal-Wallis test, will be performed on each sub-validation questionnaire between the three groups (experimental groups 1 and 2, control group). The degrees of freedom, test statistic (H), and significance level will be reported. Furthermore, a nonparametric post-hoc test will be performed to analyze the rank distribution between groups using Friedman.

- Through this analysis, differences and changes between groups will be analyzed to verify the effectiveness of VR-based Ojisangseung treatment and Ojisangseung treatment.

③ Quantitative Electroencephalography (QEEG) Analysis

a. Frequency spectral analysis:

- Based on the baseline, descriptive statistics (mean, standard deviation) were analyzed for absolute power (μV2) and relative power (%) for each electrode location in each frequency band for all subjects in the test groups 1 and 2 and the control group.

b. Source localization-LORETA (low-resolution brain electromagnetic tomography): Frequency bands and Brodmann areas were distinguished, and differences in brain activation areas between LORETA (low-resolution brain electromagnetic tomography) sessions 1 and 16 were analyzed at a p<.05 level.

11.2.2. Correlation Analysis between Pre- and Post-Original and VR-Based Original Therapy Measurements

After verifying the normality of the Primary and Secondary Validity Scales (PCL-5-K) for psychotherapy, a parametric (Pearson) correlation analysis was performed if normality was met. If normality was not met, a non-parametric Spearman correlation analysis was performed.

11.2.3. Economic Evaluation

- The economic evaluation is primarily conducted using FAS analysis, with a PP analysis performed concurrently to assess sensitivity to missing data. Cost and utility estimation in economic evaluations is conducted in the same manner as clinical trial effectiveness estimation, with an analysis of the mechanisms underlying missing data and an appropriate imputation method selected.
- The economic evaluation, conducted concurrently with the clinical trial, aims to determine the cost-effectiveness of the treatment under study versus conventional care. The primary economic endpoint is Quality Adjusted Life Years (cost per QALY) gained, calculated using the EQ-5D. Secondary endpoints include effectiveness indicators, such as cost per EQ-VAS, calculated using the EQ-VAS. - The first analysis period is 8 weeks (total follow-up period), and if an estimate for the subsequent period is required, a secondary analysis is conducted by extrapolating the costs and effects after the follow-up period using a regression model, etc., or by performing decision modeling analysis, etc. Treatment costs incurred in connection with clinical trials are calculated by combining the number of treatments and unit costs. Treatment costs incurred within the clinical trial institution are recorded by researchers after breaking the blind and examining the electronic data. Quality of life estimation for calculating QALY uses quality of life derived from the EQ-5D as the primary evaluation variable, and the calculation method is the Area under the curve method (Brazier J, Ratcliffe J, Salomon JA, Tsuchiya A: Measuring and Valuing Health Benefits for Economic Evaluation. Oxford, UK: Oxford University Press; 2007.).
- If the total analysis period (Time Horizon) is extrapolated beyond the clinical trial period and exceeds 12 months, the cost unit will be standardized to the 2021 Korean Won (KRW), and a 5% discount rate will be applied based on the Health Insurance Review and Assessment Service's economic evaluation guidelines.
- This study focuses on a societal perspective. Baseline analysis is conducted using representative values (e.g., means) of the parameters used in the study. Sensitivity analysis is conducted using a probabilistic sensitivity analysis using the distributions and representative values of all possible estimated parameters.
- The analysis results are presented in tables, including the incremental cost-effectiveness ratio, a cost-effectiveness plane with confidence intervals identified using nonparametric methods, a cost-effectiveness acceptability curve (CEAC) that demonstrates the sensitivity of cost-effectiveness to changes in the national threshold, and a value-of-information analysis graph that estimates the value of information for the target population. In this study, statistical analysis was conducted after verifying the normality of all continuous variables using the Shapiro-Wilk test, etc., when the sample size was 30 or less. The statistical significance level was tested at a p-value of 0.05. The analysis program was Stata 14 MP version and R program (4.0.2 version). In cases where extrapolation through modeling was performed, the study was conducted using Treeage Pro 2016.

11.2.4. Qualitative Evaluation

- A qualitative evaluation of the MRM will be conducted for the VR Ojisangeuphyanggi treatment group, the Ojisangeuphyanggi treatment group, and the control group.

- The MRM is a type of projective test, and unlike objective assessments, it analyzes qualitative content.

- A clinical psychologist and a Korean medicine neuropsychiatrist qualified to evaluate projective psychological tests will conduct the projective evaluation.

11.3. Analysis Methods for Safety Variables

- List all adverse reactions that occurred, along with detailed descriptions. Record the frequency of adverse reactions related to VR-based or non-VR-based treatment, as well as those unrelated to VR-based treatment.

- For adverse reactions, use the McNemar test to determine whether there is a significant difference in the total frequency of adverse reactions or individual frequencies. If the severity (or level) of adverse reactions is being investigated, use a paired t-test or the Wilcoxon-Mann-Whitney U test.

# 12. Safety evaluation criteria, evaluation methods, and reporting methods, including adverse reactions

- Subject Safety

This study aimed to evaluate the safety, efficacy, and economic feasibility of a virtual reality-based euphoric therapy for patients with post-traumatic stress disorder (PTSD). The intervention, euphoric therapy, is a treatment currently being used in clinical practice. The VR-based euphoric therapy utilizes a virtual reality device only during the training phase of existing treatments. Medically, the additional risks associated with this study are not significant compared to existing procedures or treatments. Therefore, no additional physical or mental harm or specific injury is expected to occur to the subjects. Furthermore, the device used in this clinical trial is noninvasive and the duration of the trial is short, so there are no safety risks.

12.1 Definition of Safety-Related Terms

12.1.1 Adverse Events

- An adverse event (AE) refers to an undesirable and unintended sign, symptom, or disease that occurs in a subject during a clinical trial. It does not necessarily have a causal relationship with the treatment used in the clinical trial.

12.1.2 Serious Adverse Reactions

- Serious adverse reactions (AEs) are adverse reactions occurring due to acupuncture treatment used in clinical trials that fall under any of the following categories:

-Caused death or life-threatening

-Requiring hospitalization or an extension of hospitalization

-Caused persistent or significant disability or functional decline

-Caused congenital malformations or abnormalities

## 12.2 Evaluation of adverse reactions

12.2.1 Severity Assessment

If an adverse reaction occurs, it must be reported according to the following severity assessment criteria.

Mild: Does not interfere with the subject's normal daily life (function), causes minimal discomfort, and is easily tolerated by the subject.

Moderate: Causes discomfort that significantly interferes with the subject's normal daily life (function).

Severe: Makes the subject's normal daily life (function) impossible.

12.2.2 Assessment of Causality with the Test Treatment

The investigator will assess the causality of the adverse reaction to the treatment based on the following criteria and describe the investigator's opinion.

1) Definitely related

- If the temporal sequence of the adverse reaction and the VR-based or VR-based treatment is reasonable.

- If the adverse reaction is most likely explained by the VR-based or VR-based treatment.

- If the adverse reaction disappears upon discontinuation of the VR-based or VR-based treatment.

- If the adverse reaction is consistent with previously known information about the VR-based or VR-based treatment.

2) Probably related

- If the temporal sequence of the adverse reaction and VR-based or VR-based treatment is reasonable.

- If the adverse reaction is more likely to be explained by VR-based or VR-based treatment than by other causes.

- If the adverse reaction disappears upon discontinuation of VR-based or VR-based treatment.

2) Possibly related

- If the temporal sequence of the adverse reaction and VR-based or VR-based treatment is reasonable.

- If the adverse reaction is judged to be at least as likely to be attributed to VR-based or VR-based treatment as to other possible causes.

- If the adverse reaction disappears with VR-based or discontinuation of oriental escalation therapy (if implemented).

3) Probably not related

- If there is a more probable cause for the adverse reaction.

- If the results of VR-based oriental escalation therapy discontinuation (if implemented) are negative or ambiguous.

- If the results of VR-based oriental escalation therapy (if implemented) are negative or ambiguous.

4) Definitely not related

- If the temporal sequence between VR-based oriental escalation therapy and the adverse reaction onset is not plausible.

- If there is another clear cause for the adverse reaction.

5) Unknown

12.3 Safety Statistical Analysis

- Follow the statistical analysis method in Section 11.3.

12.4 Adverse Event Reporting

12.4.1 Adverse Event Education

The study principal investigator shall provide training to study staff, subjects, or guardians regarding all adverse events that may occur after VR-based or OA treatment, and to ensure that all adverse events occurring after treatment are reported.

12.4.2 Reporting of Serious Adverse Events

All serious adverse events occurring during the study, regardless of whether they are related to VR-based or orthostatic hypotensive therapy, must be reported to the person listed in the contact information section below within 24 hours, and no later than the next business day. Additionally, any event deemed serious by the investigator or suggesting significant risks, contraindications, side effects, or precautions that may be associated with the use of VR-based or orthostatic hypotensive therapy, must also be recorded as a serious adverse event..

| 성명 | 소속 | 연락처 |
| --- | --- | --- |
| 강형원 | 원광대학교 산본병원 | 031-390-2763 |

The following information should be provided in the final report, if available: time of onset, severity, treatment, course, and causal relationship to VR-based or VR-based terrestrial elevation therapy.

12.4.3 Actions to Take in the Event of an Adverse Reaction

- During this trial, the principal investigator and study staff must ensure patient safety. In the event of an unexpected serious adverse reaction, they must take prompt and appropriate measures to minimize the adverse reaction.

- In the event of a "serious adverse reaction" during a clinical trial, the duties of each staff member are as follows:

Obligations of the Principal Investigator

If a serious adverse reaction occurs during a clinical trial, the Principal Investigator must immediately report it to the Institutional Review Board and suspend all or part of the clinical trial for the VR-based orientated ascending-upward therapy or the orientated ascending-upward therapy until further instructions are provided.

Obligations of the Principal Investigator

If a serious adverse reaction occurs during the clinical trial, the Principal Investigator must immediately report it to the Principal Investigator and the Institutional Review Board.

Obligations of the Sponsor

If the Sponsor receives a report of a serious adverse reaction from the Principal Investigator or investigator, the Sponsor must attach a copy of the report submitted by the Principal Investigator or investigator to the Serious Adverse Reaction Report

and retain it, ensuring that appropriate action is taken immediately.

### 12.4.4 Follow-up of Adverse Reactions

### Investigators must monitor subjects who experience adverse reactions until the symptoms resolve and their condition stabilizes. Upon request by the sponsor, investigators must submit a report on the subsequent progression of adverse reactions.

# 13.Benefits and Benefits to Subjects

### - Through the study, the research can contribute to the scientific development of VR-based Ojiseungsanggi therapy and its effectiveness, and can contribute to the development of national healthcare policies. While subjects participate in the study, examination and training fees are free, and a small transportation allowance (KRW 50,000) is provided for each visit. However, while the control group's VR-based Ojiseungsanggi therapy is free of charge after the clinical trial, transportation costs are not covered..

14. Measures to Protect Subject Safety

- This trial is conducted in accordance with the guidelines of Good Clinical Practice and the Declaration of Helsinki.

14.1 Role of the Investigator

- The "Investigator" refers to the principal investigator and the study staff. The investigator must conduct the clinical trial in compliance with the protocol. During and after the clinical trial, the investigator must ensure that subjects receive appropriate medical treatment for all adverse reactions occurring during the clinical trial, including clinically significant abnormalities in clinical pathological tests. If the investigator becomes aware of any concomitant diseases requiring medical treatment, the investigator must inform the subject. The investigator must accurately analyze and understand the clinical trial plan and proactively address any concerns raised by the subject.

14.2 Role of Monitoring

- Monitoring oversees the progress of a clinical trial and reviews and confirms that the trial is conducted and recorded in accordance with the protocol, standard operating procedures, clinical trial management practices, and relevant regulations.

- Clinical trial monitoring is conducted through regular visits and telephone calls by the monitoring staff. During these visits, the monitoring staff checks original patient records and archived data (study files). Furthermore, the monitoring staff closely monitors the progress of the clinical trial and consults with the investigator if any issues arise.

14.3 Subject Informed Consent

- Before deciding whether to participate in a clinical trial, the subject must receive all relevant information through a written informed consent document. The informed consent document must be signed and dated to confirm the subject's voluntary participation.

- If the subject or their legal representative is unable to read the consent form, the informed consent document, or other written information, an impartial witness must be present throughout the entire consent process.

- Before obtaining consent, the investigator must provide the subject or their legal representative with sufficient time and opportunity to inquire about the details of the clinical trial and decide whether to participate. All questions related to the clinical trial must be answered to the subject or their legal representative's satisfaction.

14.4 Approval and Amendments to the Clinical Trial Plan

- The clinical trial plan must be reported to and approved by the Institutional Review Board.

- The clinical trial plan must be amended if the trial procedure becomes more extensive, the risk level increases, patient selection criteria change, or additional safety information becomes available. When amending the clinical trial plan, the date, reason, and details of the amendment must be recorded and maintained, and the amendment must be reported to the Institutional Review Board.

- The investigator must not conduct the clinical trial in a manner that deviates from the protocol, except when necessary to eliminate an immediate risk to the subjects.

14.5 Confidentiality of Subject Records

- Records that identify subjects will be kept confidential, and subject identities will remain confidential even if the clinical trial results are published.

- The sponsor, monitors, and inspectors involved in this trial may access subject records for the purposes of monitoring, reviewing, and managing the progress of this trial. By signing this protocol, the investigator acknowledges that the sponsor or the contract research organization's monitors and inspectors may review or copy subject charts and case record records for verification purposes, in accordance with domestic laws and ethical standards. This information must be kept confidential, and facilities and management standards for confidential storage must be in place.

- All documents related to the clinical trial, including case record forms, must be recorded and identified by subject identification code (typically the subject's initials), not the subject's name.

14.6 Data Retention

- A separate storage area must be established to securely preserve all clinical trial-related data and records, and security must be maintained. After the completion of the clinical trial report, a person responsible for archiving clinical trial-related documents must be designated to ensure their preservation for three years from the trial completion date.

15) **References**
